# Single-molecule analysis of DNA-binding proteins from nuclear extracts (SMADNE)

**DOI:** 10.1093/nar/gkad095

**Published:** 2023-03-02

**Authors:** Matthew A Schaich, Brittani L Schnable, Namrata Kumar, Vera Roginskaya, Rachel C Jakielski, Roman Urban, Zhou Zhong, Neil M Kad, Bennett Van Houten

**Affiliations:** Department of Pharmacology and Chemical Biology, University of Pittsburgh School of Medicine, Pittsburgh, PA, USA; UPMC-Hillman Cancer Center, Pittsburgh, PA, 15232, USA; UPMC-Hillman Cancer Center, Pittsburgh, PA, 15232, USA; Molecular Biophysics and Structural Biology Program, University of Pittsburgh, Pittsburgh, PA, USA; UPMC-Hillman Cancer Center, Pittsburgh, PA, 15232, USA; Molecular Genetics and Developmental Biology Graduate Program, University of Pittsburgh School of Medicine, Pittsburgh, PA, USA; UPMC-Hillman Cancer Center, Pittsburgh, PA, 15232, USA; Department of Pharmacology and Chemical Biology, University of Pittsburgh School of Medicine, Pittsburgh, PA, USA; UPMC-Hillman Cancer Center, Pittsburgh, PA, 15232, USA; School of Biosciences, University of Kent, Kent, UK; Department of Pharmacology and Chemical Biology, University of Pittsburgh School of Medicine, Pittsburgh, PA, USA; UPMC-Hillman Cancer Center, Pittsburgh, PA, 15232, USA; LUMICKS, Waltham, MA, USA; School of Biosciences, University of Kent, Kent, UK; Department of Pharmacology and Chemical Biology, University of Pittsburgh School of Medicine, Pittsburgh, PA, USA; UPMC-Hillman Cancer Center, Pittsburgh, PA, 15232, USA; Molecular Genetics and Developmental Biology Graduate Program, University of Pittsburgh School of Medicine, Pittsburgh, PA, USA; Molecular Biophysics and Structural Biology Program, University of Pittsburgh, Pittsburgh, PA, USA

## Abstract

Single-molecule characterization of protein–DNA dynamics provides unprecedented mechanistic details about numerous nuclear processes. Here, we describe a new method that rapidly generates single-molecule information with fluorescently tagged proteins isolated from nuclear extracts of human cells. We demonstrated the wide applicability of this novel technique on undamaged DNA and three forms of DNA damage using seven native DNA repair proteins and two structural variants, including: poly(ADP-ribose) polymerase (PARP1), heterodimeric ultraviolet-damaged DNA-binding protein (UV-DDB), and 8-oxoguanine glycosylase 1 (OGG1). We found that PARP1 binding to DNA nicks is altered by tension, and that UV-DDB did not act as an obligate heterodimer of DDB1 and DDB2 on UV-irradiated DNA. UV-DDB bound to UV photoproducts with an average lifetime of 39 seconds (corrected for photobleaching, τ_c_), whereas binding lifetimes to 8-oxoG adducts were < 1 second. Catalytically inactive OGG1 variant K249Q bound oxidative damage 23-fold longer than WT OGG1, at 47 and 2.0 s, respectively. By measuring three fluorescent colors simultaneously, we also characterized the assembly and disassembly kinetics of UV-DDB and OGG1 complexes on DNA. Hence, the SMADNE technique represents a novel, scalable, and universal method to obtain single-molecule mechanistic insights into key protein–DNA interactions in an environment containing physiologically-relevant nuclear proteins.

## INTRODUCTION

Watching DNA-binding proteins interact with DNA substrates in real-time at the single-molecule level illuminates how proteins detect and bind their targets at extraordinary detail. Key information about binding stoichiometry, order of assembly and disassembly, and how proteins diffuse to find their DNA targets are gained through single-molecule analysis (1–3). Various imaging techniques and optical platforms have been employed to resolve fluorescent proteins to the single-molecule level, but most of these techniques cluster into two broad categories – studies performed with purified proteins with defined conditions (1,4,5) or studies performed in living cells (6–8).

In single-molecule fluorescence studies of DNA-binding proteins, the molecules of interest must first be purified and then be labeled with a fluorescent tag, ranging in size from small chemical dyes to fluorescent proteins to large quantum dots (Qdots) (6). These techniques hold the distinct advantage of knowing precisely what proteins are binding to the DNA substrates of interest held in a static location. However, overexpressing, purifying, and labeling some proteins can prove difficult due to loss of activity. In addition, even using Qdot conjugation with antibodies, labeling is <100% (9). Furthermore, other protein factors that may contribute to stabilizing or destabilizing ligand binding and/or catalytic activity are lost during purification. The resulting studies of purified DNA-binding proteins may therefore not accurately represent how these proteins work in the context of the complex cellular milieu of the nucleus.

Conversely, single-molecule studies of DNA-binding proteins have also been performed within living cells (6–8). These techniques were developed for prokaryotes initially, but recent work has allowed for this imaging even in mammalian cells (10–15). While these approaches are the most biologically relevant, watching DNA-binding proteins sort through the complex genome to find their specific binding sites has proven challenging, but technically possible (16). However, these approaches rely on having low enough fluorescence signal to resolve individual proteins, and therefore there are often many unlabeled proteins of interest competing and altering binding lifetimes. Furthermore, protein diffusion along DNA cannot be studied when DNA strand orientation is unknown.

To overcome many of the challenges associated with the single-molecule characterization of DNA-binding proteins, we designed a new method that exists at the confluence of these two types of techniques, which we have termed SMADNE for single-molecule analysis of DNA-binding proteins from nuclear extracts. SMADNE applies similar principles of previous single-molecule work with cellular extracts (17–24) while making several significant modifications, allowing application to human cells and scalability to numerous proteins that bind DNA. Using the LUMICKS C-trap combined optical tweezers, microfluidics, and three-color confocal microscope, we precisely define the positions of fluorescently-tagged DNA repair proteins on 48.5 kb DNA substrates containing defined types of damage. As shown below, SMADNE provides binding specificity and diffusivity measurements, including characterizing multiple proteins simultaneously binding DNA damage with over four orders of magnitude of duration (0.1 to >100 s) and a wide range of 1D diffusivity values (from 0.001 to 1 μm^2^ s^−1^), with similar precision as other single-molecule techniques (4,25–33). At the same time, SMADNE bridges the complex milieu of the nuclear environment containing thousands of proteins to a system where fluorescently tagged single particles can be followed and characterized. Thus, SMADNE has broad applicability to provide detailed mechanistic information about diverse protein–DNA and protein-protein interactions.

## MATERIALS AND METHODS

### Expression and purification of recombinant UV-DDB

Recombinant full-length UV-DDB (DDB1-DDB2 heterodimer) was expressed in Sf9 cells coinfected with recombinant baculovirus of His_6_-DDB1 and DDB2-Flag, as performed previously (34). Briefly, a 5 ml His-Trap HP column pre-charged with Ni^2+^ (GE Healthcare) and anti-FLAG M2 affinity gel (Sigma) was used to purify DDB1-His_6_ and DDB2-Flag. The pooled anti-FLAG eluate containing UV-DDB (DDB1:DDB2 at a 1:1 ratio) was purified based on size with a HiLoad 16/60 Superdex 200 column (Amersham Pharmacia) in UV-DDB storage buffer (50 mM HEPES, pH 7.5, 200 mM KCl, 1 mM EDTA, 0.5 mM PMSF, 2 mM DTT, 10% glycerol and 0.02% sodium azide). Purified fractions of DDB1–DDB2 complex from the Superdex200 were aliquoted and flash-frozen with liquid nitrogen and stored at −80°C.

### Cell lines

U2OS cells were cultured in 5% oxygen in Dulbecco modified Eagle's medium (DMEM) supplemented with 4.5 g/l glucose, 10% fetal bovine serum (Gibco), 5% penicillin/streptavidin (Life Technologies). To obtain transient overexpression of the fluorescent-tagged proteins of interest, 4 ug of plasmid per 4 million cells was used to transfect using the lipofectamine 3000 reagent and protocol for 24 h (Thermo Fisher Cat# L3000008). For the experiments with cells stably expressing mNeonGreen-DDB2, U2OS cells were plated at 70% confluency and then transfected with the mNeonGreen-DDB2 overexpression plasmid. Forty-eight hours later, cells were expanded for five days in the presence of 500 μg/ml G418. After selection, cells were grown to confluency and sorted based on mNeonGreen fluorescence, and then utilized for single-molecule studies. Cells with overexpressed HaloTag fusions were treated with 100 nM (∼10–100-fold molar excess) of fluorescent HaloTag ligand for 30 min at 37°C (Janelia Fluor® 635 or 503 HaloTag® Ligand from Dr Luke Lavis Laboratory, Janelia Research Campus). To test if HaloTag labeling reactions were saturating the available HaloTag proteins, we also performed the labeling with 200 and 500 nM HaloTag ligand – the intensity of labeled protein did not significantly increase even at 500 nM, providing evidence that we were saturating the available binding sites (Supplementary Figure S1). In most cases, protein overexpression was performed one at a time, with the exception of the co-transfection of eGFP-DDB1 and HaloTag-DDB2 and a co-transfection of eGFP-XPC with unlabeled RAD23B. Protein overexpression was confirmed via western blot and by quantifying the fluorescence intensity in solution on the C-trap correlative optical tweezers and fluorescent microscope (Supplementary Figures S1 and S2, Supplementary Table S1). For the fluorescence intensity measurements, standard curves of the background photon counts apparent on the C-trap were created for purified GFP or purified HaloTag protein conjugated to the fluorescent dyes of interest, and the intensities of the HaloTag proteins adjusted for the amount of free dye present in the sample (Supplementary Figure S1E). The intensities of the nuclear extracts were then interpolated into the standard curves to determine concentration (Supplementary Table S1).

### Nuclear extraction

Nuclear extraction was performed the day after transient transfection using a nuclear extraction kit from Abcam (ab113474). After extraction following the protocol from the Abcam kit, the tubes were aliquoted into single-use aliquots and flash-frozen in liquid nitrogen prior to storage at –80°C. Upon use for single-molecule experiments, nuclear extracts were immediately diluted after thawing in buffer for experiments at a ratio of 1:10. See Supplementary Table S3 for a list of buffer conditions used in each experiment. Nucleic acid concentration was determined using a Quant-iT™ PicoGreen™ dsDNA Assay Kits (Invitrogen) and total protein concentration obtained using a Bradford assay (Bio-Rad) (dsDNA concentration was ∼2 ng/ul and total protein was on average 1.2 mg/ml). The reproducibility of overexpression and binding behavior of the nuclear extracts was confirmed by preparing multiple batches, and in the case of eGFP-DDB1 and HaloTag-DDB2 three batches of extracts were each tested over multiple days of collection with little variation in lifetimes from batch to batch.

### Western blots of overexpressed proteins from nuclear extracts

Various amounts of extracts and purified proteins (Supplementary Figure S2) were loaded onto 4–20% tris-glycine polyacrylamide gels (Invitrogen; XP04202BOX). Proteins were transferred onto a polyvinylidene difluoride membrane followed by blocking in 20% nonfat dry milk (diluted in PBST: phosphate-buffered saline containing 0.1% Tween 20) for 1 h at room temperature. Membranes were incubated with primary antibodies for 2 h at room temperature or overnight at 4°C, washed 3 × 10 min in PSBT, and incubated with peroxidase conjugated secondary antibodies for 1 h at room temperature. Membranes were washed again before developing using SuperSignal West Femto Maximum Sensitivity Substrate (Thermo Fisher Scientific; #34095). Primary antibodies used: PARP1 (1:100; abcam #ab227244), DDB2 (1:1000; abcam #ab181136), DDB1 (1:1000; Invitrogen #37-6200), XPC (1:1000; Novus #NB100-477) Polβ (1:1000; proteintech #18003-1-AP), OGG1 (1:1000; abcam #ab124741), and APE1 (1:100; Abcam #ab194). Secondary antibodies used: anti-rabbit IgG (1:50,000 Sigma #A0545), or anti-mouse IgG (1:50 000 Sigma #A4416). Blots were analyzed on ImageJ v1.53k. Overexpressed proteins were compared to purified proteins of interest, and in cases of XPC and DNA polymerase β (Polβ) the levels of endogenous protein from nuclear extracts without transfection were utilized (Supplementary Figure S2D).

### Mass spectrometry of nuclear extracts

A 2μg aliquot of nuclear extract (consisting of samples from eGFP-DDB1 and HaloTag-DDB2 overexpression and nontransfected control, each ran in triplicate) was analyzed by nano LC/MS/MS with a Waters M-class HPLC system interfaced to a ThermoFisher Fusion Lumos. Peptides were loaded on a trapping column and eluted over a 75 μm analytical column at 350 nl/min; both columns were packed with XSelect CSH C18 resin (Waters); the trapping column contained a 3.5 μm particle, the analytical column contained a 2.4 μm particle. The column was heated to 55°C using a column heater (Sonation). A 2 h gradient was employed. The mass spectrometer was operated in data-dependent mode, with MS and MS/MS performed in the Orbitrap at 60 000 FWHM resolution and 15 000 FWHM resolution, respectively. APD was turned on. The instrument was run with a 3 s cycle for MS and MS/MS. Data were processed through the MaxQuant software v1.6.2.3 (www.maxquant.org) which served several functions: (i) recalibration of MS data; (ii) filtering of database search results at the 1% protein and peptide false discovery rate (FDR); (iii) calculation of peak areas for detected peptides and proteins; (iv) data normalization using the LFQ algorithm. In total this analysis identified 669 nuclear-associated proteins, 167 proteins associated with the nucleus and mitochondria, 244 mitochondrial proteins, and 470 other proteins based on Gene Ontology Cellular Component classification, see Extended Data Table 1.

**Table 1.** Binding lifetimes for DNA repair proteins analyzed in this study

Protein	DNA substrate	Lifetimes (s) and percentages	Binding lifetime τ_avg_ (weighted average, s)	Lifetime(s) and percentages corrected for photobleaching	Corrected binding lifetime τ_c_ (weighted average, s)
YFP-PARP1	Nicked DNA	4.2 ± 0.2 s	4.2	5.2 ± 0.2 s	5.2
eGFP-XPC	UV-damaged DNA (40J)	0.9 ± 0.08 s (67 ± 4.9%)	16.8	0.9 ± 0.08 s (67 ± 4.9%)	75.5
		48.7 ± 26.3 s (33 ± 4.9%)		227 ± 27.1 s (33 ± 4.9%)	
tGFP-APE1	Nicked DNA	0.3 ± 0.02 s	0.30	0.3 ± 0.02 s	0.3
tGFP-Polβ	Nicked DNA	1.8 ± 0.03 s	1.8	2.0 ± 0.03 s	2.0
mNeonGreen-DDB2 [1]	UV-damaged DNA (40J)	1.7 ± 0.1 s (50 ± 0.9%)	7.6	1.7 ± 0.1 s (50 ± 0.9%)	9.2
		13.5 ± 0.5 s (50 ± 0.9%)		16.6 ± 0.5 s (50 ± 0.9%)	
mNeonGreen-DDB2 [2]	UV-damaged DNA (40J)	3.8 ± 0.2 s (24 ± 0.8%)	24	4.0 ± 0.2 s (24 ± 0.8%)	42.3
		31 ± 0.7 s (76 ± 0.8%)		54.4 ± 1.0 s (76 ± 0.8%)	
HaloTag-JF635-DDB2	UV-damaged DNA (40J)	3.9 ± 0.1 s (43 ± 1.35%)	29	4.0 ± 0.1 s (43 ± 1.35%)	39.0
		16.2 ± 1.1 s (33 ± 1.79%)		17.1 ± 1.1 s (33 ± 1.79%)	
		90.3 ± 6.0 s (24 ± 1.17%)		132.2 ± 6.3 s (24 ± 1.17%)	
eGFP-DDB1	UV-damaged DNA (40J)	1.8 ± 0.2 s (14 ± 1.2%)	29	1.8 ± 0.2 s (14 ± 1.2%)	43.7
		7.3 ± 0.2 s (44 ± 1.28%)		7.6 ± 0.2 s (44 ± 1.28%)	
		60.9 ± 1.1 s (42 ± 0.43%)		95.5 ± 2.0 s (42 ± 0.43%)	
HaloTag-JF635-DDB2	8-oxoG damaged DNA	0.14 ± 0.0013 s	0.14	0.14 ± 0.0013 s	0.14
eGFP-DDB1	8-oxoG damaged DNA	0.25 ± 0.002 s	0.25	0.25 ± 0.002 s	0.25
HaloTag-JF635-DDB2	UV-damaged DNA (40J) + 3 nM purified UV-DDB	1.1 ± 0.01 s	0.1.1	1.1 ± 0.01 s	1.1
eGFP-DDB1	UV-damaged DNA (40J) + 3 nM purified UV-DDB	0.6 ± 0.01 s	0.6	0.6 ± 0.01 s	0.6
mNeonGreen-DDB2 K244E	UV-damaged DNA (40J)	0.7 ± 0.06 s (53 ± 1.4%)	8.5	0.7 ± 0.06 s (53 ± 1.4%)	27.2
		16.9 ± 1.8 s (47 ± 1.4%)		57 ± 2.5 s (47 ± 1.4%)	
OGG1-mScarlet	8-oxoG damaged DNA	1.4 ± 0.01 s	1.4	1.5 ± 0.03 s	1.5
OGG1-eGFP	8-oxoG damaged DNA	0.7 ± 0.1 s (51 ± 3.9%)	2.0	0.7 ± 0.1 s (51 ± 3.9%)	2.0
		3.2 ± 0.3 s (49 ± 3.9%)		3.3 ± 0.3 s (49 ± 3.9%)	
OGG1(K249Q)-eGFP	8-oxoG damaged DNA	7.7 ± 0.27 s (78 ± 2.29%)	15.4	8.9 ± 0.37 s (78 ± 2.29%)	47.2
		42.9 ± 8.7 s (22 ± 2.29%)		183 ± 11.6 s (22 ± 2.29%)	

1: Stably expressed mNeonGreen-DDB2.

2: Transient transfected mNeonGreen-DDB2.

### DNA substrate generation

Lambda DNA for C-trap experiments was purchased from New England Biotechnologies. The ends were biotinylated by adding a mix of 6 μg lambda DNA, 50 μM nucleotide mix (with dATP, dGTP, dTTP, and biotinylated dCTP), 15 units of Klenow fragment polymerase (NEB) and 1x concentration of NEB Buf 2. By filling in the overhangs on the cos sites of lambda DNA, this reaction labels one side of the lambda DNA with four biotins and the other with six. The reaction was incubated for 30 min at 37°C and then the free nucleotides were removed from solution via ethanol precipitation, with 1 μg/μl glycogen used as a co-precipitant to increase the yield. Biotinylation of the lambda DNA was confirmed by generating force–distance curves on the C-trap instrument and fractions were frozen down in aliquots of 20 ng/μl at –20°C. After thawing aliquots, they were stored at 4°C for up to 2 weeks and then discarded.

Biotinylated lambda DNA was then utilized to generate various forms of DNA damage for SMADNE characterization. To create UV-damage, biotinylated lambda DNA was irradiated with UV-C for 40 J/m^2^. Similarly, to create oxidative damage on lambda DNA, a single use aliquot was incubated with 0.2 μg/ml methylene blue (as performed here (35)) and exposed to 660 nm light for 10 min. Lastly, DNA with single-stranded breaks (nicked DNA) was generated by digesting 1 ug of DNA with the nickase Nt.BspQI (NEB) following the manufacturer's instructions. This nickase recognizes 10 distinct sequences of 5′-GCTCTTCN-3′ along the lambda DNA to generate 10 nicks, cutting on the 3′ side of its recognition sequence (Supplementary Figure S3A). Only eight out of ten nicks are observable by fluorescence because two sites are within 436 bp and the other is too close to the bead to be discerned. After nicking the DNA, fluorescent nucleotides were incorporated at the sites using nick translation for identification of nick sites, using a 40 μM mix of dGTP, dCTP, dATP and fluorescein-tagged dUTP, as well as 10 units of pol I and 800 ng nicked lambda DNA. Results for this nick translation reaction agreed with the anticipated sites of DNA nicks with few off-target incorporations (Supplementary Figure S3B and C).

### Single-molecule experiments

#### DNA tether formation and positioning

**Figure 1.**
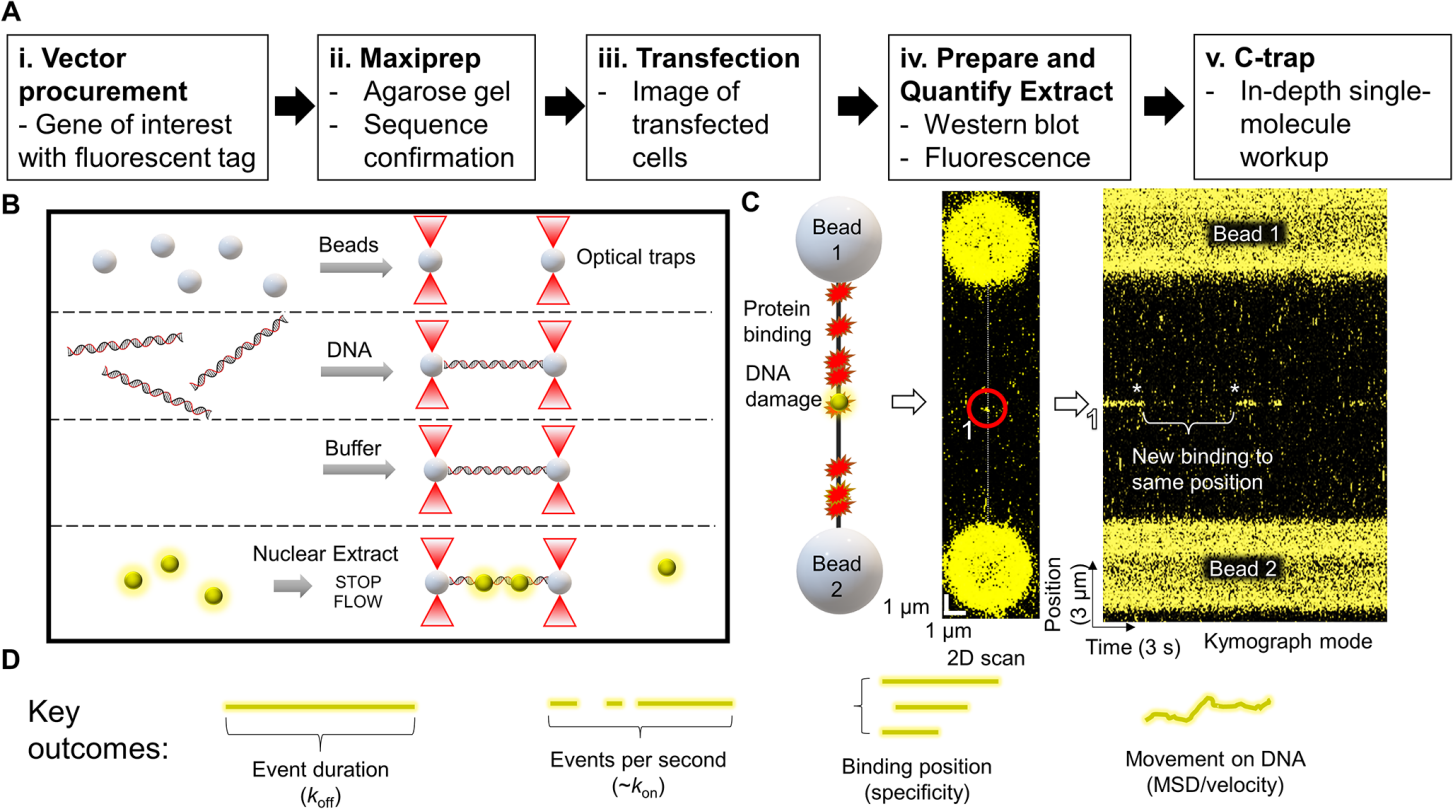
The workflow and experimental outcomes of SMADNE. (**A**) SMADNE workflow. (**B**) A diagram of the imaging techniques using four channels separated by laminar flow. (**C**) A cartoon of a DNA substrate for SMADNE suspended between two streptavidin-coated polystyrene beads and fluorescently-tagged proteins (yellow sphere) binding to sites of DNA damage. This substrate (nicked DNA) is also shown as a 2D scan (one YFP-PARP1 binding event numbered and circled) and in kymograph mode (numbered spot marked). Event one dissociated before the kymograph started and then another event appeared at the same position later (asterisks). Binding events appear as lines in the kymograph because time is indicated on the X-axis and position on the Y-axis. (**D**) The four major outcomes obtained from SMADNE characterization. See also Supplementary Figures S1 and S2.

Single-molecule experiments were performed on a LUMICKS C-Trap instrument, which consists of a three-color confocal fluorescence microscope and dual-trap optical tweezers (36). A microfluidic flow-cell from LUMICKS was used containing five distinct flow channels separated by laminar flow that could be traversed by the two optical traps. However, only four of the flow channels were utilized for these experiments (Figure 1). To prepare the DNA substrates for single-molecule imaging, channels one, two and three were filled with 4.38 μm streptavidin-coated polystyrene beads (LUMICKS), biotinylated DNA, and buffer of interest, respectively. All three were flowed at a pressure of 0.3 bar to maintain laminar flow. While maintaining flow, single beads were caught in both optical traps in channel one. Then, the beads were moved to channel 2 for DNA capture. To suspend DNA between the two traps, the bead in trap 2 was held in a constant position while moving trap 1 downstream and upstream of the flow (keeping the two traps parallel in the flow but varying the distance). By measuring force-distance curve each time the traps were spread apart, an increase in the force with an increased distance indicated the binding of a DNA tether. The force-distance curves were then compared to the extensible wormlike chain model for DNA of 48 500 bp to verify that a single tether of dsDNA was caught (37).

After tether formation, the beads with the suspended DNA were moved to the buffer channel (channel three) and channel three and four were flowed at 0.3 bar for at least 10 s to introduce nuclear extracts into the flow cell. After flushing in the extract, the flow was then stopped and the traps were moved to the position where channel four (the channel with nuclear extracts) joins the flow cell. Immediately after that (unless otherwise indicated), force-distance curve was re-zeroed at that position and bead one was pulled to generate the tension desired for data collection (typically 10 pN). Importantly, over the course of data collection, we found that nuclear debris from the extract would also get trapped in the optical traps, thus changing the apparent force measurement by positive or negative 6 pN over 5 min of collection. Therefore, after determining the initial force curve and defining the positions of the traps required to maintain the desired force, the trap positions were not altered throughout the data collection to maintain a constant force on the DNA throughout the data collection.

### Confocal imaging

Various fluorophores were utilized throughout this study, and each was excited with the laser closest to their maximum excitation wavelength. eGFP, tGFP, YFP, fluorescein, mNeonGreen and HaloTag-JF-503 were excited with a 488 nm laser and emission collected in a 500–550 nm band pass filter, mScarlet was excited at 561 nm and emission collected in a 575–625 nm band pass filter, and HaloTag-JF-635 was excited with a 638 nm laser and emission collected in a 650–750 nm band pass filter (see Supplementary Table S2). All data were collected with a 1.2 NA 60× water emersion objective and photons measured with single-photon avalanche photodiode detectors. With each fluorophore, the imaging settings were set with both the photostability and binding lifetimes in mind (Supplementary Tables S2, S3). Typically, each laser was set to 5% power and scanned continuously (0.1 msec of exposure for each pixel of size 100 nm. For λ DNA these settings result in ∼30 frames per second). However, for some binding events with long binding lifetimes and lower photostability (i.e. eGFP-tagged DDB1), a pulsed excitation was utilized. In this imaging scheme, the same exposure time and laser power was utilized, but brief pauses were included between each exposure. In the case of eGFP-DDB1, for instance, data was collected with a 34 ms exposure followed by 66 ms pause in exposure, thus increasing the fluorophore lifetime by threefold. See Supplementary Table S3 for a table of laser powers, average binding lifetime, photobleaching lifetime with each fluorophore, and exposure settings. All data presented were obtained from multiple collection days and for most proteins at least two differently preparations of nuclear extracts.

### Single-molecule Förster resonance energy transfer imaging

For the FRET approach in Figure 5, data were collected at 50% power of the 488 nm laser at 34 ms per frame to excite the FRET donor eGFP-DDB1, and the intensity of DDB2-mCherry was measured as the FRET acceptor. For quantification of the signal, lines that exhibited acceptor emission were tracked with Pylake, and then downsampled by a factor of ten to increase the signal-noise of the fluorescence data. To subtract for background signal in the quantifications of the intensities, photon counts for each channel were taken for the region between 6 and 9 pixels on either side of the tracked line (resulting in zones that follow the path of the event in regions without fluorescent signal. We then subtracted any bleedover from the eGFP-DDB1 by collecting multiple events with both colors, photobleaching the mCherry-DDB2 signal, and then measuring the resultant intensities in the acceptor channel caused by eGFP emission. These intensities were consistently 9.0% of the intensity of eGFP in the FRET donor emission channel, so that ratio was used for subtracting the bleedover.

### TIRF C-trap experiments

Other single-molecule fluorescence experiments were performed on a commercial optical tweezers and microfluidics system using the TIRF objective (C-trap; LUMICKS). This system is equipped with five microfluidic channels, four were used as follows: channel 1 contained 3.7 μm diameter streptavidin-coated polystyrene beads (Spherotech), channel two contained biotinylated λ-DNA (damaged beforehand with 40 J/m^2^ UVC), channel three contained buffer and channel four contained nuclear extract with overexpressed eGFP-DDB1 and HaloTag-DDB2 conjugated to Janelia fluor 635.

Following bead capture in channel one the tethered DNA was held 10 μm above the surface in channel two using the laser tweezers at 30% power. Flow at 0.2 ± 0.05 bar was used during DNA capture and a single strand of damaged biotinylated λ-DNA was tethered between the beads. The DNA tensions used were 10 pN for experiments without flow and 30 pN with flow. The tether was then transferred to the nuclear extract in channel four. Depending on the experiment, the flow was kept constant at 0.05 ± 0.03 bar; pulsed at 0.05 ± 0.03 bar for 3 s on then 10 s off; or the channel was flushed for ∼10 s at 0.1 ± 0.05 bar to introduce fresh protein and binding was observed without flow. Fluorophores were excited with the 488 nm (80% power) and 638 nm (40% power) lasers for 200 ms with exposure synchronization. Videos were taken over the region encompassing the tether and beads at a framerate of 4.3 Hz.

### Data analysis

Images and force data for kymographs collected were exported and analyzed using custom software by LUMICKS (Pylake). For visualization of the kymographs and 2D scans after exporting, the utility C-Trap .h5 Visualization GUI (2020) by John Watters was used as downloaded from harbor.lumicks.com. As data were collected with images containing both the DNA of interest and the streptavidin-coated polystyrene beads, the pixels on the edge of the beads were first defined to determine the start and the end positions of the DNA. Line tracking was performed using a custom script from LUMICKS based on performing a Gaussian fit over the line intensity and connecting the time points to form a line using previous line tracking algorithms (38). Of note, fluorophores derived from GFP tended to blink for periods up to two seconds, which caused line tracking programs to identify a single event as two separate binding events. To address this issue, the tracked lines were curated to determine if any events occurred at the same position (<100 nm) with off times less than 2 s—the gaps in these lines were manually connected using a feature of the LUMICKS software. After tracking the lines, the position and time data for each line was used to determine each line's duration, the number of lines per minute, and the average position of each line.

For motile events, mean squared displacement (MSD) was calculated using a custom script provided by LUMICKS, with this equation:


$$\begin{equation*}MSD\ \left( {n\Delta t} \right) = \frac{1}{{N - n}}\ \mathop \sum \limits_{i\ = \ 1}^{N - n} {\left( {{x_{i + n}} - {x_i}} \right)^2}\end{equation*}$$

where *N* is total number of frames in the phase, *n* is the number of frames at a given time step, *Δt* is the time increment of one frame, and *x_i_* is the particle position in the *i*th frame. The diffusion coefficient (*D*) was determined by fitting a model of one-dimensional diffusion to the linear portion of the MSD plots:


$$\begin{equation*}MSD\ \left( {n\Delta t} \right) = \ 2D{\left( {n\Delta t} \right)^\alpha } + y\end{equation*}$$

where α is the anomalous diffusion coefficient and *y* is a constant (*y*-intercept). In order to ensure the best fit possible, the table of time steps and MSD values was exported and fit using GraphPad Prism. The fit was manually adjusted to include as much of the linear portion of the graph as possible. Fittings resulting in *R^2^* less than 0.8 or using <10% of the MSD plot were excluded. Furthermore, for lines less than 1 s long the anomalous diffusion coefficient was fixed to 1 (i.e. a linear fit of diffusivity was utilized).

### TIRF C-trap data analysis

Videos were analyzed using ImageJ (https://imagej.nih.gov/ij/). In the case of DDB1 and DDB2 images two channels were overlaid and aligned using Align RGB planes plugin (https://blog.bham.ac.uk/intellimic/g-landini-software/), using the laser tweezer captured beads as fiducial markers. Line traces along the position of the DNA tether were converted to kymographs, which provided continuous streaks corresponding to bound molecules. Lifetimes were determined by measuring the length of the streaks and converted to time, based on the known framerate. Bound lifetimes were analyzed using the cumulative residence time distribution (CRTD) approach (39). CRTDs were then fitted to single (DDB1, DDB1 and DDB2) or double (DDB2) exponentials based on fit quality and examination of residuals. Fitting was performed in Microsoft Excel using Solver. Fit errors are SEM. As the photobleaching rates were similar to the rates of dissociation in this data, corrections to the lifetimes were made as previously published (40).

### Colocalization analysis

For colocalization analysis, lines tracked from the data (after selecting the region of the scan containing the DNA not including the beads) were compared against each other using a custom-made colocalization analysis script. Briefly, times and positions for each datapoint of each line were compared between the two sets of lines to determine if the distance and time agreed within an adjustable window (less than 200 nm and 400 ms apart). By calculating the data this way, even events that started without colocalization before diffusing to a colocalized position would be counted—however no datasets with motile events were used for colocalization analysis. After determining colocalization, events are sorted into 11 categories as previously established by other single-molecule studies (41). This script, named colocalization analyzer, is available at harbor.lumicks.com/scripts.

### Photobleaching analysis

Photobleaching decay constants were determined for each fluorophore by collecting kymographs with continuous exposure of immobilized fluorescent proteins non-specifically adsorbed to the bottom of the flow chamber. Because the microenvironment of these fluorophores is different than the ones bound to DNA in the center of the flow cell, these measured photobleaching rates may not be exactly the same as the fluorophores used during experiments. However, performing the photobleaching measurement this way allows for these measurements to rapidly be performed for each fluorescent tag in the same conditions utilized during experiments. To collect these kymographs, we lowered the objective of the C-trap to focus on to the bottom of the flow chamber until defined single-molecule spots could be observed and photon counts per second reached a maximum. After focusing, a minimum of three kymographs were imaged using the collection settings of interest. Photon counts from the appropriate channel were binned into bins consisting of 1 s intervals and the resulting bins fit to a single-exponential decay function to determine photobleaching lifetimes (Supplementary Table S2). This script, named photostability calculator, is also publicly available at harbor.lumicks.com/scripts. All binding lifetimes reported in the text have been corrected for the photobleaching rate of each respective fluorophore (τ_c_) (40). Both raw values (τ_avg_) and corrected values are available for each experiment in the appropriate figures, Table 1 and Supplementary Table S3.

## RESULTS

### SMADNE workflow and characterization of PARP1 binding to damaged DNA

To study fluorescently tagged DNA-binding proteins from nuclear extracts, we developed the workflow shown in Figure 1A, B (and Materials and Methods). Western blotting and fluorescence intensity of the tagged protein were utilized to provide estimates of the target protein in the extract (Supplementary Figures S1 and S2, Supplementary Table S1), which are generally 50–100 times more prevalent than the endogenous protein under study. Thus, endogenous proteins are probably too dilute to affect overall binding of the transiently expressed fluorescently labeled-proteins (Supplementary Figure S2) (42). Mass spectrometry confirmed that our nuclear extraction protocol enriches for nuclear proteins, with 836/1550 proteins identified associated with the nucleus (Extended Data Table 1). Using the LUMICKS C-trap optical traps, streptavidin-coated polystyrene beads were captured and biotinylated 48.5 kb DNA was suspended between the beads (Figure 1C, left panel). After flowing in the nuclear extract containing the fluorescently labeled protein of interest, flow was stopped, and 2D confocal images were collected to verify binding of the protein to the DNA (Figure 1C, middle panel). Then, the area being scanned was reduced to only the central DNA position. In this 1-dimensional (1D) scanning mode, imaging rates as fast as six msec per scan can be achieved (166 frames per second). These data appear as fluorescent time streaks (kymographs) showing the fluorescently-tagged protein position over time, where the Y-axis represents the position on the DNA and the X-axis shows the scan time (Figure 1C, right panel). In this mode, the Y-axis represents positions on the DNA where binding occurs, and the X-axis shows the scan time, which in this kymograph is 33 ms increments for 30 frames per second.

To validate the general utility of SMADNE, we examined a series of fluorescently-tagged DNA repair proteins on various DNA substrates, namely poly(ADP-ribose) polymerase 1 (PARP1), xeroderma pigmentosum complementation group C protein (XPC), apurinic/apyrimidinic endonuclease 1 (APE1), DNA polymerase β (Polβ), DNA damage-binding protein 1 (DDB1) and DNA damage-binding protein 2 (DDB2). In Figure 1, YFP-PARP1 forms transient complexes on nicked DNA, creating time streaks in the kymograph mode. Notice how multiple molecules revisit the same positions on the DNA (Figure 1C, asterisks). These likely represent multiple events on the same damage site. Using SMADNE, we determined four key outcomes: (i) how long a binding event lasts from start to finish (*k*_off_); (ii) how many binding events per second occur (related to *k*_on_); (iii) the position of binding events along the DNA; and (iv) how bound proteins diffuse along the DNA (Figure 1D).

### SMADNE characterization of PARP1 binding nicked DNA: increasing DNA tension increased binding events

**Figure 2.**
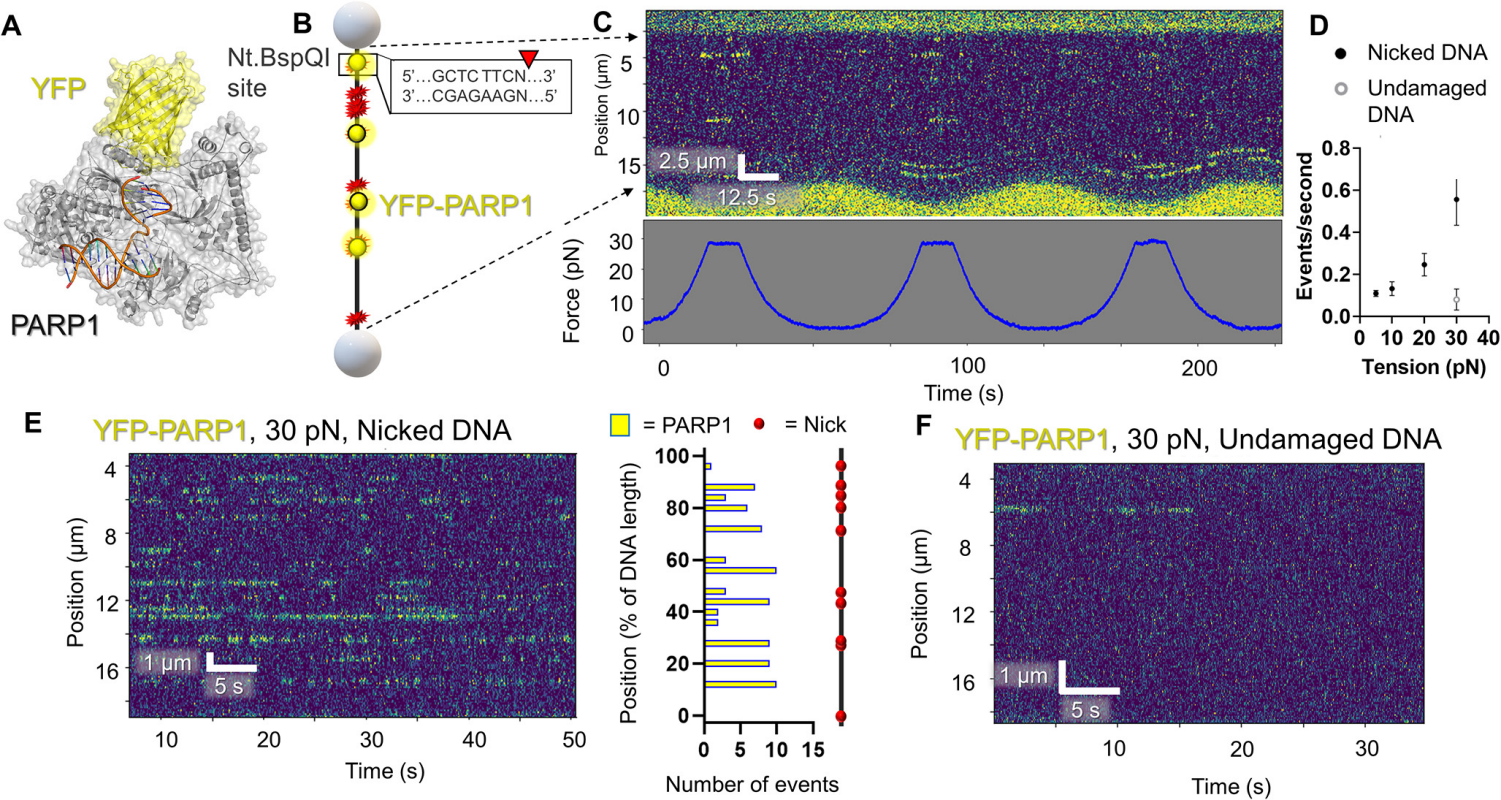
DNA tension influences DNA nick detection by PARP1. (**A**) A structural model of PARP1 bound to nicked DNA with YFP tag (PDB codes 3ED8 and 4KLO) generated as in this reference (1). (**B**) A schematic of the DNA suspended between streptavidin-coated polystyrene beads containing ten discrete nicks from the nickase Nt.BspQI. (**C**) An example kymograph of PARP1 binding DNA at oscillating tensions from 5 to 30 pN. Binding events shown in yellow and tension measurements shown below in blue. (**D**) Number of events per second at various DNA tensions held constant. Error bars represent the SEM of three experiments. Gray circle represents undamaged DNA. (**E**) An example kymograph of PARP1 binding DNA at constant tension (30 pN). Positional analysis shown to the right showed binding at the expected sites, but also several sites that were bound multiple times that did not contain the recognition sequence by Nt.BspQI. (**F**) Undamaged DNA exhibited reduced YFP-PARP1 binding, even at 30 pN. See Supplementary Figures S1–S4.

To demonstrate the broad applicability of SMADNE to various DNA repair proteins and different forms of DNA damage, the binding interactions were examined for YFP-tagged PARP1 from nuclear extracts on DNA containing ten nicks generated by a sequence-specific nickase (Figure 2A and B). For YFP-PARP1 at 10 pN of DNA tension, the average lifetime corrected for photobleaching (τ_c_) was 5.2 s, events occurred at 0.13 events per second, the positions agreed with the expected sites, and no diffusion along the DNA was observed (Figure 2, Supplementary Figure S3, Table 1 and Supplementary Table S3). Unexpectedly, increasing the tension on the DNA from 5 to 30 pN dramatically increased the number of YFP-PARP1 events per second. At 30 pN, new binding sites also appeared that were not observed at lower tension (Figure 2C). It is possible that the higher tension makes previously existing nicks more identifiable by PARP1. Datasets were collected at various constant DNA tensions. While binding lifetimes stayed relatively consistent as analyzed by fitting a cumulative residence time distribution (CRTD) to an exponential decay function (Supplementary Figure S3), events per second increased four-fold at 30 pN of tension. In contrast, undamaged events per second remained low even at high tensions (Figure 2D). YFP-PARP1 from nuclear extracts repeatedly bound at specific locations on the DNA, both on undamaged and damaged DNA (Figure 2E, F). The latter substrate indicates repeated specific binding events occurred at the nick sites. Datasets collected at 30 pN tension resulted in numerous binding events at 13 positions on the nicked DNA, indicating some off-target DNA damage present in our DNA sequence (Figure 2E). While no previous work to our knowledge has examined PARP1 binding to nicked DNA at the single-molecule level, we and another recent publication examined single molecules of purified PARP1 on abasic sites and gapped DNA, finding that PARP1 largely bound its substrate via 3D diffusion, which agrees with the results we observed with nicked DNA using SMADNE (4,43).

### Application of SMADNE to study transient DNA interactions

**Figure 3.**
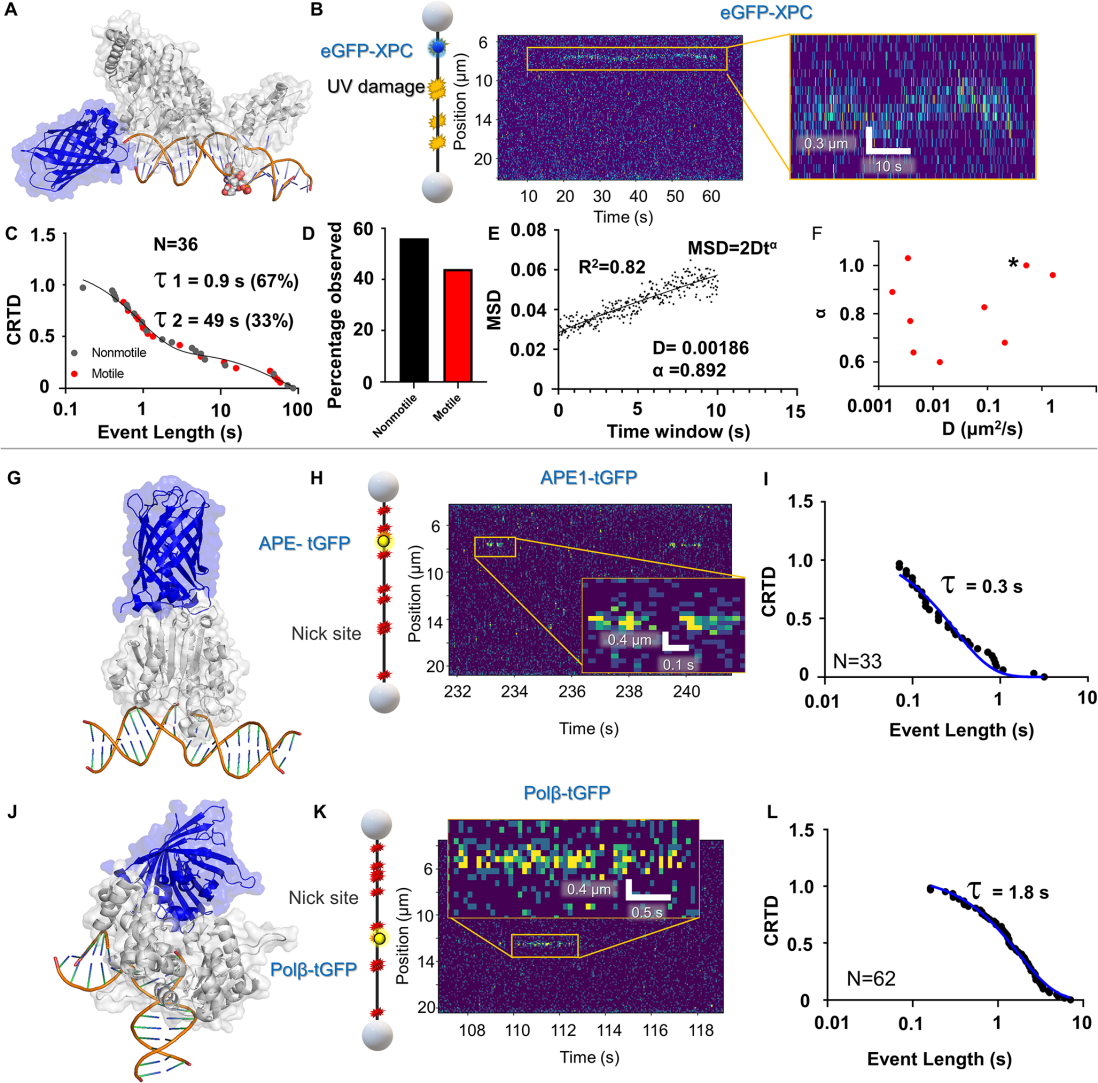
DNA-binding interactions of other DNA repair proteins. (**A**) The structure of eGFP-XPC (PDB codes 6CFI of Rad4 the yeast homolog to XPC and 4EUL). (**B**) A cartoon depiction of the DNA substrate used for XPC binding characterization, with UV damage sites shown in yellow and XPC binding shown in blue. Also shown is an example kymograph of eGFP-XPC binding and diffusing along the DNA in yellow. (**C**) CRTD analysis of XPC binding DNA with UV damage. (**D**) Distribution of motile and nonmotile XPC events. (**E**) An example MSD plot for analyzing XPC diffusion on DNA (D, in μm^2^/s). (**F**) Diffusion and α values for the diffusion of XPC on DNA. Event marked with asterisk was too short to determine an α value so it was defined as 1.0. (**G**) A structural model of APE1-tGFP from PDB code (5WNO and 4EUL). (**H**) Schematic and example kymograph of APE1 binding to DNA with nicks. (**I**) CRTD analysis of APE1 binding nicked DNA, with fit shown in blue. (**J**) A structural model of polβ-tGFP, taken from PDB codes (4KLO and 4EUL) and the tGFP modeled in. (**K**) Example schematics of polβ binding DNA containing nicks as well as a corresponding kymograph of an observation of polβ binding. (**L**) CRTD analysis of polβ binding nicked DNA, with the fit shown in blue. See Supplementary Figure S4.

We next sought to push the limits of the SMADNE technique to more transient interactions, such as XPC-RAD23B that diffuses along the DNA while detecting UV damage, as well as APE1 or Polβ binding to nicks with much lower affinity (Figure 3, Supplementary Table S3) (44–46). For XPC-RAD23B, eGFP-tagged XPC and nonfluorescent RAD23B were co-transfected, and eGFP signal was observed on UV-damaged (40 J/m^2^) DNA (Figure 3A). Thus, XPC binds UV-damaged DNA and, in 44% of events, diffused along the DNA (Figure 3B, D). Binding lifetimes for XPC in nuclear extracts were similar to those observed for purified XPC (45), with the CRTD fitting to a double exponential to yield one lifetime at 48.6 s and a second lifetime at 0.89 s, with the fast component contributing 67%. With photobleaching correction this yields a τ_c_ value of 75.5 s (Figure 3C, Table 1 and Supplementary Table S3). Mean squared dissociation (MSD) analysis performed on the motile XPC molecules (Figure 3E) revealed a diffusion constant with a geometric mean of ∼0.03 μm^2^ s^−1^, which agrees with previously published work (45) (Figure 3F). Additionally, tGFP-tagged APE1 and Polβ binding were also characterized on DNA with ten nicks as previously done with PARP1. Both proteins bound the nicked substrate with relatively lower affinity, with APE1 exhibiting a τ_c_ of 0.3 s (Figure 3G-I) and Polβ with a τ_c_ of 2.0 s (Figure 3J-L, Table 1). No binding for these three proteins was observed for undamaged DNA (Supplementary Figure S4).

### Using SMADNE to observe protein dynamics on DNA: a case study with UV-DDB

**Figure 4.**
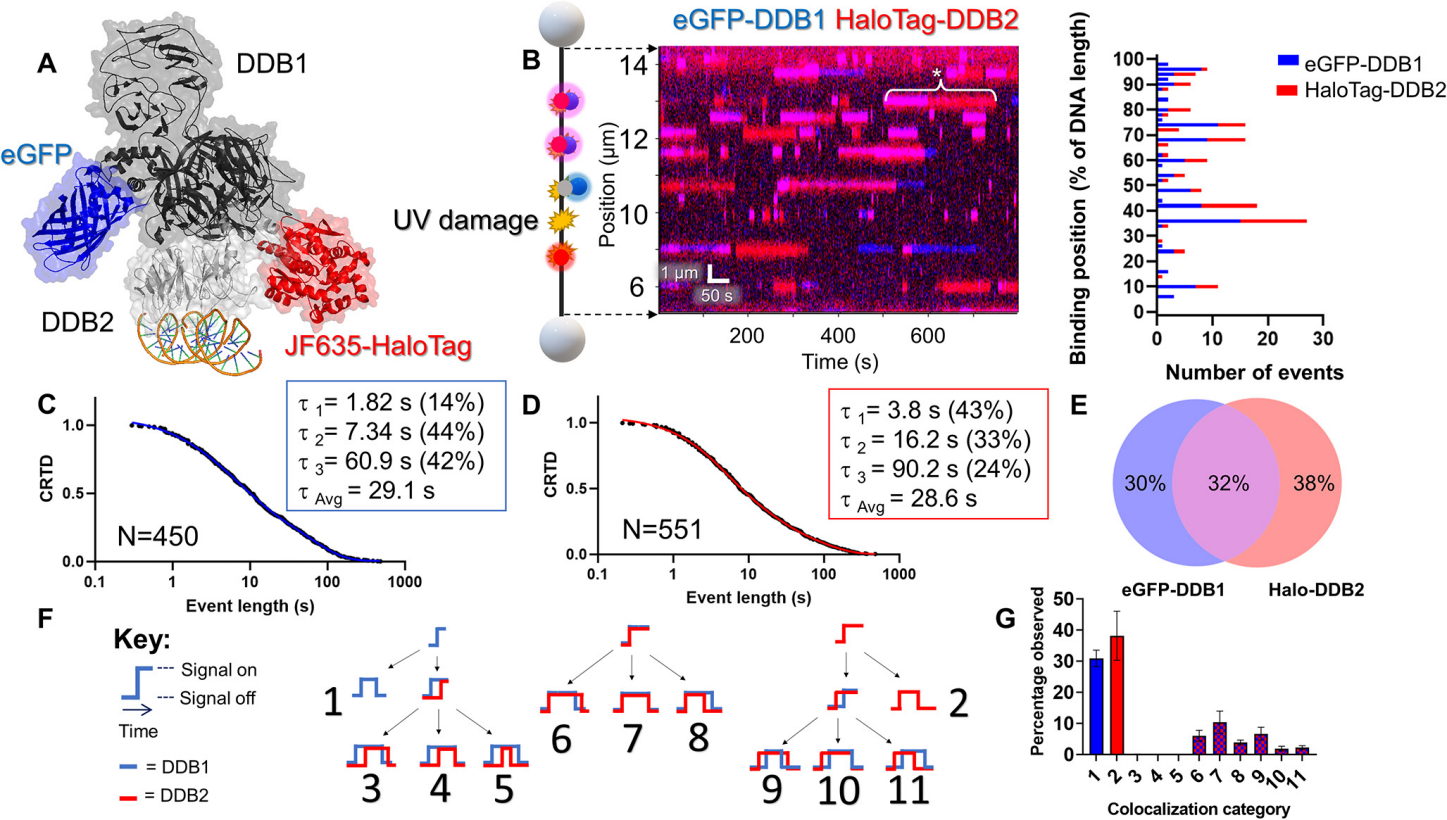
SMADNE characterization of dual-labeled UV-DDB binding UV damage. (**A**) Structure of UV-DDB bound to DNA (PDB ID: 4E5Z, 4EUL, 5UY1) with modeled fluorescent tags. (**B**) An example kymograph of eGFP-DDB1 (blue) and HaloTag-DDB2 (red) binding to 48.5 kb DNA with UV damage. When both colors bind together the color appears magenta. The white asterisk marks an event where DDB1 and DDB2 bound together followed by DDB1 dissociation. Also shown is a graph of the positions of events in the kymograph. (**C**, **D**) Cumulative residence time distribution (CRTD) for DDB1 (C) and DDB2 (D) binding UV-damaged DNA. (**E**) Percentage of events that were DDB1 alone, DDB2 alone, or colocalized (middle). (**F**) A diagram showing the 11 possible colocalization categories for two colors of molecules binding DNA. **G**, The distribution of the 11 categories for DDB1 and DDB2 binding UV-damaged DNA. Error bars represent the SEM of four experiments. See Supplementary Figures S4–S6.

We next studied the DNA repair protein UV-DDB, which is composed of a heterodimer between DNA damage-binding protein 1 (DDB1, 127 kDa) and DNA damage-binding protein 2 (DDB2, 48 kDa). This latter subunit engages DNA at the site of damage (5). UV-DDB detects UV-induced photoproducts with high affinity (47), and the purified protein has been extensively characterized at the single-molecule level for various DNA substrates (5,29,34). Thus, these previous studies serve as a benchmark in which to validate the behavior of UV-DDB by SMADNE. Since UV-DDB is a heterodimer, it provided an opportunity to orthogonally label DDB1 with an N-terminal eGFP tag and DDB2 with an N-terminal HaloTag conjugated to JaneliaFluor 635 dye under saturating conditions of 100 nM (Supplementary Figure S1, Materials and Methods, and Figure 4A) (48). After co-transfecting both plasmids into U2OS cells and obtaining a nuclear extract, we found that the concentration of the overexpressed protein in the flow cell was ∼0.1–0.4 nM, which was 50–100-fold higher than that of the endogenous proteins by western blot (Supplementary Table S1).

As reported by previous studies of purified protein in these buffer conditions, UV-DDB did not exhibit 1D diffusion (sliding) on the DNA but rather found its damaged substrates via 3D diffusion (5). Furthermore, DDB1 and DDB2 bound specific positions on the DNA multiple times within a single viewing window (Figure 4B). These long-lived binding positions (lifetimes > 10 s) represent the sites of UV photoproducts after UV treatment (40 J/m^2^). Non-damaged DNA supported significantly fewer and shorter binding events with short dwell-times (<10 s) (Supplementary Figure S4). With increasing UV dose, the number of binding events increase with emergence of long-lived UV-DDB complexes (Supplementary Figure S4 E–H). Within these damage sites, some positions had many short interactions over the course of a kymograph (consistent with a low-affinity substrate being weakly bound and released multiple times) and some positions only had a few long interactions (consistent with a high-affinity substrate strongly bound by UV-DDB). This pattern may indicate binding to cyclobutane pyrimidine dimers and 6–4 photoproducts, respectively, both of which are products of UV irradiation (49).

The binding events of both DDB1 and DDB2 exhibited a wide distribution of binding durations (four orders of magnitude) in good agreement with previous studies of purified Qdot-labeled UV-DDB (Figure 4C, D). Binding event durations were fit to CRTD to quantify the rate of dissociation (*k*_off_) (5). The DDB1 and DDB2 plots were fit to a triple-exponential decay function as was previously reported for purified UV-DDB, with one short lifetime (∼2 and 4 s respectively) one medium lifetime (7 and 16 s respectively), and one long lifetime (61 and 90 s respectively) in good agreement with previous studies using purified proteins (Figure 4C, D, Table 1, Supplementary Table S3) (5). After correcting for photobleaching, the weighted average lifetimes yield a τ_c_ value for DDB1 was 43.7 s, relatively close to DDB2 at 39.0 s. Incorporating the data from both the dissociation (*k*_off_) and association (*k*_on_) rates of DDB2 yields an equilibrium dissociation constant (*K_d_*) ∼0.4 nM, which agrees well other studies analyzing the affinity of purified UV-DDB to various UV-photoproducts, (Supplementary Figure S7) (50–52). To test the impact of fluorescent tags on lifetimes, we also collected a dataset with overexpressed mNeonGreen-DDB2, which exhibited a similar τ_c_ of 42.3 s (Supplementary Figure S7). These weighted average lifetimes were around two-fold longer than the previous single-molecule observations with purified UV-DDB on UV-damaged DNA (weighted average of 18.5 s) (5). As the previous strategy relied on Qdot-conjugated UV-DDB, this shorter lifetime observed previously could be due to Qdot conjugation process causing a modest reduction in UV-DDB binding affinity and thus a decreased lifetime as compared to our new fusion protein approach. Alternatively, unlabeled interacting proteins in the nuclear extract, such as heat shock proteins (Extended data table 1), could help stabilize UV-DDB (53). These two-color results were also validated using a C-Trap instrument with total internal reflection fluorescence capabilities, and similar trends of colocalization and binding lifetimes were observed (Supplementary Figure S5).

Using the dual-labeling approach, the frequency of DDB1 and DDB2 co-localization within the localization precision of our instrument (∼150 bp with these fluorophores, see Supplementary Figure S6) was quantified. Consistent with UV-DDB acting as a stable heterodimer, we saw many events colocalize—32% of events had at least one colocalization with the second color, compared to 30% of events that were either one molecule of eGFP-DDB1 or 38% that were HaloTag-DDB2 (Figure 4E). Although we observe colocalized events and single-color DDB1 events, the events we collect could also represent more complex scenarios, like DDB1 bound to an unlabeled or photobleached DDB2, or other proteins in the nucleus bringing DDB1 to the sites of damage. To ensure that the colocalized binding events were from one heterodimer of UV-DDB rather than a dimer of heterodimers or two heterodimers bound closely together (5), we also studied a mix of two colors of HaloTag-DDB2 (JF503 and JF-635) which rarely colocalized (∼2%, see Supplementary Figure S6).

SMADNE also allows the dynamics of multiprotein interactions on DNA to be analyzed. Including single-color events (without colocalization), there are 11 possible event classes of molecular interactions on DNA (Figure 4F), nine of which are colocalization events with unique assembly and disassembly mechanisms. We wrote a script to classify these 11 different types of events (publicly available on LUMICKS Harbor as “Colocalization Analyzer”) and found that the most common type for our data was category 7, in which DDB1 and DDB2 arrive and dissociate together. These data are consistent with UV-DDB acting as a stable heterodimer. However, we also saw the next most common was category 9, where DDB2 binds first followed by DDB1 and then DDB1 dissociates before DDB2, suggesting that alternative modes of binding exist where the proteins sequentially assemble and disassemble from the damage. Of note, categories 3–5 appeared exceedingly rare (Figure 4G).

**Figure 5.**
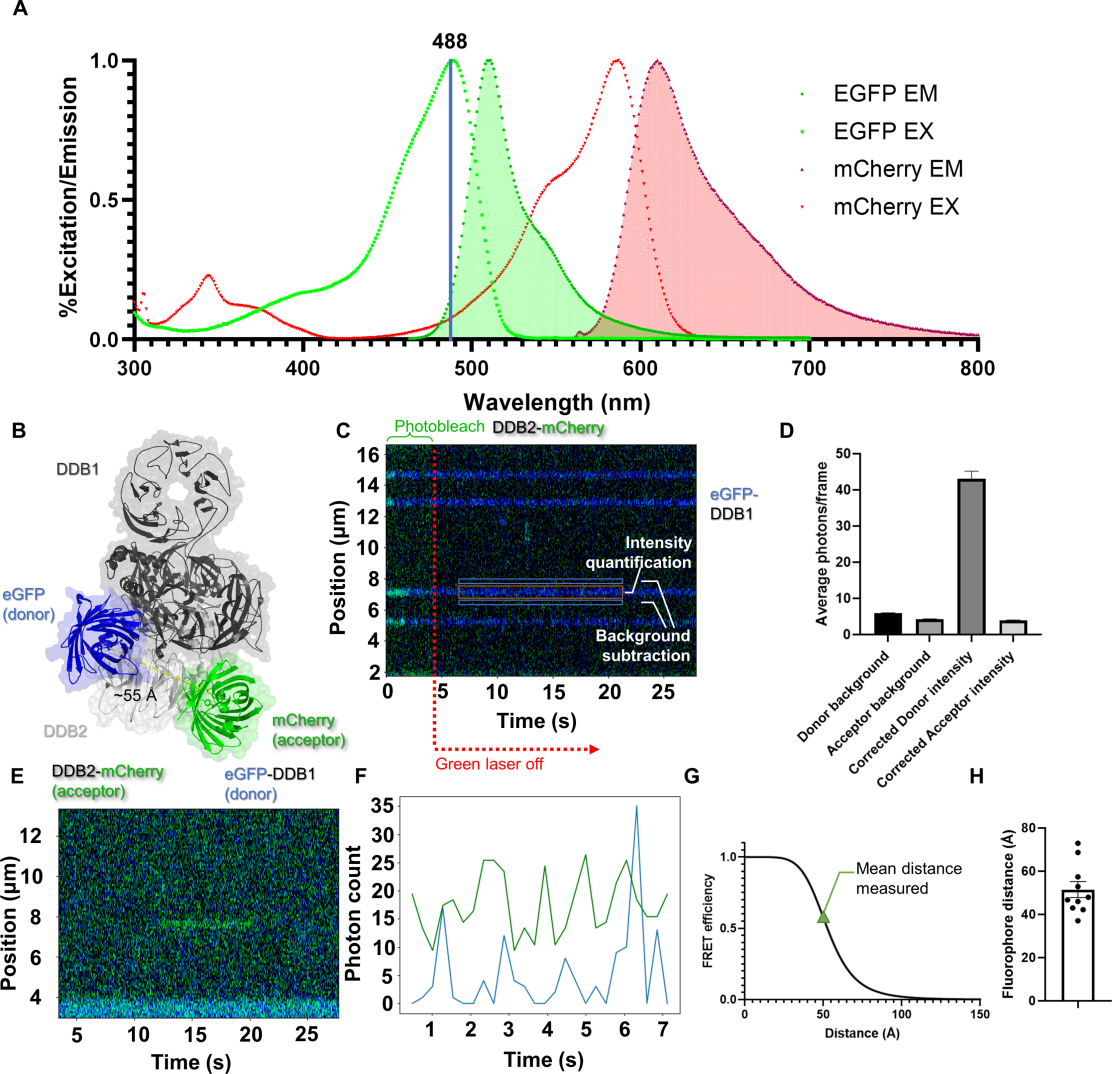
smFRET confirms the direct interaction of DDB1 and DDB2. To probe the structure of colocalized events at resolution beyond the limits of the C-trap, we employed a single-molecule Förster resonance energy transfer (smFRET) approach. (**A**) A diagram of the excitation and emission spectrum of eGFP and mCherry generated from data on FPbase.org. The emission of eGFP overlaps with the excitation of mCherry as necessary for FRET. (**B**) The structure of eGFP-DDB1 (donor) and DDB2-mCherry (acceptor), with fluorophores modeled in at their respective termini. (**C**) An example kymograph of four events to assay eGFP signal in the channel used for mCherry (green). (**D**) A consistent ratio of 9.0% of the eGFP photon counts were observed in the mCherry channel, so that factor was used as a correction factor. (**E**) An example FRET-positive event with quantification of photon counts shown in (**F**). (**G**) With a known Förster radius of eGFP and mCherry, distances were calculated based on the ratiometric FRET efficiency. (**H**) Out of ten FRET positive events (*n* = 10), the average distance between fluorophores was 51.0 Å, in agreement with the structural model shown in (B).

To further probe the structure of these colocalization events, we utilized an DDB2-mCherry construct to act as the acceptor in a single-molecule Förster resonance energy (sm-FRET) approach with eGFP-DDB1 donor (spectral overlap shown in Figure 5A). Based on the structure of UV-DDB with the fluorescent tags modeled in, we expected the distance between the tags to be ∼55 Å in the case of a true UV-DDB heterodimer (Figure 5B). To quantitatively measure fluorescence intensity, the background intensity for the donor and acceptor were measured, as well as quantifying how much a single donor fluorophore emits in the acceptor channel (Figure 5C and D). We observed a clear FRET signal for multiple events (Figure 5E and F). This FRET signal efficiency correlated well with the expected distances from the two fluorophores (Figure 5G) and remained relatively consistent for multiple events (Figure 5H), thus confirming a direct interaction between DDB1 and DDB2.

### Effects of unlabeled protein on fluorescently tagged protein behavior: facilitated dissociation of UV-DDB with purified UV-DDB

**Figure 6.**
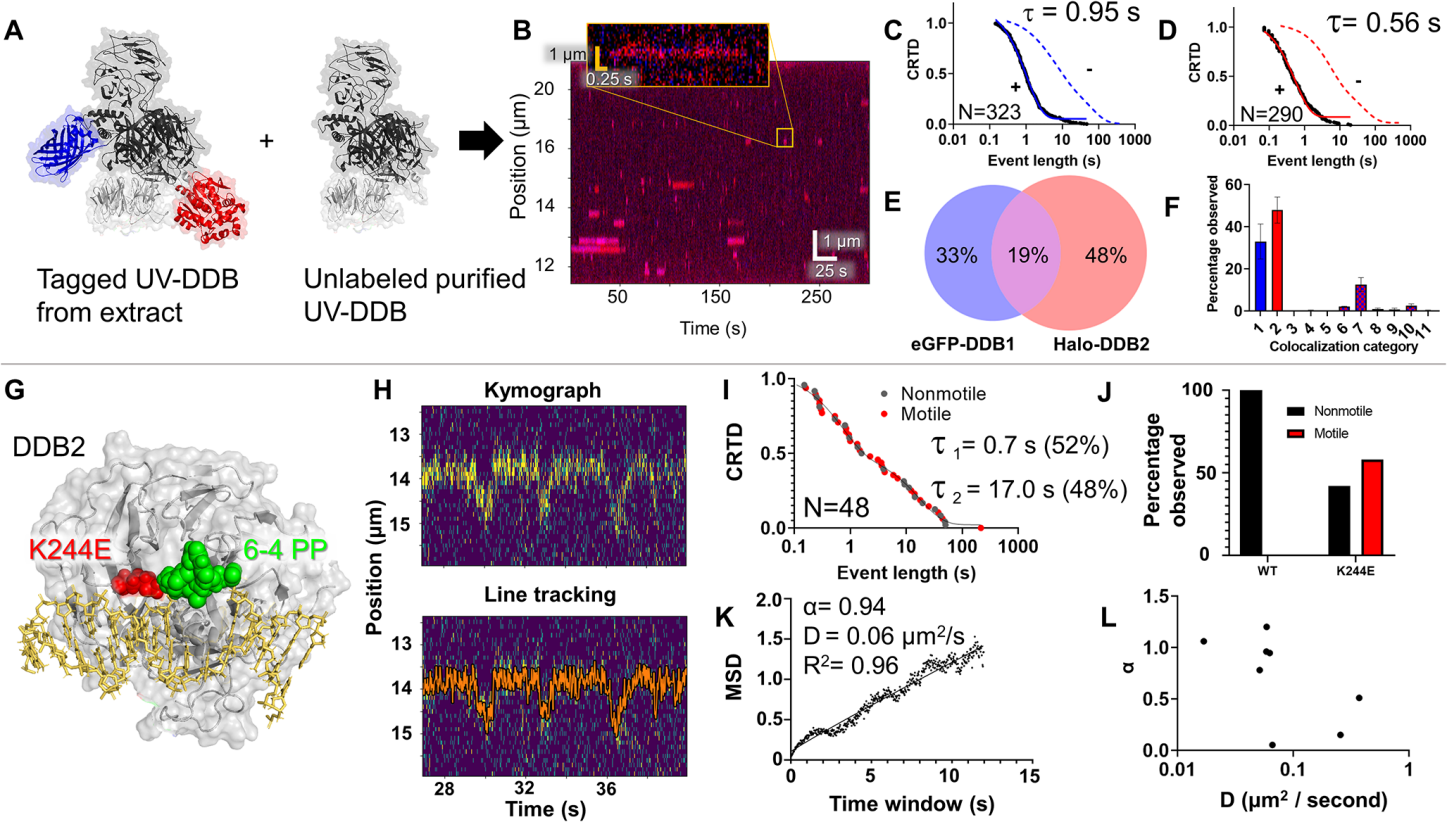
Observing facilitated dissociation and movement behavior of DDB2 K244E. (**A**) Diagram of dual-labeled UV-DDB (with eGFP and HaloTag-JF-635) and unlabeled purified UV-DDB included (PDB ID: 4E5Z, 4EUL, 5UY1). (**B**) An example kymograph of labeled DDB1 and DDB2 binding transiently to UV-damaged DNA. (**C**, **D**) CRTD plots of DDB1 (blue, c) and DDB2 (red, d) shown. Dotted lines indicate CRTD curves without added unlabeled UV-DDB (data from Figure 4). (**E**) Distribution of events that were DDB1 alone, DDB2 alone, or colocalized. (**F**) Colocalization categories for DDB1 and DDB2 binding damaged DNA with error bars as the SEM of three experiments. (**G**) Structure of DDB2 bound to a 6–4 photoproduct (green), with the site of the K244E mutation (red). (**H**) A kymograph of motile DDB2 K244E binding. The tracked position of the line is shown in orange. (**I**) the CRTD plot for all K244E binding events, with motile shown in red and nonmotile events shown in gray. (**J**) the distribution of motile and nonmotile for WT and DDB2 K244E. (**K**) Mean squared displacement analysis of event shown in panel (H). (**L**) Diffusivity (*D*) and α values for K244E events. See Supplementary Figures S6 and S8.

Although *k*_off_ values and thus binding lifetimes are traditionally thought to be concentration independent, a growing body of work has shown that presence of competitor proteins can alter binding lifetimes (54–56). This phenomenon would alter binding results observed by SMADNE if the endogenous unlabeled protein represents a significant fraction compared to the labeled protein of interest. To examine facilitated dissociation of the target labeled protein by the endogenous non-labeled protein, we included tenfold excess concentration of purified UV-DDB (3 nM) along with the eGFP-DDB1 and HaloTag-DDB2 tagged proteins in extracts (Figure 6A). While we observed a similar number of events, the event lifetime was drastically reduced by ∼30-fold for DDB1 and ∼40-fold for DDB2 in the presence of purified protein (Figure 6B–D, Supplementary Table S3). We also tested other concentrations of added unlabeled UV-DDB and saw a concentration-dependent response in binding lifetime (Supplementary Figure S8). Interestingly, we also saw a decrease in colocalization frequency from 32% to 19%, which may suggest that the subunits from purified UV-DDB may exchange in solution; however, category 7 (binding together and dissociating together) was again the most common category (Figure 6E and F). We additionally collected a dataset with overexpressed mNeonGreen-DDB2, and the τ_c_ agreed with that of the HaloTag-DDB2 (42.3 s, Supplementary Figure S7 and Table 1). Of note, we also collected binding parameters for DDB2 expressed at levels only fourfold higher than endogenous levels, and the resultant τ_c_ of 9.2 s was similar to the lifetime when 0.3 nM purified protein was added (Supplementary Figure S7A, D and Supplementary Table S3).

### SMADNE allows rapid characterization of protein variants: DDB2 variant (K244E)

SMADNE offers a rapid means to determine the effects of naturally occurring mutations on function without having to purify the protein, which can reduce yield and activity. To address this goal, we utilized a K244E variant of DDB2 associated with the human syndrome xeroderma pigmentosum complementation group E (Figure 6G). Previous single-molecule characterization of this variant demonstrated this substitution causes UV-DDB to lose specificity for damage sites by diffusing past UV-induced photoproducts (5). Indeed, the mNeonGreen-DDB2 K244E variant exhibited increased motility and decreased binding lifetimes (Figure 6H), with 58% of the events observed exhibiting detectable motion in contrast to 0% with WT DDB2 (Figure 6I and J). MSD analysis of the motile binding events indicated motile mNeonGreen-DDB2 K2444E events exhibited diffusivity values between ∼0.01–1 μ^2^/s, which is slightly more diffusive than previous studies of the variant with Qdot labeling (Figure 6K and L) (5). One reason that the diffusivity could be slower with the purified proteins is that the large Qdot label increases the drag considerably compared to the smaller fusion tag in the SMADNE approach. In addition to the motion along the DNA, shorter binding lifetimes were observed with this mutant compared to our characterization of WT DDB2. The τ_c_ for DDB2 K244E was 24.0 s (compared to the WT mNeonGreen-DDB2 τ_c_ of 42.3 s), which agrees with the hypothesis that the mutation prevents full engagement with the DNA (Figure 6I, Supplementary Figure S7, Table 1, and Supplementary Table S3).

### Visualizing oxidative damage repair dynamics with SMADNE

**Figure 7.**
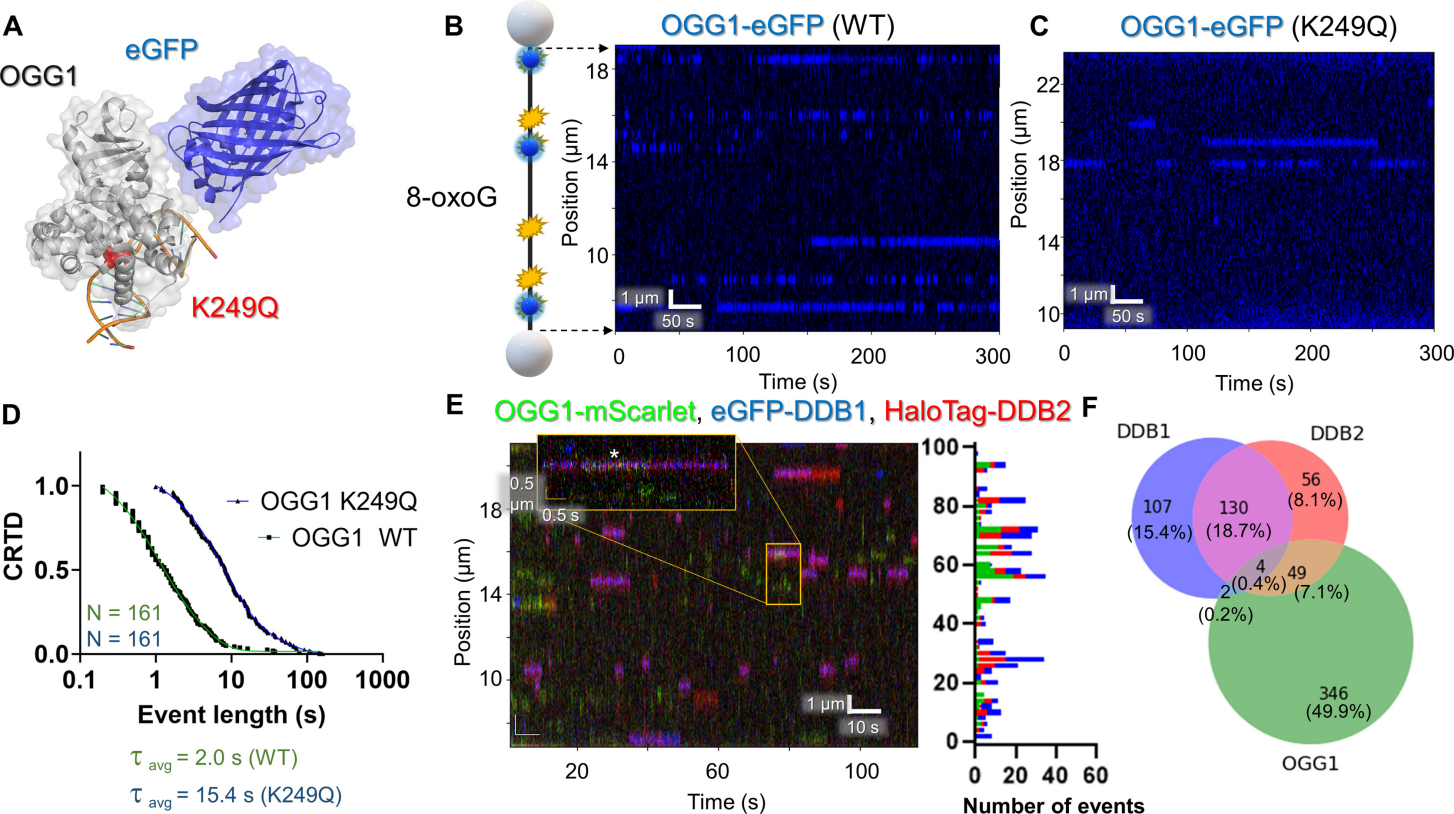
OGG1 and UV-DDB binding to DNA with oxidative damage. (**A**) A structural model of eGFP-tagged OGG1 bound to 8-oxoG containing DNA with K249Q showed in red (PDB codes 1YQR and 5LK4). (**B**) A cartoon depiction of DNA with 8-oxoG damage shown in blue. The accompanying kymograph shows many transient OGG1 binding events on the DNA in blue. (**C**) With the catalytically dead variant K249Q, much longer binding lifetimes were observed (blue). (**D**) CRTD analysis for WT and K249Q OGG1 at 10 pN. The weighted average lifetime for the mutant was 15.4 s (42.9 and 7.7 s, 78% fast), ∼eight-fold longer than the 2.0 s lifetime of the WT. After correcting for photobleaching rate this ratio becomes 23-fold longer for the K249Q variant (Table 1). (**E**) Kymograph of OGG1-mScarlet (green), eGFP-DDB1 (blue) and HaloTag-DDB2 (red) with binding positions shown on the right. (**F**) The distribution of events that bound alone vs colocalizing for all three proteins. See Supplementary Figures S4 and S9.

Using single-molecule and cellular studies, we recently demonstrated that UV-DDB interacts with OGG1 to process 8-oxoG lesions (34). To this end, we first prepared nuclear extracts from OGG1-eGFP expressing cells (Figure 7A) and studied OGG1 binding on DNA treated with oxidative damage (one 8-oxoG/440 bp) (35). OGG1 bound to numerous positions along the length of the DNA, with many positions bound multiple times (at sites of oxidative damage, Figure 7B). Each binding position of OGG1 exhibited similar binding lifetimes: a CRTD plot revealed a best fit to a double-exponential function with a weighted average lifetime of 2.0 s (Figure 7D). We also observed short lifetimes of OGG1 binding to non-damaged DNA, although the frequency of binding was significantly less (Supplementary Figure S4I). These lifetimes agree with the ∼2 s lifetimes published by Wallace and coworkers for purified *E. coli* Fpg (35), and Verdine and colleagues for OGG1 on non-damaged DNA (57). We then tested the binding characteristics of a catalytically dead OGG1 variant containing a mutation in its active site, K249Q (Figure 7C) (58). As compared to WT OGG1, eGFP-labeled OGG1-K249Q bound to a DNA substrate containing 8-oxoG significantly longer with two photobleaching corrected lifetimes of 8.9 and 183 s, for a τ_c_ of 47.2 s (Figure 7D, Table 1, and Supplementary Table S3).

Since we have found that UV-DDB interacts with OGG1 to process 8-oxoG lesions (34), we sought to determine whether these interactions could be observed in nuclear extracts using SMADNE. To this end, we expressed OGG1-mScarlet, which behaves similarly to OGG1-eGFP but is an alternate color compatible with the dual-labeled UV-DDB (Supplementary Figure S7E–G), eGFP-DDB1, and HaloTag-JF635-DDB2, and observed interactions between all three proteins (Figure 7E and F). UV-DDB bound to DNA with oxidative damage robustly, but the binding lifetimes of DDB2 (0.14 s) were reduced compared to their lifetime on UV damage, in agreement with its lower affinity to 8-oxoG compared to UV damage (Supplementary Figure S9, Supplementary Table S3) (34). Furthermore, we did observe a moderate degree of transient colocalization between DDB2 and OGG1, but the majority of binding events were either OGG1 alone or DDB1 and DDB2 together at 49.9% and 15.4%, respectively (Figure 7F).

## DISCUSSION

SMADNE offers several major advantages compared to traditional single-molecule studies in living cells or with purified proteins. First, nuclear extracts used in SMADNE rapidly generate similar mechanistic information in agreement with previous work using purified proteins (including binding lifetimes and other outcomes shown in Figure 1). Second, since SMADNE can utilize common fluorescence tags, such as eGFP, nuclear extracts could be rapidly prepared from transfection of commercially available overexpression plasmids. One potential limitation is that proteins that do not overexpress well or are unstable in the absence of a partner may represent poor candidates for SMADNE characterization. However, overexpression can be optimized in various ways, such as expressing unlabeled binding partners at the same time (as with XPC-RAD23B) to increase overexpression levels. Additionally, if an affinity tag were to be utilized in conjunction with a fluorescent tag (e.g. FLAG or 6xHIS tag), proteins that express poorly could be enriched with an affinity pulldown. Adding an enrichment step provides the advantage of sampling higher concentrations of protein and likely would make the enriched proteins more uniform compared to SMADNE without enrichment. However, a disadvantage of this setup is that other proteins that may influence binding or stability in a physiological system may be lost, and adding this step makes the method slightly more time consuming and would require more cells and reagents.

Another potential limitation, as observed with live cell imaging, SMADNE may be influenced by unlabeled endogenous proteins, which is not the case with other single-molecule approaches with purified proteins. We found that transient transfection overexpressed our proteins of interest 50 to 100-fold higher than endogenous levels, minimizing these interactions. Alternately, fluorescent proteins can be expressed near endogenous levels as in Supplementary Figure S7C and F. At the lower expression level, we observed that the competition increased and thus the τ_c_ decreased ∼four-fold to 9.2 s. However, with this method the influence of potential interacting proteins could be studied via specific knockouts or inhibitors (42) (Extended Data Table 1). Third, orthologous labeling allowed co-localization studies to be performed on heterodimeric complexes and interacting proteins. Fourth, SMADNE enables a wide range of interaction affinities to be studied, even transient interactions with *K*_D_ values of ∼1 μM. Because the *k*_off_ correlates with binding lifetime, a *K*_D_ value of ∼1 μM appears to be the limit of detection using SMADNE – binding events weaker than this would have a lifetime of <0.1 s and be challenging to detect. Finally, the work on the UV-DDB and OGG1 variants indicate that SMADNE will allow analysis of structural variants for proteins of interest via site-directed mutagenesis of specific residues and will facilitate rapid screening of variants of unknown significance associated with tumors (COSMIC data base: https://cancer.sanger.ac.uk/cosmic).

Other methods exist that have been used to characterize proteins, RNA, and DNA at the single-molecule scale from extracts. These include Comparative Colocalization Single-Molecule Spectroscopy (CoSMoS) to study RNA-protein interactions out of yeast extracts (17,18), *Xenopus laevis* egg extracts to study DNA replication and repair (19–21), and single-molecule pulldown (SiMPull) to analyze protein complex stoichiometry and binding parameters from pulled-down proteins, among other techniques (22–24). These single-molecule methods all represent major advances in bridging the gap between cellular and single-molecule studies by studying cell extracts at the single-molecule level. SMADNE synthesizes elements from previous techniques as it, for the first time, uses human nuclear extracts to visualize protein binding on DNA strands in relation to defined genomic position and generates invaluable mechanistic information in an environment containing physiologically-relevant nuclear proteins. In this way post-translational modification of desired proteins after specific signaling events (e.g. DNA damage responses) can be monitored. Furthermore, performing SMADNE on the LUMICKS C-trap overcomes a disadvantage to single-molecule approaches requiring TIRF microscopy that utilize DNA tethered to the bottom of the flow cells: nuclear debris can also stick to the bottom of flow chambers and obscure/overpower the fluorescence of single molecules. Despite this disadvantage, other single-molecule techniques with DNA near the bottom of the chamber, like the DNA curtains assay, can also be utilized with nuclear extracts, as long as care is taken to filter out surface binding events of cell debris from true DNA binding events (via careful quantitation of fluorescence intensity and spot sizes) and maximizing photostability (30,59). In cases where binding events are rare or transient, these alternate assays have the advantage of visualizing hundreds of DNA molecules simultaneously, although inconsistent tension between the strands of DNA could also be a confounding factor. In contrast, with SMADNE on the LUMICKS C-trap, the DNA strand remains in the center of the flow cell, circumventing debris accumulation in its focal plane and generating clear binding events.

SMADNE substantially lowers the barrier of entry for numerous research groups to understand their DNA-binding proteins of interest at the single-molecule level without the burden of protein purification. While the applications shown here focus on DNA repair proteins, many other types of DNA-binding proteins would also apply, including transcription factors, helicases, and DNA polymerases. Furthermore, this new approach could be used to observe macromolecular interactions from extracts generated from a wide range of cells and tissues from animals expressing fluorescent proteins. With the rapid workflow of plasmid transfection to single-molecule data collection, SMADNE creates the possibility to screen numerous disease-associated protein variants in a high-throughput manner previously unattainable with purified proteins. Hence, SMADNE performed in conjunction with the LUMICKS C-trap represents a novel, scalable, and relatively high-throughput method to obtain single-molecule mechanistic insights into key protein–DNA interactions in an environment containing physiologically-relevant nuclear proteins.

## DATA AVAILABILITY

Python scripts used to calculate photobleaching decay constants and colocalization analysis have been deposited at https://harbor.lumicks.com/scripts. Raw data for kymograph images and force measurements are available upon request.

## Supplementary Material

gkad095_Supplemental_Files
