# Supplementary material for: Single-molecule analysis of DNA-binding proteins from nuclear extracts (SMADNE)
Source: Nucleic Acids Res. 2023 Mar 2;51(7):e39. doi: 10.1093/nar/gkad095 (PMC10123111; doi:10.1093/nar/gkad095)
Supplement: gkad095_Supplemental_Files [file gkad095_supplemental_files.zip › C-trap analysis template-V28-red-and-blue-colocalization-analyzer.html]

C-trap analysis template-V28-red-and-blue-coloc-cleaned


In [1]:

```
%%javascript
IPython.OutputArea.prototype._should_scroll = function(lines) {
    return false;
}
```

This tool will track lines in the red (638 nm) and blue (488 nm) channels, and determine the binding lifetimes and orders of assembly and disassembly of the events.

In [2]:

```
import matplotlib.pyplot as plt
import lumicks.pylake as lk
import itertools
import ipywidgets
import numpy as np
import glob
import os
import shutil
# We use skimage to downsample the data
from skimage.measure import block_reduce
from statistics import mean
from statistics import median
from scipy.stats import sem
from matplotlib_venn import venn2 #this line is used for making venn diagrams of interactions
%matplotlib inline
# Use notebook if you're in Jupyter Notebook
%matplotlib notebook
```

In [3]:

```
filename = glob.glob('*.h5') #fetches filename assuming only one .h5 in notebook
if len(filename) == 1:
    print (filename[0])
else: 
    print ("too many or too few .h5 files")
```

```
20211023-154631 Kymograph 3.h5
```

In [4]:

```
plt.close('all')#This closes any plots left open to save memory and loads up the .h5 file in folder
file = lk.File(str(filename[0]))
list(file.kymos)
```

Out[4]:

```
['2', '3', '4']
```

In [5]:

```
forcex = file["Force HF"]["Force 1x"] #this cell downsamples the force to save memory and make it easier to visualize

# time traces (seconds)
time = forcex.timestamps/1e9
time = time - time[0]
sample_rate = forcex.sample_rate

downsampled_rate = 50 # Hz, this rate can be changed as needed

# downsample the force, nanostage position and time
forcex_downsamp = forcex.downsampled_by(int(sample_rate/downsampled_rate))
time_downsamp = forcex_downsamp.timestamps/1e9
time_downsamp = time_downsamp - time_downsamp[0]

median_force = (median(forcex_downsamp.data))
```

In [6]:

```
from kymowidget import KymoWidgetGreedy #imports the widget for kymograph analysis
```

In [7]:

```
_, kymo = file.kymos.popitem() #after defining the downsample rate earlier, this actually does the downsampling

data = file.kymos["3"].blue_image #note this depends on the collection channel -- for Cy3 we use green
downsample_factor = 1
data = block_reduce(data, (1, downsample_factor))
#rgb = kymo.rgb_image
kymo = file.kymos["3"]
```

In [8]:

```
dt = downsample_factor * kymo.line_time_seconds
```

In [10]:

```
plt.figure(figsize=(7, 4)) #this plots the kymograph side-by-side with the force

# Plot the kymograph
ax1 = plt.subplot(2, 1, 1)
plt.title('UV-DDB binding UV-damaged lambda DNA')
# We use aspect="auto" because otherwise the kymo would be very long and thin
kymo.plot_red(vmax=1, aspect="auto")
plt.ylim(19, 0)

# Plot the force
ax2 = plt.subplot(2, 1, 2, sharex = ax1)
forcex_downsamp.plot(color='red')
plt.ylabel('Force (pN)')
plt.tight_layout()
plt.title('DNA tension')
ax2.set_facecolor('gray')
plt.show()
```

In [11]:

```
print(dt)
```

```
0.10000640000000001
```

In [12]:

```
pixel_size = kymo.pixelsize_um
pixel_size_int = pixel_size[0]
```

In [13]:

```
print(pixel_size_int)
```

```
0.1
```

In [14]:

```
kw = KymoWidgetGreedy(data, axis_aspect_ratio=2, min_length=4, pixel_threshold=5, window=7, sigma=1, vmax=2)
#this first plot is to look at the kymograph on the pixel scale in order to decide if an upper and lower bound need to be applied
```

In [15]:

```
#Trim the data to avoid the beads by defining upper and lower bounds
lower_bound= 28
adjusted_lower_bound = pixel_size_int * lower_bound
upper_bound= 180
adjusted_upper_bound = pixel_size_int * upper_bound
data = data[lower_bound:upper_bound, :] 
kymo.red_data = kymo.red_image[lower_bound:upper_bound, :]
kymo.green_data = kymo.green_image[lower_bound:upper_bound, :]
steps_between_bounds = int((adjusted_upper_bound - adjusted_lower_bound) * 10) #defines number of 100 nm steps between bounds
kymo.blue_data = kymo.blue_image[lower_bound:upper_bound, :]
plt.close('all')

#Define a downsample factor here to downsample data if needed. Note that all colors must be downsampled the same ratio for the
#colocalization analysis to work properly

downsample_factor = 2
```

In [16]:

```
red_lines = lk.KymoWidgetGreedy(kymo.downsampled_by(downsample_factor), "red", aspect="auto", min_length=4, line_width=0.5, pixel_threshold=1, window=7, sigma=0.25, vmax=2)
```

In [17]:

```
red_lines.save_lines("redkymotracks.txt", sampling_width=3)
```

In [18]:

```
blue_lines = lk.KymoWidgetGreedy(kymo.downsampled_by(downsample_factor), "blue", aspect="auto", min_length=4, line_width=0.5, pixel_threshold=1, window=12, sigma=0.25, vmax=2)
```

In [19]:

```
blue_lines.save_lines("bluekymotracks.txt", sampling_width=3)
```

In [20]:

```
#this cell outputs the number of lines in the kymo and also defines number of bins based on events^1/2
#if the kymotracking didn't work, the number will be 0
numberofredlines = len(red_lines.lines)
print (numberofredlines)
numberofredbins = int(round(np.sqrt (numberofredlines)) )
print (numberofredbins)
#below section for blue lines
numberofbluelines = len(blue_lines.lines)
print (numberofbluelines)
numberofbluebins = int(round(np.sqrt (numberofbluelines)) )
print (numberofbluebins)
```

```
16
4
18
4
```

In [21]:

```
#RED LINES SECTION--------------------------------------------------------------
redlengths = [len(line) for line in red_lines.lines]
# Get the index of the longest kymo line
redlongest_index = np.argmax(redlengths)

# Select the longest red line
redlongest_line = red_lines.lines[redlongest_index]

print(redlongest_index)
#this section plots the positions of the longest line and also shows the raw image of that line 
#also leaves 1 second empty on each side so the start and end can be seen.
plt.figure(figsize=(5, 3))
plt.plot(np.array(redlongest_line.time_idx) * dt, np.array(redlongest_line.coordinate_idx ) * pixel_size + adjusted_lower_bound) 
plt.xlabel('Time [s]')
plt.ylabel('Position [$\mu$m]')
plt.tight_layout()
plt.show()

plt.figure(figsize=(7, 4))
ax1 = plt.subplot(2, 1, 1)
kymo.plot_red(vmax=3, aspect="auto")
ax1.set_xlim([(np.array(redlongest_line.time_idx[0])) * dt -1, (np.array(redlongest_line.time_idx[-1]) * dt +1 )])
ax1.set_ylim(adjusted_upper_bound+1, adjusted_lower_bound-1) 

#BLUE LINES SECTION--------------------------------------------------------------
bluelengths = [len(line) for line in blue_lines.lines]
# Get the index of the longest kymo line
bluelongest_index = np.argmax(bluelengths)

# Select the longest blue line
bluelongest_line = blue_lines.lines[bluelongest_index]

print(bluelongest_index)
#this section plots the positions of the longest line and also shows the raw image of that line 
#also leaves 1 second empty on each side so the start and end can be seen.
plt.figure(figsize=(5, 3))
plt.plot(np.array(bluelongest_line.time_idx) * dt, np.array(bluelongest_line.coordinate_idx ) * pixel_size + adjusted_lower_bound) 
plt.xlabel('Time [s]')
plt.ylabel('Position [$\mu$m]')
plt.tight_layout()
plt.show()

plt.figure(figsize=(7, 4))
ax1 = plt.subplot(2, 1, 1)
kymo.plot_blue(vmax=3, aspect="auto")
ax1.set_xlim([(np.array(bluelongest_line.time_idx[0])) * dt -1, (np.array(bluelongest_line.time_idx[-1]) * dt +1 )])
ax1.set_ylim(adjusted_upper_bound+1, adjusted_lower_bound-1)
```

```
14
```

```
5
```

Out[21]:

```
(19.0, 1.8000000000000003)
```

In [23]:

```
#THIS CELL FOR PROCESSING RED CHANNEL DATA
#to plot photon count along the longest line
plt.figure(figsize=(6, 5))
plt.plot( np.array(redlongest_line.time_idx) * dt, redlongest_line.sample_from_image(3)) #this samples 3 pixels away from line position
plt.ylabel('Photon count')
plt.xlabel('Time [s]')
plt.title('Photon counts along the longest line')
plt.tight_layout()
plt.show()

#fits the data to a single exponential decay
from scipy.optimize import curve_fit
def func(x, a, b, c,d):
    return a * np.exp(-b * (x-d)) + c 
redtimes = np.array([(line.time_idx[-1]-line.time_idx[0]) for line in red_lines.lines])*dt*downsample_factor
counts,bins=np.histogram(redtimes, bins=numberofredbins)
binscenters = np.array([0.5 * (bins[i] + bins[i+1]) for i in range(len(bins)-1)])
popt, pcov = curve_fit(func, binscenters, counts, bounds=(0, [500, 1, 10, 5]))

plt.figure(figsize=(5,3))
plt.plot(binscenters,counts,'.')
plt.plot(binscenters, func(binscenters, *popt), 'r-',

         label='fit: a=%5.3f, b=%5.3f, c=%5.3f, d=%5.3f' % tuple(popt))
redlifetime = 1 / popt[1]
print (redlifetime)
plt.legend()
plt.show()
# for this dataset, this translates to a t1/2 of 1 s (1/b = 1 s)

#plots line length as a histogram. numberofbins was defined earlier as square root of n

plt.figure(figsize=(5, 3))
plt.plot(np.flipud(np.sort (redtimes)))
plt.ylabel('Events')
plt.xlabel('Duration (s)')
plt.title('Lengths of events')
plt.tight_layout()
plt.show()

#adjust redtimes by downsampling factor
adjusted_redtimes = redtimes*downsample_factor

#histogram of starting positions for each line
redtime = [(line.time_idx[0]) * dt for line in red_lines.lines]
##np.array(longest_line.coordinate_idx) * pixel_size / 1000
startpositions = np.array([line.coordinate_idx[0] for line in red_lines.lines]) * pixel_size + adjusted_lower_bound
plt.figure(figsize=(5, 3))
plt.hist(startpositions, 214) #Arbitrarily defined a bunch of bins on this, roughly corresponding to 100 nm. This can be altered.
plt.xlabel('Start Position (um from top)')
plt.ylabel('Number of events')
plt.tight_layout()
plt.show()


#histogram of mean positions for each line
redmeanpositions = np.array([np.average(line.coordinate_idx) for line in red_lines.lines]) * pixel_size + adjusted_lower_bound
plt.figure(figsize=(5, 3))
plt.hist(redmeanpositions, 214)
plt.xlabel('Average position (um from top)')
plt.ylabel('Times occuring')
plt.tight_layout()
plt.show()

#histogram of localization precision in um
redstandarddeviation = np.array([np.std(line.coordinate_idx, ddof=1) for line in red_lines.lines]) * pixel_size
plt.figure(figsize=(5, 3))
plt.hist(redstandarddeviation, numberofredbins)
plt.xlabel('Localization precision (um)')
plt.ylabel('Times occuring')
plt.tight_layout()
plt.show()
#this saves the MSD vlues as a .txt file in case we want to graph these in excel/prism. Still under development.
np.savetxt("redstd.txt", 
           redstandarddeviation,
           delimiter =", ",
            fmt = "%s")

#histogram of normalized line positions in terms of percentage along DNA bound. 
#To function properly, upper and lower bound must be defined as the edge of the beads.
rednorm_meanpositions = np.array([np.average(line.coordinate_idx-lower_bound) for line in red_lines.lines])  /(upper_bound-lower_bound) * 100
plt.figure(figsize=(5, 3))
plt.hist(rednorm_meanpositions, steps_between_bounds, color='blue')
plt.xlabel('Normalized average position (percent from top)')
plt.xlim(0, 100)
plt.ylabel('Times occuring')
plt.tight_layout()
plt.show()

#histogram of the difference between the start and endpoint of each line
reddistancetraveled = np.array([line.coordinate_idx[-1]-line.coordinate_idx[0] for line in red_lines.lines]) * pixel_size
plt.figure(figsize=(5, 3))
plt.hist(reddistancetraveled, numberofredbins)
plt.ylabel('Event number')
plt.xlabel('Distance traveled (um)')
plt.tight_layout()
plt.show()


#histogram of the absolute value of the difference between the start and endpoint of each line
redabsdistancetraveled = np.array([abs(line.coordinate_idx[-1]-line.coordinate_idx[0]) for line in red_lines.lines]) * pixel_size
plt.figure(figsize=(5, 3))
plt.hist(redabsdistancetraveled, numberofredbins)
plt.xlabel('Absolute value of distance traveled (um)')
plt.ylabel('Number of events')
plt.tight_layout()
plt.show()
```

```
C:\Users\schaichm\Anaconda3\envs\pylake3_9\lib\site-packages\scipy\optimize\minpack.py:833: OptimizeWarning: Covariance of the parameters could not be estimated
  warnings.warn('Covariance of the parameters could not be estimated',
```

```
26.856840366118952
```

In [25]:

```
#THIS CELL FOR PROCESSING BLUE CHANNEL DATA
#to plot photon count along the longest line
plt.figure(figsize=(6, 5))
plt.plot( np.array(bluelongest_line.time_idx) * dt, bluelongest_line.sample_from_image(3)) #this samples 3 pixels away from line position
plt.ylabel('Photon count')
plt.xlabel('Time [s]')
plt.title('Photon counts along the longest line')
plt.tight_layout()
plt.show()

#fits the data to a single exponential decay
from scipy.optimize import curve_fit
def func(x, a, b, c,d):
    return a * np.exp(-b * (x-d)) + c 
bluetimes = np.array([(line.time_idx[-1]-line.time_idx[0]) for line in blue_lines.lines])*dt*downsample_factor 
counts,bins=np.histogram(bluetimes, bins=numberofbluebins)
binscenters = np.array([0.5 * (bins[i] + bins[i+1]) for i in range(len(bins)-1)])
popt, pcov = curve_fit(func, binscenters, counts, bounds=(0, [5000, 1, 5, 0.01]))

plt.figure(figsize=(5,3))
plt.plot(binscenters,counts,'.')
plt.plot(binscenters, func(binscenters, *popt), 'r-',

         label='fit: a=%5.3f, b=%5.3f, c=%5.3f, d=%5.3f' % tuple(popt))
bluelifetime = 1 / popt[1]
print (bluelifetime)
plt.legend()
plt.show()
# for this dataset, this translates to a t1/2 of 1 s (1/b = 1 s)

#adjust bluetimes by downsampling factor
adjusted_bluetimes = bluetimes*downsample_factor

#plots line length as a histogram. numberofbins was defined earlier as square root of n
plt.figure(figsize=(5, 3))
plt.hist(bluetimes, numberofbluebins)
plt.ylabel('Events')
plt.xlabel('Duration (s)')
plt.title('Lengths of events')
plt.tight_layout()
plt.show()


#histogram of starting positions for each line
bluetime = [(line.time_idx[0]) * dt for line in blue_lines.lines]
##np.array(longest_line.coordinate_idx) * pixel_size / 1000
startpositions = np.array([line.coordinate_idx[0] for line in blue_lines.lines]) * pixel_size + adjusted_lower_bound
plt.figure(figsize=(5, 3))
plt.hist(startpositions, 214) #Arbitrarily defined a bunch of bins on this, roughly corresponding to 100 nm. This can be altered.
plt.xlabel('Start Position (um from top)')
plt.ylabel('Number of events')
plt.tight_layout()
plt.show()


#histogram of mean positions for each line
bluemeanpositions = np.array([np.average(line.coordinate_idx) for line in blue_lines.lines]) * pixel_size + adjusted_lower_bound
plt.figure(figsize=(5, 3))
plt.hist(bluemeanpositions, 214)
plt.xlabel('Average position (um from top)')
plt.ylabel('Times occuring')
plt.tight_layout()
plt.show()

#histogram of localization precision in um
bluestandarddeviation = np.array([np.std(line.coordinate_idx, ddof=1) for line in blue_lines.lines]) * pixel_size
plt.figure(figsize=(5, 3))
plt.hist(bluestandarddeviation, numberofbluebins)
plt.xlabel('Localization precision (um)')
plt.ylabel('Times occuring')
plt.tight_layout()
plt.show()
#this saves the MSD vlues as a .txt file in case we want to graph these in excel/prism. Still under development.
np.savetxt("bluestd.txt", 
           bluestandarddeviation,
           delimiter =", ",
            fmt = "%s")

#histogram of normalized line positions in terms of percentage along DNA bound. 
#To function properly, upper and lower bound must be defined as the edge of the beads.
bluenorm_meanpositions = np.array([np.average(line.coordinate_idx-lower_bound) for line in blue_lines.lines])  /(upper_bound-lower_bound) * 100
plt.figure(figsize=(5, 3))
plt.hist(bluenorm_meanpositions, steps_between_bounds, color='blue')
plt.xlabel('Normalized average position (percent from top)')
plt.xlim(0, 100)
plt.ylabel('Times occuring')
plt.tight_layout()
plt.show()

#histogram of the difference between the start and endpoint of each line
bluedistancetraveled = np.array([line.coordinate_idx[-1]-line.coordinate_idx[0] for line in blue_lines.lines]) * pixel_size
plt.figure(figsize=(5, 3))
plt.hist(bluedistancetraveled, numberofbluebins)
plt.ylabel('Event number')
plt.xlabel('Distance traveled (um)')
plt.tight_layout()
plt.show()


#histogram of the absolute value of the difference between the start and endpoint of each line
blueabsdistancetraveled = np.array([abs(line.coordinate_idx[-1]-line.coordinate_idx[0]) for line in blue_lines.lines]) * pixel_size
plt.figure(figsize=(5, 3))
plt.hist(blueabsdistancetraveled, numberofbluebins)
plt.xlabel('Absolute value of distance traveled (um)')
plt.ylabel('Number of events')
plt.tight_layout()
plt.show()
```

```
C:\Users\schaichm\Anaconda3\envs\pylake3_9\lib\site-packages\scipy\optimize\minpack.py:833: OptimizeWarning: Covariance of the parameters could not be estimated
  warnings.warn('Covariance of the parameters could not be estimated',
```

```
34.80335735904268
```

```
C:\Users\schaichm\AppData\Local\Temp/ipykernel_9080/436842969.py:67: RuntimeWarning: More than 20 figures have been opened. Figures created through the pyplot interface (`matplotlib.pyplot.figure`) are retained until explicitly closed and may consume too much memory. (To control this warning, see the rcParam `figure.max_open_warning`).
  plt.figure(figsize=(5, 3))
```

In [26]:

```
#Colocalization analysis
colocalization_count = 0
interaction_tuple = []
print (numberofredlines) 
print (numberofbluelines)
interaction_window = 2 #distance in pixels for an interaction to occur
time_window = 8 #distance in frames for an interaction to occur
#the following loop iterates over the length of each line, comparing every coordinate to the coordinate of all lines of the other color
#if a point between both is closer than the interaction window and occurs within the time window, the interacting lines will
#be appended to the interaction tuple as coordinates
for l in range (0, numberofredlines):
    redlinelength = len((red_lines.lines[l].coordinate_idx))
    for i in range (0, redlinelength):
        for j in range (0, numberofbluelines):
            bluelinelength = len((blue_lines.lines[j].coordinate_idx))
            for k in range (0, bluelinelength):
                if abs(blue_lines.lines[j].coordinate_idx[k]-red_lines.lines[l].coordinate_idx[i]) <= interaction_window and abs(blue_lines.lines[j].time_idx[k] - red_lines.lines[l].time_idx[i]) <= time_window: 
                
                    colocalization_count = colocalization_count + 1
                    #print (l, j)
                    if len(interaction_tuple) > 0:
                        if j != interaction_tuple [len(interaction_tuple)-1][1]:
                            interaction_tuple.append((l, j))

                            break
                        break
                    else: interaction_tuple.append((l,j))
                break

            

        
print (interaction_tuple) #note that this is ordered as (red line index, blue line index)
print (len(interaction_tuple))
```

```
16
18
[(3, 4), (5, 11), (7, 12), (8, 13), (9, 9), (11, 10), (12, 15), (12, 16)]
8
```

In [27]:

```
#interaction statistics
total_interactions=len(interaction_tuple)
print (len(interaction_tuple), 'total interactions')
redinteractions = 1 #there has to be at least one interaction to start with, to compare for unique interactions
if len(interaction_tuple) >0: # this statement checks that there is indeed 1 or more interactions. If not it sets the count to 0
    for i in range (0, len(interaction_tuple)-1):
        if interaction_tuple[i][0] != interaction_tuple[i+1][0]:
            redinteractions = redinteractions + 1
else:
    redinteractions = 0

blueinteractions = 1 #same as above but for blue lines
if len(interaction_tuple) >0:
    for k in range (0, len(interaction_tuple)-1):
        if interaction_tuple[k][1] != interaction_tuple[k+1][1]:
            blueinteractions = blueinteractions + 1
else:
    blueinteractions = 0
    
print (redinteractions)
print (blueinteractions)
```

```
8 total interactions
7
8
```

In [28]:

```
#further colocalization patterns -- of the colocalizations, how many start with red, how many start with blue, how many together?
plt.close('all')
timing_window = 6 #allowed window for events to occur at the same time
interaction_starts = [] #if blue comes on first, this is 0, if they start together: 1, if red is first: 2
for i in range (0, len(interaction_tuple)):
    if ((blue_lines.lines[interaction_tuple[i][1]].time_idx[0]-red_lines.lines[interaction_tuple[i][0]].time_idx[0]) > timing_window):
        interaction_starts.append(2)
    if (abs(blue_lines.lines[interaction_tuple[i][1]].time_idx[0]-red_lines.lines[interaction_tuple[i][0]].time_idx[0]) <= timing_window):
        interaction_starts.append(1)
    if ((red_lines.lines[interaction_tuple[i][0]].time_idx[0]-blue_lines.lines[interaction_tuple[i][1]].time_idx[0]) > timing_window):
        interaction_starts.append(0)

print (interaction_starts)
print ('From',len(interaction_starts), 'interactions')

#further colocalization patterns -- of the colocalizations, how many end with red, how many end with blue, how many together?

timing_window = 6 #allowed window for events to occur at the same time
interaction_ends = [] #if blue leaves first, this is 0, if they leave together: 1, if red leaves first: 2
for i in range (0, len(interaction_tuple)):
    if ((blue_lines.lines[interaction_tuple[i][1]].time_idx[-1]-red_lines.lines[interaction_tuple[i][0]].time_idx[-1]) > timing_window):
        interaction_ends.append(2)
    if (abs(blue_lines.lines[interaction_tuple[i][1]].time_idx[-1]-red_lines.lines[interaction_tuple[i][0]].time_idx[-1]) <= timing_window):
        interaction_ends.append(1)
    if ((red_lines.lines[interaction_tuple[i][0]].time_idx[-1]-blue_lines.lines[interaction_tuple[i][1]].time_idx[-1]) > timing_window):
        interaction_ends.append(0)

print (interaction_ends)
print ('From',len(interaction_ends), 'interactions')


redstart = interaction_starts.count(2)
doublestart = interaction_starts.count(1)
bluestart = interaction_starts.count(0)


redend = interaction_ends.count(0)
doubleend = interaction_ends.count(1)
blueend = interaction_ends.count(2)
print (blueend)
# this section quantifies these phenomena to make venn diagrams based on what color starts and what color ends
# basically this is a rough way of displaying order of assembly and dissassembly
venn2(subsets = (redstart, bluestart, doublestart), set_labels = ('Halo-DDB2', 'eGFP-DDB1'), set_colors=('red', 'blue'));
plt.title("Colocalization starting color")
plt.show()
#this section generates a table categorizing each ternary event into 1 of 9 possible categories
# in other words, this is a more rigorous way of showing order of assembly and dissassembly
#this list starts as 3 as category 1 and 2 represent binary events

interaction_categories = []

for i in range (0, len(interaction_tuple)):
    if interaction_starts[i] == 0:
        if interaction_ends[i] == 0:
            interaction_categories.append(3)
        if interaction_ends[i] == 1: 
            interaction_categories.append(4)
        if interaction_ends[i] == 2: 
            interaction_categories.append(5)    
    if interaction_starts[i] == 1:
        if interaction_ends[i] == 0:
            interaction_categories.append(6)
        if interaction_ends[i] == 1: 
            interaction_categories.append(7)
        if interaction_ends[i] == 2: 
            interaction_categories.append(8) 
    if interaction_starts[i] == 2:
        if interaction_ends[i] == 0:
            interaction_categories.append(9)
        if interaction_ends[i] == 1: 
            interaction_categories.append(10)
        if interaction_ends[i] == 2: 
            interaction_categories.append(11)
            
print (interaction_categories)
```

```
[1, 1, 1, 1, 1, 2, 1, 2]
From 8 interactions
[1, 1, 1, 1, 2, 1, 0, 1]
From 8 interactions
1
```

```
[7, 7, 7, 7, 8, 10, 6, 10]
```

In [29]:

```
plt.figure(figsize=(7, 4))
venn2(subsets = (redend, blueend, doubleend), set_labels = ('Halo-DDB2', 'eGFP-DDB1'), set_colors=('red', 'blue'));
plt.title("Colocalization ending color")
plt.show()
#what color ends a colocalization event, i.e. the last color on
```

In [30]:

```
#colocalization lifetimes 
#determines how long interactions occur before one or both partners leaves
#-- note from MAS -- probably need to go back and do this in a more sophisticated way
# as is this does not account for movement and assumes 1 colocalization for each set of lines

blue_red_colocalization_lifetimes = []
for i in range (0, len(interaction_tuple)):
    if interaction_starts[i] == 0: # this means that blue came on first, so the start of the colocalization is the first red time
        coloc_start = red_lines.lines[interaction_tuple[i][0]].time_idx[0]
    if interaction_starts[i] == 1: # this means that both colors came on together. For now we'll just take the average start.
        coloc_start = ((red_lines.lines[interaction_tuple[i][0]].time_idx[0] + blue_lines.lines[interaction_tuple[i][1]].time_idx[0])/2)
    if interaction_starts[i] == 2: # this means that red came on first, so the start of the colocalization is the first blue time
        coloc_start = blue_lines.lines[interaction_tuple[i][1]].time_idx[0]
    if interaction_ends[i] == 0: # this means that red left last, so the end of the colocalization is the last blue timepoint
        coloc_end = blue_lines.lines[interaction_tuple[i][1]].time_idx[-1]
    if interaction_ends[i] == 1: # this means that both colors left together. For now we'll just take the average end.
        coloc_end = ((red_lines.lines[interaction_tuple[i][0]].time_idx[-1] + blue_lines.lines[interaction_tuple[i][1]].time_idx[-1])/2)    
    if interaction_ends[i] == 2: # this means that blue left last, so the end of the colocalization is the last red timepoint
        coloc_end = red_lines.lines[interaction_tuple[i][0]].time_idx[-1]
        
    blue_red_colocalization_lifetimes.append((coloc_end-coloc_start) * dt)
    
print (blue_red_colocalization_lifetimes)
```

```
[3.6002304000000005, 5.00032, 3.8002432, 15.951020800000002, 9.800627200000001, 25.001600000000003, 29.351878400000004, 2.250144]
```

In [31]:

```
#Analysis of single-color events without colocalization only
        

def Filter(list1, list2):  #define a function to filter out any lines with interactions
    return (list(set(list2) - set(list1)))

red_extracted_list = []
for i in range (0, len(interaction_tuple)):
    red_extracted_list.append(interaction_tuple[i][0])
    
red_line_list = np.arange(0, numberofredlines, 1) #this generates a list of red lines that did not colocalize
solo_red_lines = Filter(red_extracted_list, red_line_list)


print (solo_red_lines)

solo_red_times_from_blue = []
for k in range (0, len(solo_red_lines)):
    solo_red_times_from_blue.append(redtimes[solo_red_lines[k]-1])
print (solo_red_times_from_blue)
print (len(red_line_list), len(solo_red_lines), len(solo_red_times_from_blue))

blue_extracted_list = []
for i in range (0, len(interaction_tuple)):
    blue_extracted_list.append(interaction_tuple[i][1])
    
blue_line_list = np.arange(0, numberofbluelines, 1) #this generates a list of blue lines that did not colocalize
solo_blue_lines = Filter(blue_extracted_list, blue_line_list)
#filteblue = (list(set(blue_line_list) - set(blue_extracted_list)))


solo_blue_times_from_red = []
for k in range (0, len(solo_blue_lines)):
    solo_blue_times_from_red.append(bluetimes[solo_blue_lines[k]-1])
print (solo_blue_times_from_red)
print (len(solo_blue_lines))
```

```
[0, 1, 2, 4, 6, 10, 13, 14, 15]
[102.20654080000001, 113.80728320000001, 19.001216000000003, 7.400473600000001, 9.000576, 19.401241600000002, 65.80421120000001, 63.80408320000001, 153.60983040000002]
16 9 9
[106.40680960000002, 22.001408, 158.21012480000002, 43.80280320000001, 7.0004480000000004, 243.015552, 68.4043776, 27.201740800000003, 31.402009600000003, 4.800307200000001]
10
```

In [32]:

```
#classic venn diagram showing how many blue alone, how many green alone, and how many colocalizations
#also lists unique interactions (i.e., how many lines of a color contribute to the middle portion)

plt.figure(figsize=(7, 4))
venn2(subsets = (len(solo_blue_lines), len(solo_red_lines), len(blue_red_colocalization_lifetimes)), set_labels = ('GFP-DDB1', 'JF-635-DDB2'), set_colors=('blue', 'red'), alpha = 0.5) 
plt.show()
print(len(redtimes)-len(solo_red_times_from_blue),"unique red interactions")
print(len(bluetimes)-len(solo_blue_times_from_red),"unique blue interactions")
print(len(redtimes))
```

```
7 unique red interactions
8 unique blue interactions
16
```

In [34]:

```
#outputs key parameters. Super useful for comparing multiple datasets.
#print (filename[0])

print (median_force)
print (redlifetime)
print (numberofredlines)
print (np.floor(max(time)), "s is the final timepoint")
print (dt)
print (pixel_size[0])
#print ('Localization precision is',np.average(standarddeviation)*1000, 'plus or minus', (sem(standarddeviation)*1000), 'nm, assuming no diffusion')

#this section saves down key parameters as .txt files. Can be used with the three-color analysis
np.savetxt("rednorm_meanpositions.txt", 
           rednorm_meanpositions,
           delimiter =", ",
            fmt = "%s")
np.savetxt("bluenorm_meanpositions.txt", 
           bluenorm_meanpositions,
           delimiter =", ",
            fmt = "%s")
np.savetxt("redtimes.txt", 
           (np.sort (redtimes)),
           delimiter =", ",
            fmt = "%s")
np.savetxt("bluetimes.txt", 
           (np.sort (bluetimes)),
           delimiter =", ",
            fmt = "%s")
np.savetxt("adjusted_redtimes.txt", 
           (np.sort (adjusted_redtimes)),
           delimiter =", ",
            fmt = "%s")
np.savetxt("adjusted_bluetimes.txt", 
           (np.sort (adjusted_bluetimes)),
           delimiter =", ",
            fmt = "%s")

np.savetxt("blue_red_colocalization_lifetimes.txt", 
           np.sort(blue_red_colocalization_lifetimes),
           delimiter =", ",
            fmt = "%s")
np.savetxt("solo_red_times_from_blue.txt", 
           (np.sort (solo_red_times_from_blue)),
           delimiter =", ",
            fmt = "%s")
np.savetxt("solo_blue_times_from_red.txt", 
           (np.sort (solo_blue_times_from_red)),
           delimiter =", ",
            fmt = "%s")

np.savetxt("red_blue_interaction_tuple.txt", 
           interaction_tuple,
           delimiter =", ",
            fmt = "%s")

xarray = np.array([1, 2, 3, 4, 5, 6, 7, 8, 9, 10, 11])
yarray = np.array([len(solo_blue_lines), len(solo_red_lines), interaction_categories.count(3), interaction_categories.count(4), interaction_categories.count(5), interaction_categories.count(6), interaction_categories.count(7), interaction_categories.count(8), interaction_categories.count(9), interaction_categories.count(10), interaction_categories.count(11)])

data = np.column_stack([xarray, yarray])

np.savetxt("categories_of_colocalization.txt", data, fmt=['%d','%d'])

#this section saves all of the .txt files to a new folder based on colors of analysis
#this way, multiple analyses of the data will not erase each other.
parent_dir = os.getcwd()
directory = "BlueandRed"
newpath = os.path.join(parent_dir, directory)
os.mkdir(newpath)
path = os.getcwd() 

inpath = path
outpath = (newpath)
file_names = os.listdir(parent_dir)
os.chdir(inpath)
for file in glob.glob("*.txt"):
    shutil.copy(inpath+'/'+file,outpath)
```

```
9.69581492265753
26.856840366118952
16
599.0 s is the final timepoint
0.10000640000000001
0.1
```

In [35]:

```
#rigorous way of showing order of assembly/dissassembly functions
plt.figure(figsize=(5, 3))
plt.bar(xarray, yarray)
plt.xlabel('Binding event category')
plt.ylabel('Times occuring')
plt.yscale("log")
plt.tight_layout()
plt.show()
```

In [ ]:

```

```
