# Supplementary material for: Single-molecule analysis of DNA-binding proteins from nuclear extracts (SMADNE)
Source: Nucleic Acids Res. 2023 Mar 2;51(7):e39. doi: 10.1093/nar/gkad095 (PMC10123111; doi:10.1093/nar/gkad095)
Supplement: gkad095_Supplemental_Files [file gkad095_supplemental_files.zip › C-trap_photobleach_analysis_V10-blue.html]

C-trap\_photobleach\_analysis\_V10-blue


In [1]:

```
#This notebook is intended to be used for photobleach calculations based on a kymograph collection of static fluorophores.

#To setup a collection, begin by performing continuous confocal scans while lowering the Z-position with the silver knob.
#When the Z focus is at the bottom edge of the slide, proteins stuck to the surface should fluoresce brightly.
#To analyze photobleaching, start collecting a kymograph as in data collection settings 
#until photon counts decrease to less than half of the original value.
#This script should automatically fetch and bin the photon counts in the blue channel by second and plot them as a function of time.
#Finally, an exponential decay fit determines the lifetime of the fluorophore (see figure 2)
#Note that color still must be changed in cells 5 and 7 for colors other than blue
```

In [2]:

```
import matplotlib.pyplot as plt
import lumicks.pylake as lk
import itertools
import ipywidgets
import numpy as np
import glob
import math
# We use skimage to downsample the data
from skimage.measure import block_reduce
from statistics import mean
from statistics import median
# Use notebook if you're in Jupyter Notebook
%matplotlib notebook
```

In [3]:

```
filename = glob.glob('*.h5') #fetches filename assuming only one .h5 in notebook
if len(filename) == 1:
    print (filename[0])
else: 
    print ("too many or too few .h5 files")
```

```
20210907-192448 Kymograph 27 5% photobleach with refocusing.h5
```

In [4]:

```
plt.close('all')
file = lk.File(str(filename[0]))
_, kymo = file.kymos.popitem()
```

In [10]:

```
forcex = file["Force HF"]["Force 1x"]

# time traces (seconds)
time = forcex.timestamps/1e9
time = time - time[0]
sample_rate = forcex.sample_rate

downsampled_rate = 600 # Hz
print (max(time), 'seconds is the length of the kymo') #displays the length of the kymo in seconds


kymo_time = np.floor(max(time))
# downsample the force, nanostage position and time
forcex_downsamp = forcex.downsampled_by(int(sample_rate/downsampled_rate))
time_downsamp = forcex_downsamp.timestamps/1e9
time_downsamp = time_downsamp - time_downsamp[0]

median_force = (median(forcex_downsamp.data))
#print (median(forcex_downsamp.data))
#print (mean(forcex_downsamp.data))

photons = file['Photon count']['Blue']
#print (len (photons)) for this dataset the number of timestamps in photon collection was 8017501
photonwindow = int(len(photons)/kymo_time) #divide the length of the dataset in seconds by the photon window to get photons/second

#1 second photons
photon_sum = 0
array_of_photon_sums = []
for k in range (1, 100):
    for i in range (k*photonwindow, (k+1)*photonwindow):  
        photon_sum = photon_sum + photons.data[i]
        if i == (k+1)*photonwindow-1:
            array_of_photon_sums.append(photon_sum)
            photon_sum = 0
```

```
286.8800001144409 seconds is the length of the kymo
```

In [11]:

```
_, kymo = file.kymos.popitem()
data = kymo.blue_image
downsample_factor = 60
data = block_reduce(data, (1, downsample_factor))
```

In [12]:

```
dt = downsample_factor * kymo.line_time_seconds
```

In [13]:

```
x = np.arange(1, 100) 
y = array_of_photon_sums
plt.figure(figsize=(6, 5))
plt.plot(x, y, color ="blue") 
plt.ylabel('Photon count')
plt.xlabel('Time [s]')
plt.title('Photon counts per second')
plt.tight_layout()
#plt.show()

from scipy.optimize import curve_fit
def func(x, a, b, c,d):

    return a * np.exp(-b * (x-d)) + c 
#NOTE: These bounds may need to be changed if the photon count is really high! Check if fit is near the bounds
popt, pcov = curve_fit(func, x, y, bounds=(0, [100000, 5, 50000, 5]), absolute_sigma=True)

plt.figure(figsize=(6,4))
plt.plot(x,y,'.')
plt.title('Photobleaching over time fit to exponential decay')
plt.ylabel('Photon count')
plt.xlabel('Time [s]')
plt.plot(x, func(x, *popt), 'r-',

         #label='fit: a=%5.3f, b=%5.3f, c=%5.3f, d=%5.3f' % tuple(popt)
        )
lifetime = 1 / popt[1]

plt.tight_layout()
#plt.legend()
plt.show()
residuals = y- func(x, *popt)
ss_res = np.sum(residuals**2)
ss_tot = np.sum((y-np.mean(y))**2)
r_squared = 1 - (ss_res / ss_tot)
print ('The R squared of the fit is', (r_squared))
print ('Mean bleach time is', (lifetime), 'seconds')
```

```
The R squared of the fit is 0.9876079636532984
Mean bleach time is 31.032849939471802 seconds
```
