# Supplementary material for: Single-molecule analysis of DNA-binding proteins from nuclear extracts (SMADNE)
Source: Nucleic Acids Res. 2023 Mar 2;51(7):e39. doi: 10.1093/nar/gkad095 (PMC10123111; doi:10.1093/nar/gkad095)
Supplement: gkad095_Supplemental_Files [file gkad095_supplemental_files.zip › SMADNE supplementary figures 12-21-22_fixed.pptx]

## Slide 1
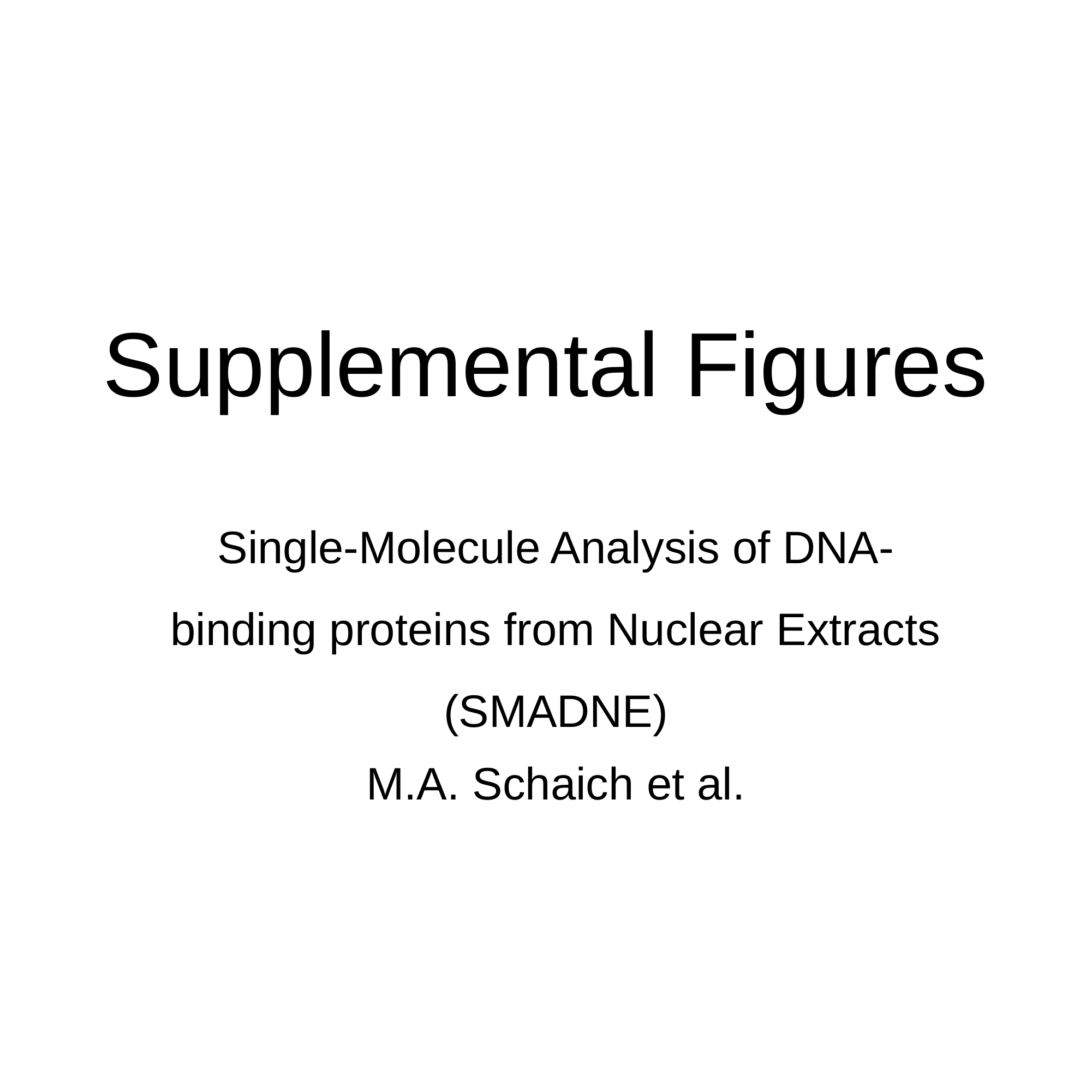

# Supplemental Figures
Single-Molecule Analysis of DNA-binding proteins from Nuclear Extracts (SMADNE)
M.A. Schaich et al.

## Slide 2
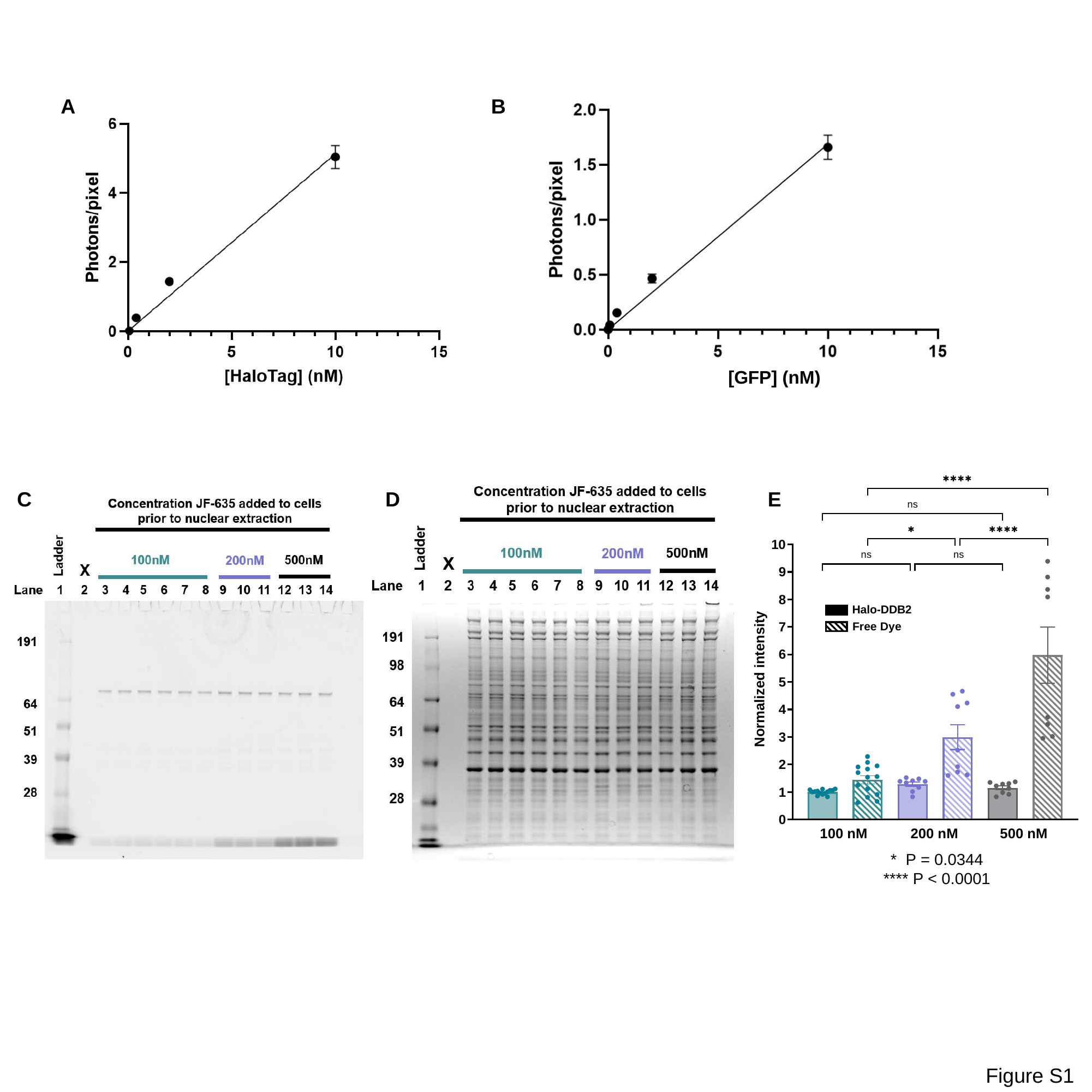

a
b
[GFP] (nM)
C
D
E
* P = 0.0344
**** P < 0.0001
Figure S1

## Slide 3
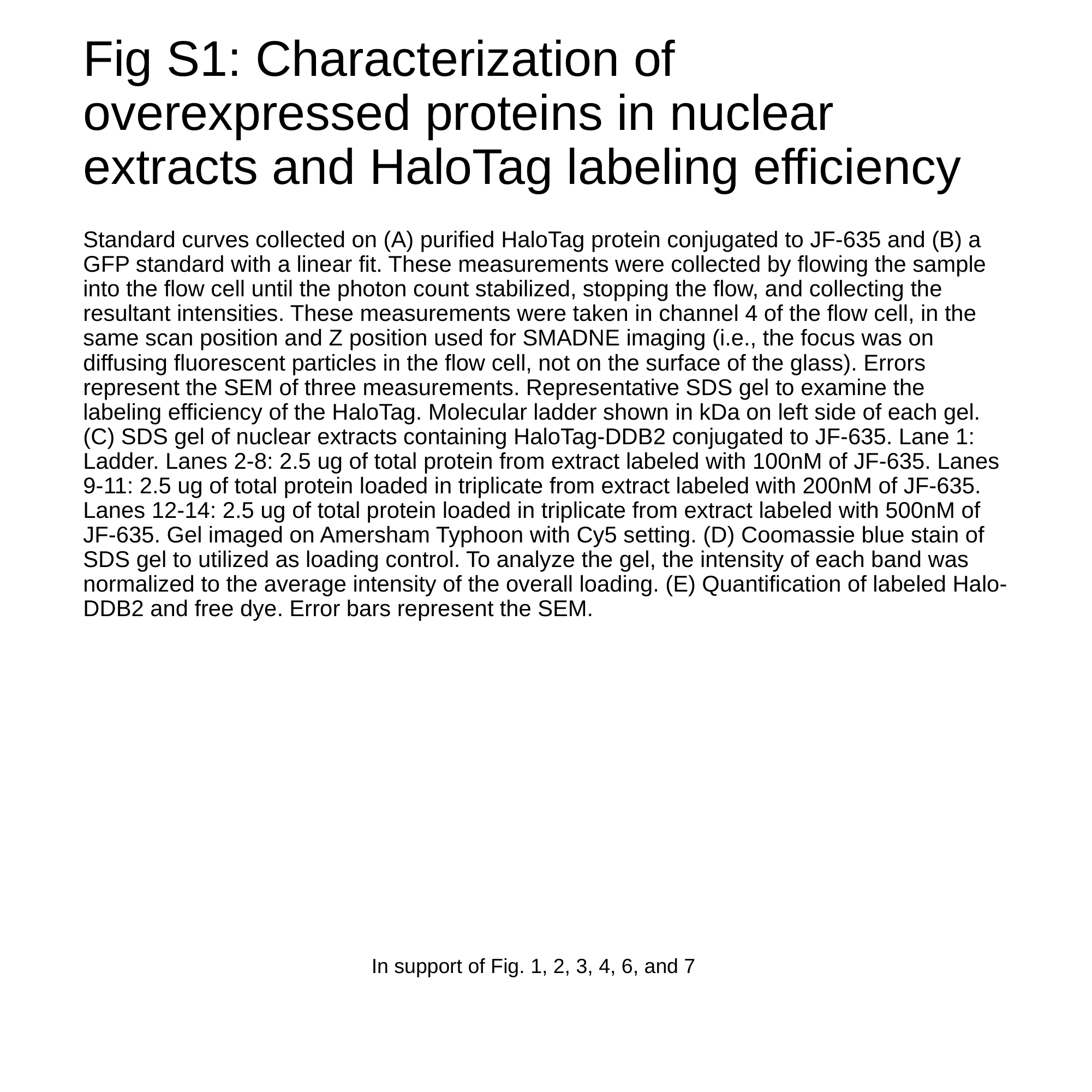

# Fig S1: Characterization of overexpressed proteins in nuclear extracts and HaloTag labeling efficiency
Standard curves collected on (A) purified HaloTag protein conjugated to JF-635 and (B) a GFP standard with a linear fit. These measurements were collected by flowing the sample into the flow cell until the photon count stabilized, stopping the flow, and collecting the resultant intensities. These measurements were taken in channel 4 of the flow cell, in the same scan position and Z position used for SMADNE imaging (i.e., the focus was on diffusing fluorescent particles in the flow cell, not on the surface of the glass). Errors represent the SEM of three measurements. Representative SDS gel to examine the labeling efficiency of the HaloTag. Molecular ladder shown in kDa on left side of each gel. (C) SDS gel of nuclear extracts containing HaloTag-DDB2 conjugated to JF-635. Lane 1: Ladder. Lanes 2-8: 2.5 ug of total protein from extract labeled with 100nM of JF-635. Lanes 9-11: 2.5 ug of total protein loaded in triplicate from extract labeled with 200nM of JF-635. Lanes 12-14: 2.5 ug of total protein loaded in triplicate from extract labeled with 500nM of JF-635. Gel imaged on Amersham Typhoon with Cy5 setting. (D) Coomassie blue stain of SDS gel to utilized as loading control. To analyze the gel, the intensity of each band was normalized to the average intensity of the overall loading. (E) Quantification of labeled Halo-DDB2 and free dye. Error bars represent the SEM.
In support of Fig. 1, 2, 3, 4, 6, and 7

## Slide 4
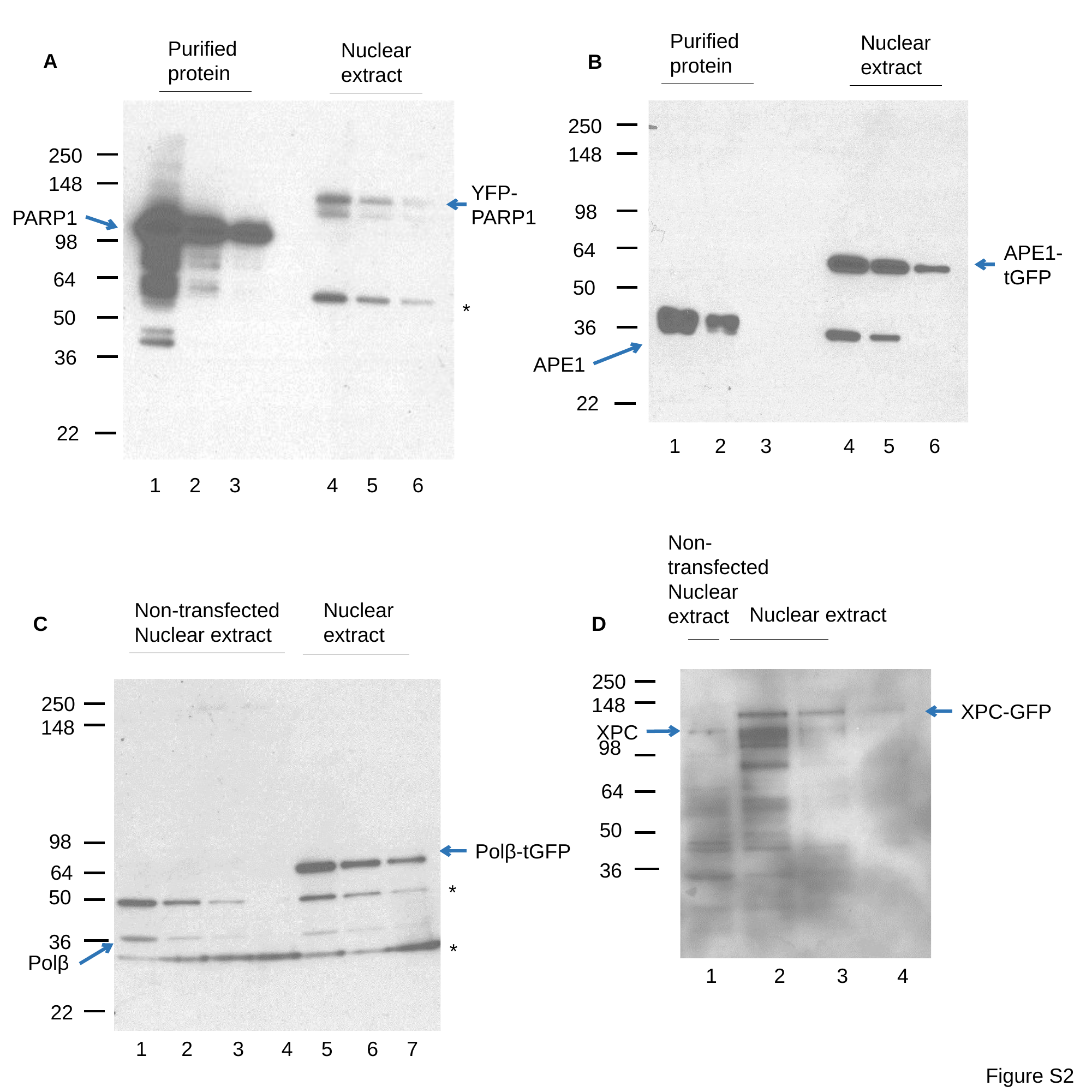

Purified
protein
Nuclear
extract
Purified
protein
Nuclear
extract
A
B
250
148
250
148
YFP-
PARP1
98
PARP1
98
64
APE1-tGFP
64
50
*
50
36
36
APE1
22
22
1 2 3 4 5 6
1 2 3 4 5 6
Non-transfected
Nuclear extract
Non-transfected
Nuclear extract
Nuclear
extract
Nuclear extract
D
C
250
250
148
XPC-GFP
148
XPC
98
64
50
98
Polβ-tGFP
36
64
*
50
36
*
Polβ
1 2 3 4
22
1 2 3 4 5 6 7
Figure S2

## Slide 5
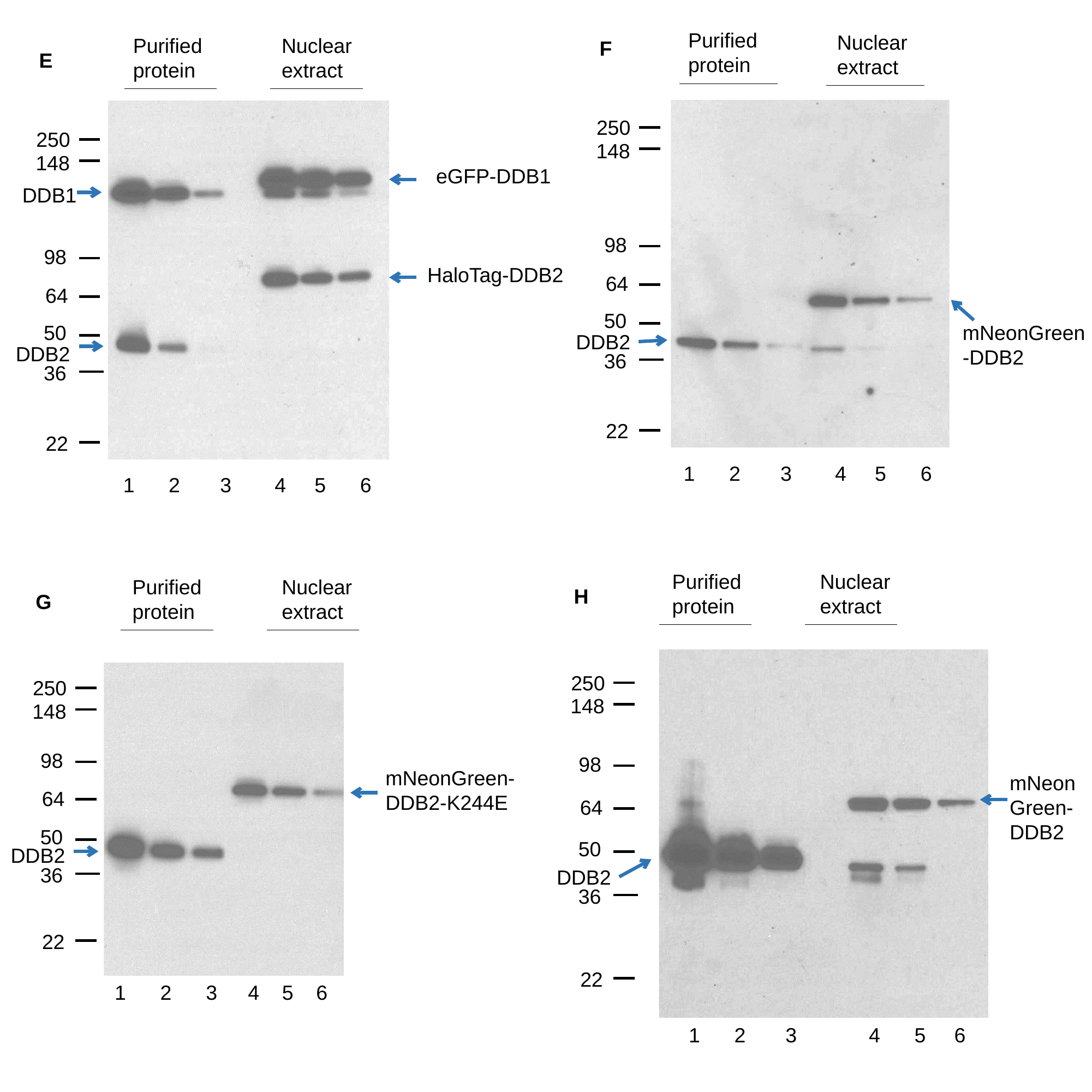

Purified
protein
Nuclear
extract
Purified
protein
Nuclear
extract
F
E
250
250
148
148
eGFP-DDB1
DDB1
98
98
HaloTag-DDB2
64
64
50
50
mNeonGreen
-DDB2
DDB2
DDB2
36
36
22
22
1 2 3 4 5 6
1 2 3 4 5 6
Purified
protein
Nuclear
extract
Purified
protein
Nuclear
extract
H
G
250
250
148
148
98
98
mNeonGreen-DDB2-K244E
mNeonGreen-DDB2
64
64
50
50
DDB2
36
DDB2
36
22
22
1 2 3 4 5 6
1 2 3 4 5 6

## Slide 6
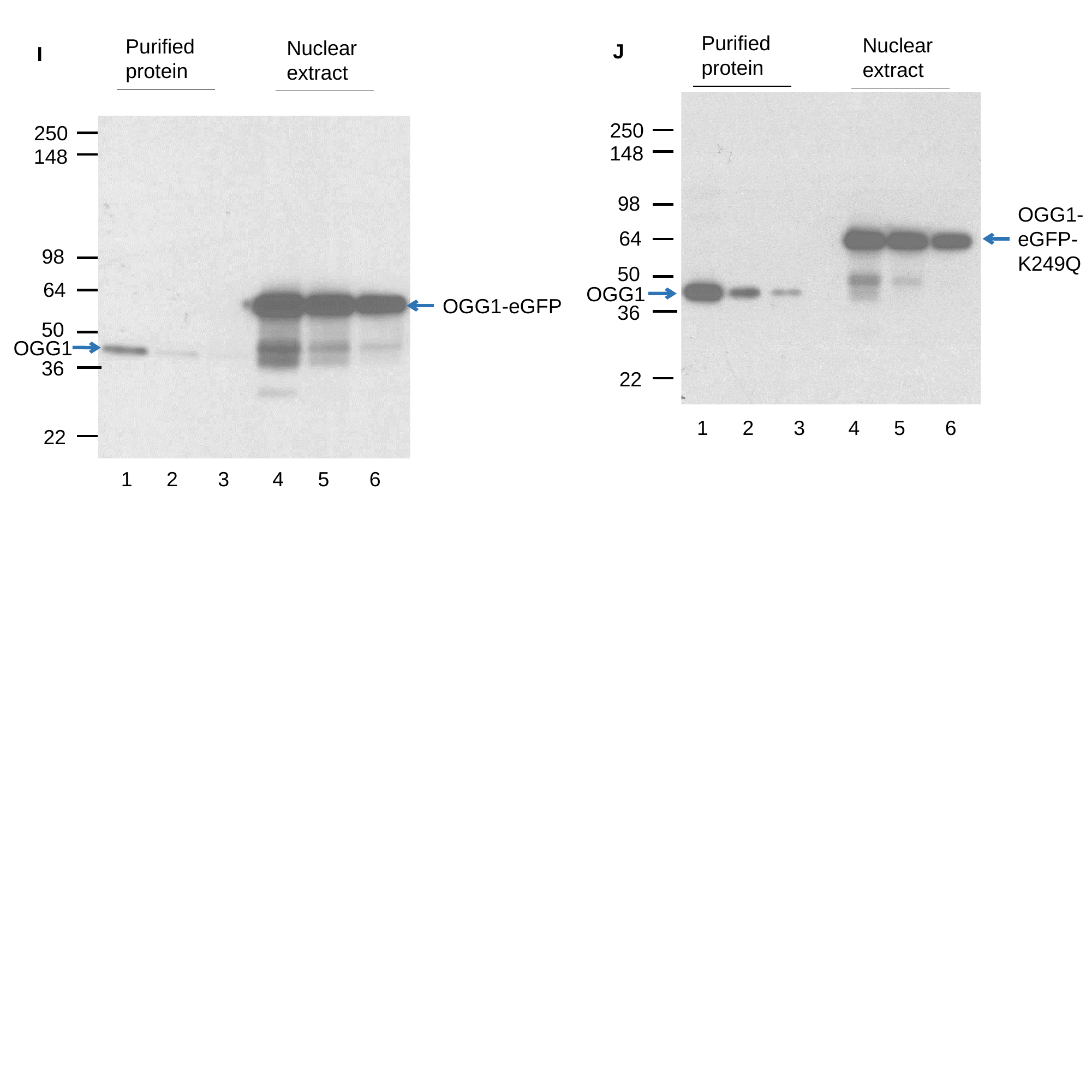

Purified
protein
Nuclear
extract
Purified
protein
Nuclear
extract
J
I
250
250
148
148
98
OGG1-eGFP-K249Q
64
98
50
64
OGG1
OGG1-eGFP
36
50
OGG1
36
22
1 2 3 4 5 6
22
1 2 3 4 5 6

## Slide 7
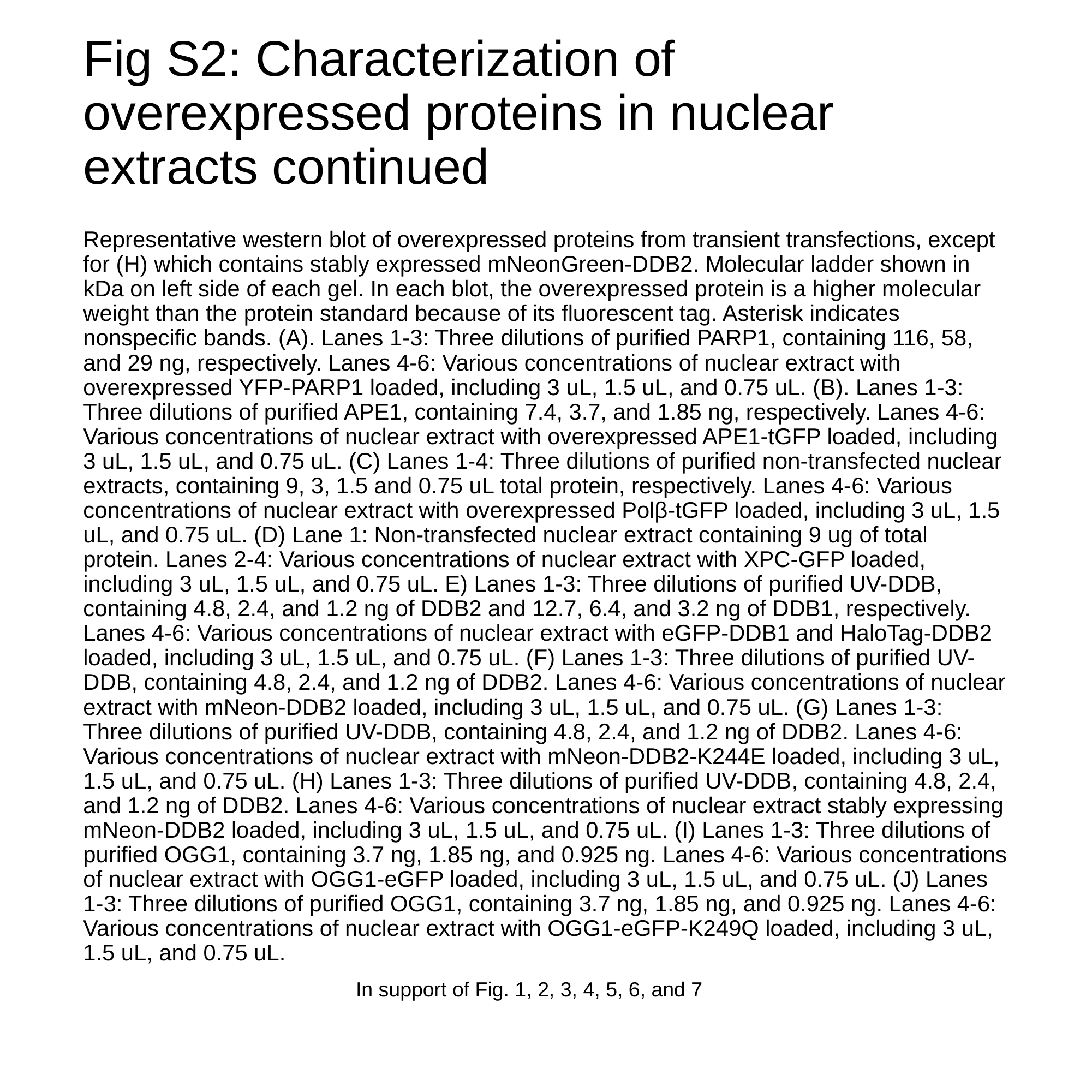

# Fig S2: Characterization of overexpressed proteins in nuclear extracts continued
Representative western blot of overexpressed proteins from transient transfections, except for (H) which contains stably expressed mNeonGreen-DDB2. Molecular ladder shown in kDa on left side of each gel. In each blot, the overexpressed protein is a higher molecular weight than the protein standard because of its fluorescent tag. Asterisk indicates nonspecific bands. (A). Lanes 1-3: Three dilutions of purified PARP1, containing 116, 58, and 29 ng, respectively. Lanes 4-6: Various concentrations of nuclear extract with overexpressed YFP-PARP1 loaded, including 3 uL, 1.5 uL, and 0.75 uL. (B). Lanes 1-3: Three dilutions of purified APE1, containing 7.4, 3.7, and 1.85 ng, respectively. Lanes 4-6: Various concentrations of nuclear extract with overexpressed APE1-tGFP loaded, including 3 uL, 1.5 uL, and 0.75 uL. (C) Lanes 1-4: Three dilutions of purified non-transfected nuclear extracts, containing 9, 3, 1.5 and 0.75 uL total protein, respectively. Lanes 4-6: Various concentrations of nuclear extract with overexpressed Polβ-tGFP loaded, including 3 uL, 1.5 uL, and 0.75 uL. (D) Lane 1: Non-transfected nuclear extract containing 9 ug of total protein. Lanes 2-4: Various concentrations of nuclear extract with XPC-GFP loaded, including 3 uL, 1.5 uL, and 0.75 uL. E) Lanes 1-3: Three dilutions of purified UV-DDB, containing 4.8, 2.4, and 1.2 ng of DDB2 and 12.7, 6.4, and 3.2 ng of DDB1, respectively. Lanes 4-6: Various concentrations of nuclear extract with eGFP-DDB1 and HaloTag-DDB2 loaded, including 3 uL, 1.5 uL, and 0.75 uL. (F) Lanes 1-3: Three dilutions of purified UV-DDB, containing 4.8, 2.4, and 1.2 ng of DDB2. Lanes 4-6: Various concentrations of nuclear extract with mNeon-DDB2 loaded, including 3 uL, 1.5 uL, and 0.75 uL. (G) Lanes 1-3: Three dilutions of purified UV-DDB, containing 4.8, 2.4, and 1.2 ng of DDB2. Lanes 4-6: Various concentrations of nuclear extract with mNeon-DDB2-K244E loaded, including 3 uL, 1.5 uL, and 0.75 uL. (H) Lanes 1-3: Three dilutions of purified UV-DDB, containing 4.8, 2.4, and 1.2 ng of DDB2. Lanes 4-6: Various concentrations of nuclear extract stably expressing mNeon-DDB2 loaded, including 3 uL, 1.5 uL, and 0.75 uL. (I) Lanes 1-3: Three dilutions of purified OGG1, containing 3.7 ng, 1.85 ng, and 0.925 ng. Lanes 4-6: Various concentrations of nuclear extract with OGG1-eGFP loaded, including 3 uL, 1.5 uL, and 0.75 uL. (J) Lanes 1-3: Three dilutions of purified OGG1, containing 3.7 ng, 1.85 ng, and 0.925 ng. Lanes 4-6: Various concentrations of nuclear extract with OGG1-eGFP-K249Q loaded, including 3 uL, 1.5 uL, and 0.75 uL.
In support of Fig. 1, 2, 3, 4, 5, 6, and 7

## Slide 8
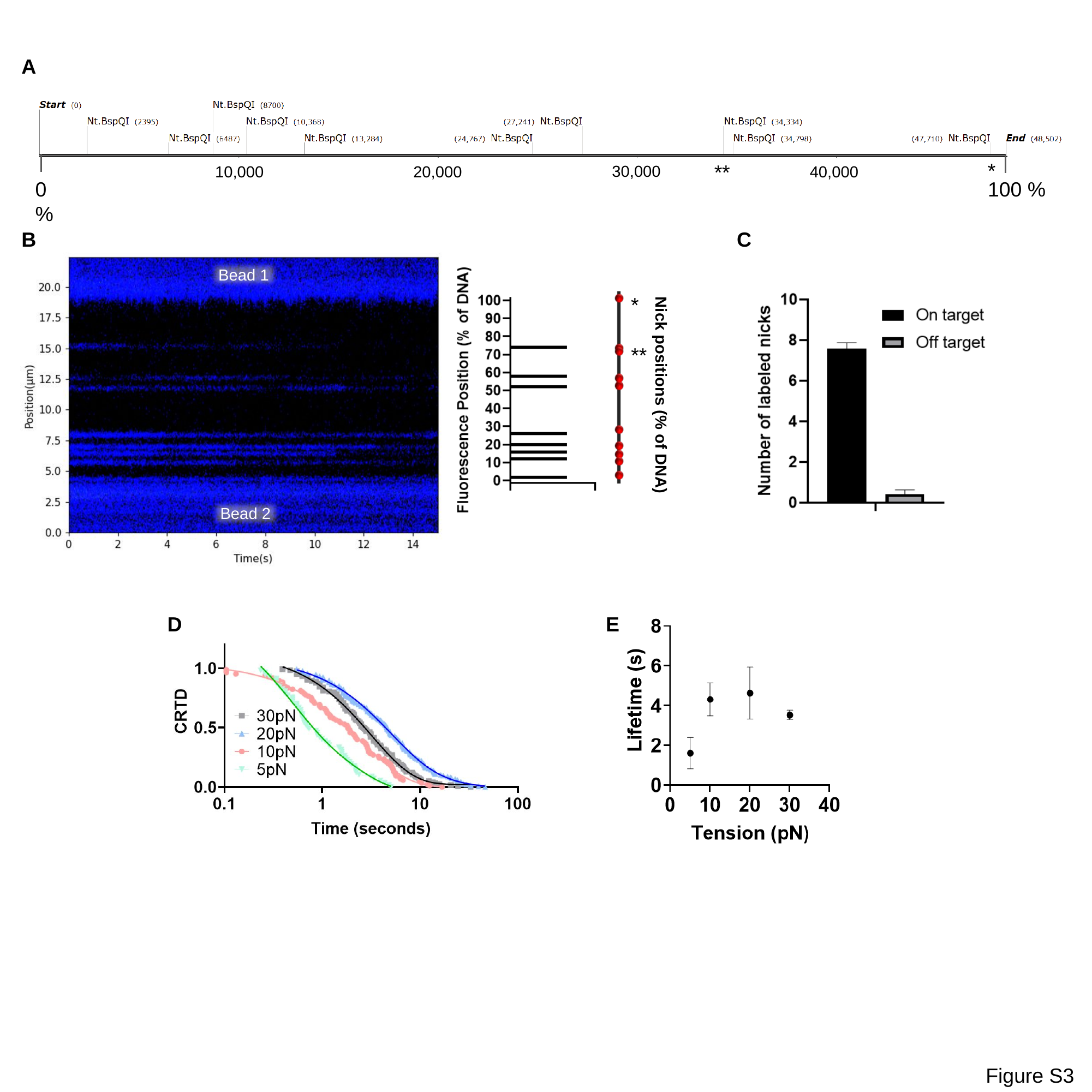

a
*
**
30,000
10,000
20,000
40,000
0 %
100 %
b
c
Bead 1
*
**
Nick positions (% of DNA)
Bead 2
d
e
Figure S3

## Slide 9
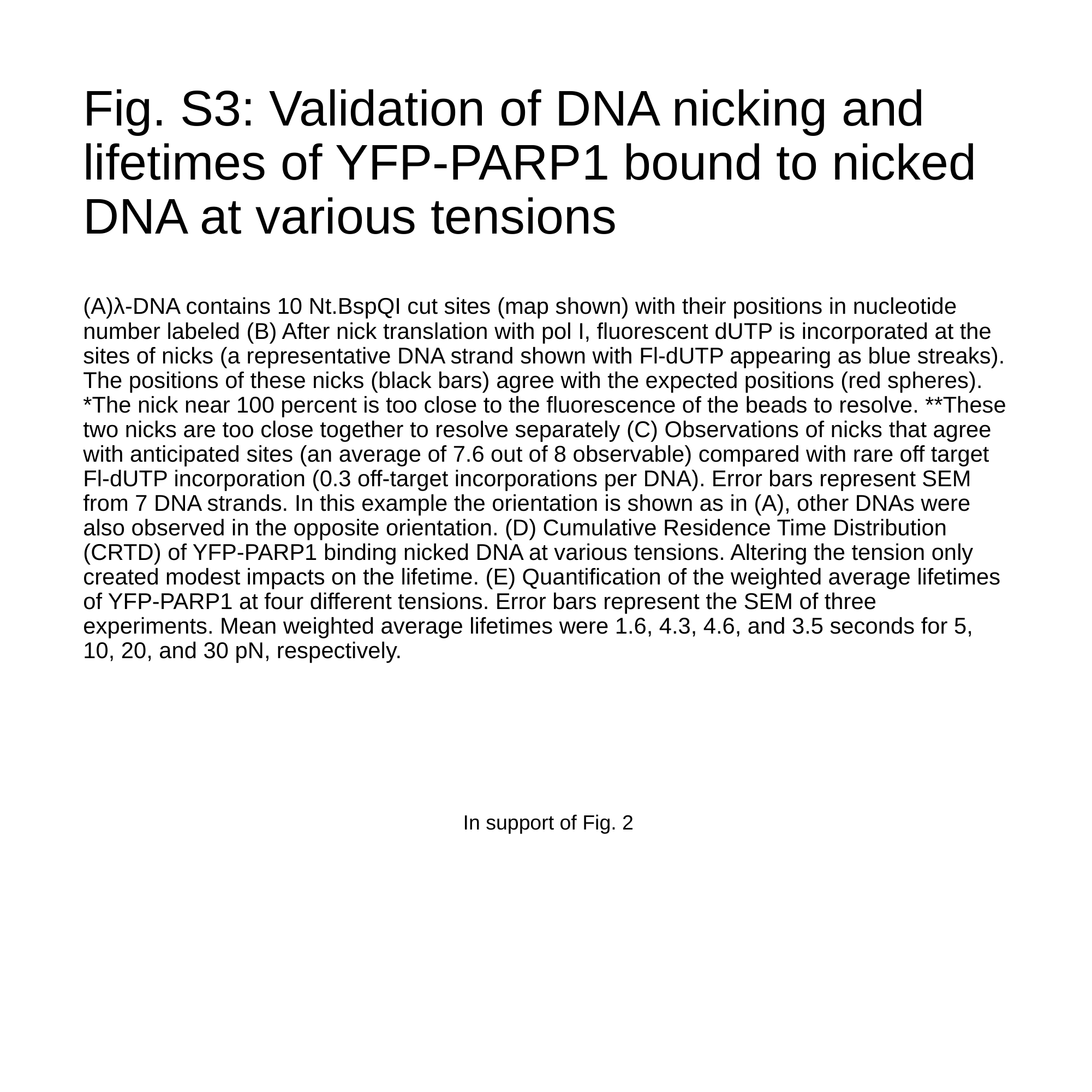

# Fig. S3: Validation of DNA nicking and lifetimes of YFP-PARP1 bound to nicked DNA at various tensions
(A)λ-DNA contains 10 Nt.BspQI cut sites (map shown) with their positions in nucleotide number labeled (B) After nick translation with pol I, fluorescent dUTP is incorporated at the sites of nicks (a representative DNA strand shown with Fl-dUTP appearing as blue streaks). The positions of these nicks (black bars) agree with the expected positions (red spheres). *The nick near 100 percent is too close to the fluorescence of the beads to resolve. **These two nicks are too close together to resolve separately (C) Observations of nicks that agree with anticipated sites (an average of 7.6 out of 8 observable) compared with rare off target Fl-dUTP incorporation (0.3 off-target incorporations per DNA). Error bars represent SEM from 7 DNA strands. In this example the orientation is shown as in (A), other DNAs were also observed in the opposite orientation. (D) Cumulative Residence Time Distribution (CRTD) of YFP-PARP1 binding nicked DNA at various tensions. Altering the tension only created modest impacts on the lifetime. (E) Quantification of the weighted average lifetimes of YFP-PARP1 at four different tensions. Error bars represent the SEM of three experiments. Mean weighted average lifetimes were 1.6, 4.3, 4.6, and 3.5 seconds for 5, 10, 20, and 30 pN, respectively.
In support of Fig. 2

## Slide 10
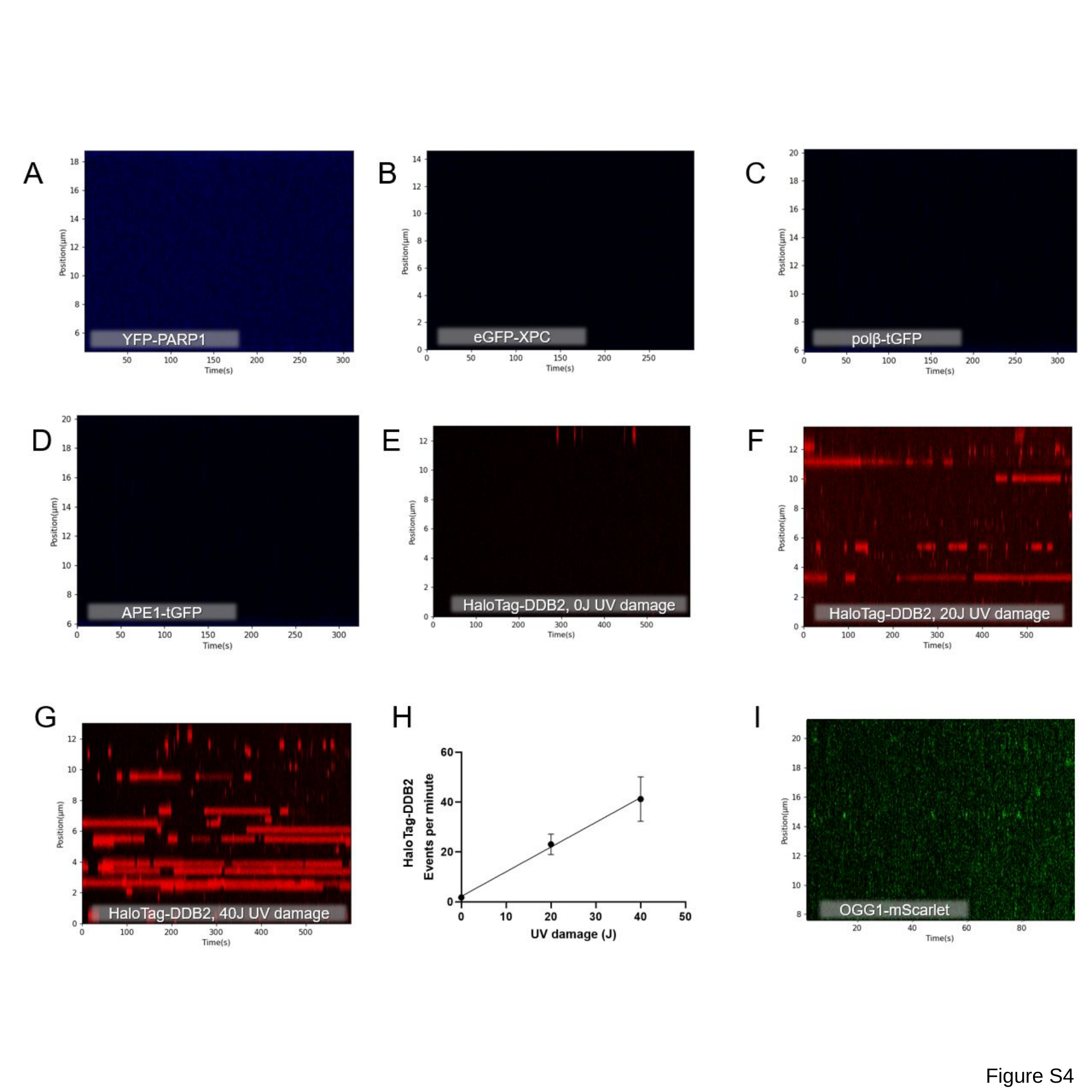

Figure S4

## Slide 11
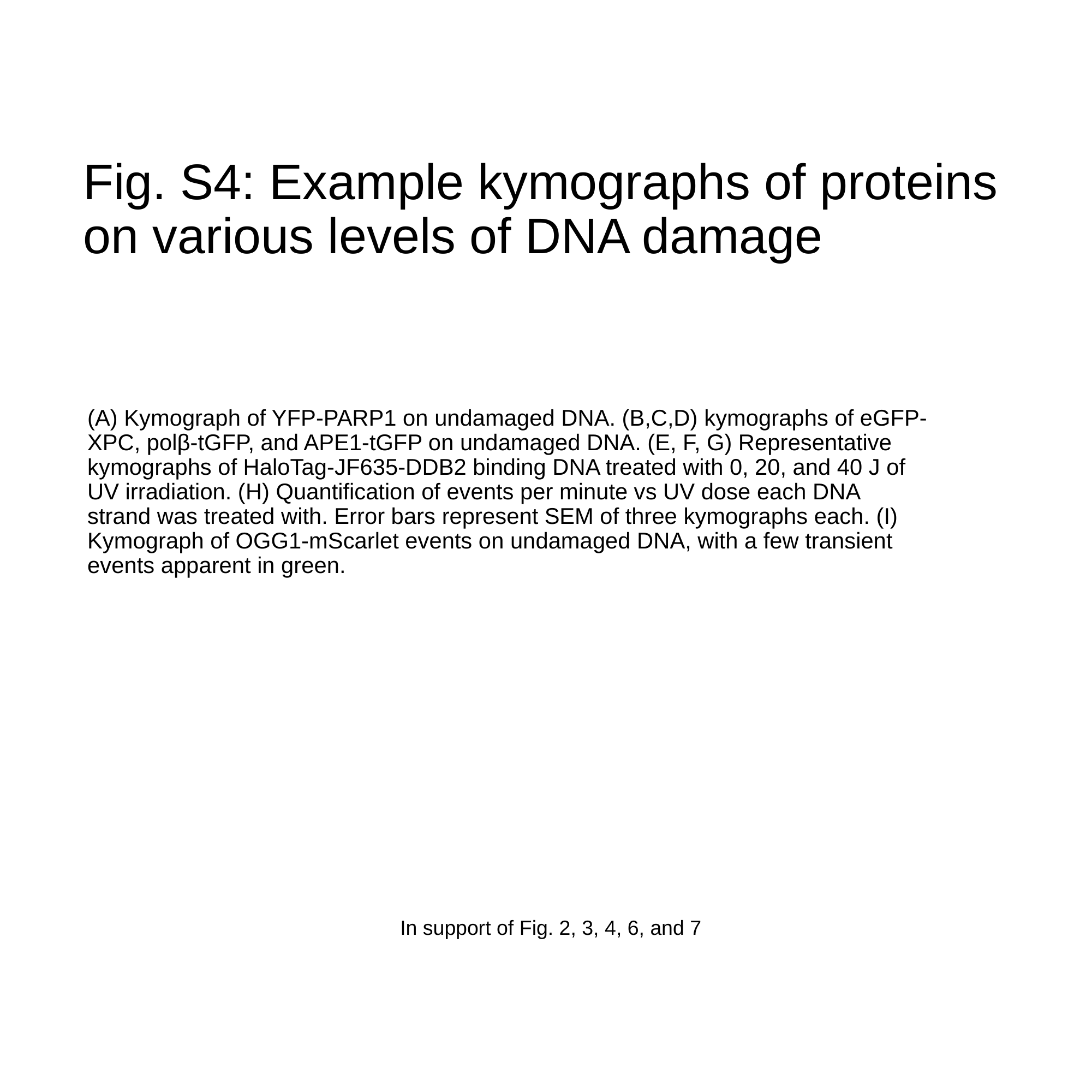

# Fig. S4: Example kymographs of proteins on various levels of DNA damage
(A) Kymograph of YFP-PARP1 on undamaged DNA. (B,C,D) kymographs of eGFP-XPC, polβ-tGFP, and APE1-tGFP on undamaged DNA. (E, F, G) Representative kymographs of HaloTag-JF635-DDB2 binding DNA treated with 0, 20, and 40 J of UV irradiation. (H) Quantification of events per minute vs UV dose each DNA strand was treated with. Error bars represent SEM of three kymographs each. (I) Kymograph of OGG1-mScarlet events on undamaged DNA, with a few transient events apparent in green.
In support of Fig. 2, 3, 4, 6, and 7

## Slide 12
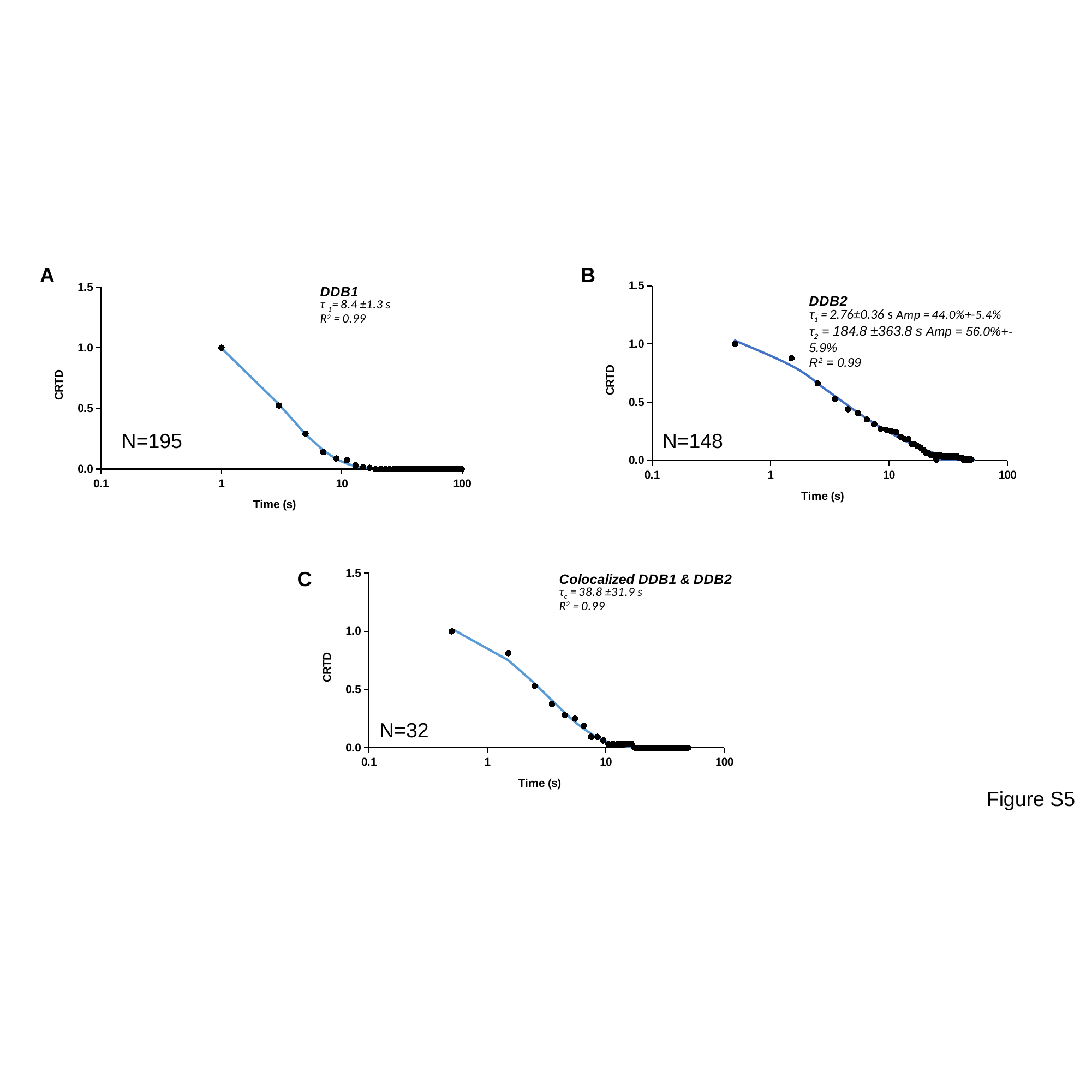

a
b
### Chart
| Category | CRTD | Double exp |
|---|---|---|
### Chart:
| Category | CRTD | Single exp |
|---|---|---|N=195
N=148
c
### Chart:
| Category | | Single exp |
|---|---|---|N=32
Figure S5

## Slide 13
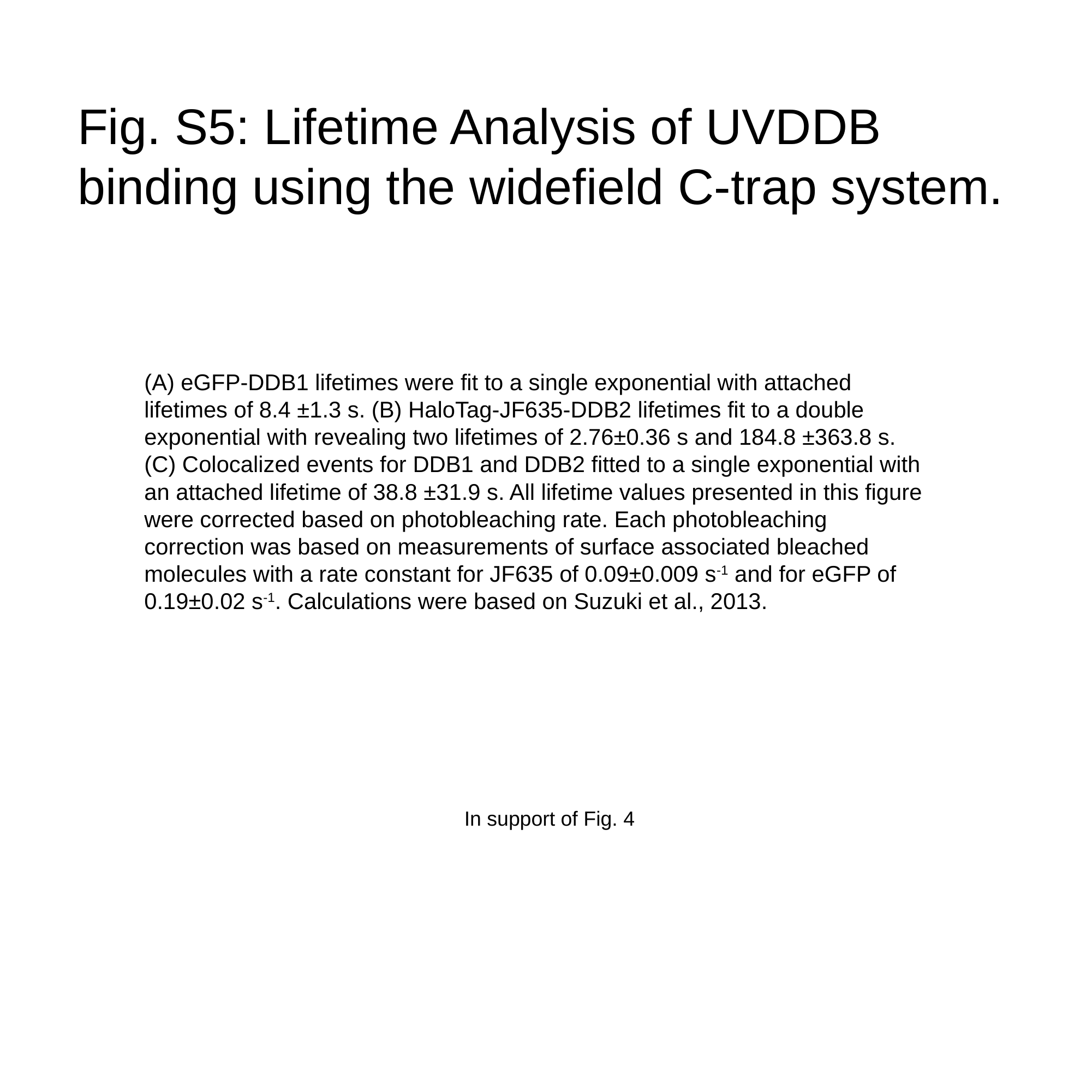

Fig. S5: Lifetime Analysis of UVDDB binding using the widefield C-trap system.
(A) eGFP-DDB1 lifetimes were fit to a single exponential with attached lifetimes of 8.4 ±1.3 s. (B) HaloTag-JF635-DDB2 lifetimes fit to a double exponential with revealing two lifetimes of 2.76±0.36 s and 184.8 ±363.8 s. (C) Colocalized events for DDB1 and DDB2 fitted to a single exponential with an attached lifetime of 38.8 ±31.9 s. All lifetime values presented in this figure were corrected based on photobleaching rate. Each photobleaching correction was based on measurements of surface associated bleached molecules with a rate constant for JF635 of 0.09±0.009 s-1 and for eGFP of 0.19±0.02 s-1. Calculations were based on Suzuki et al., 2013.
In support of Fig. 4

## Slide 14
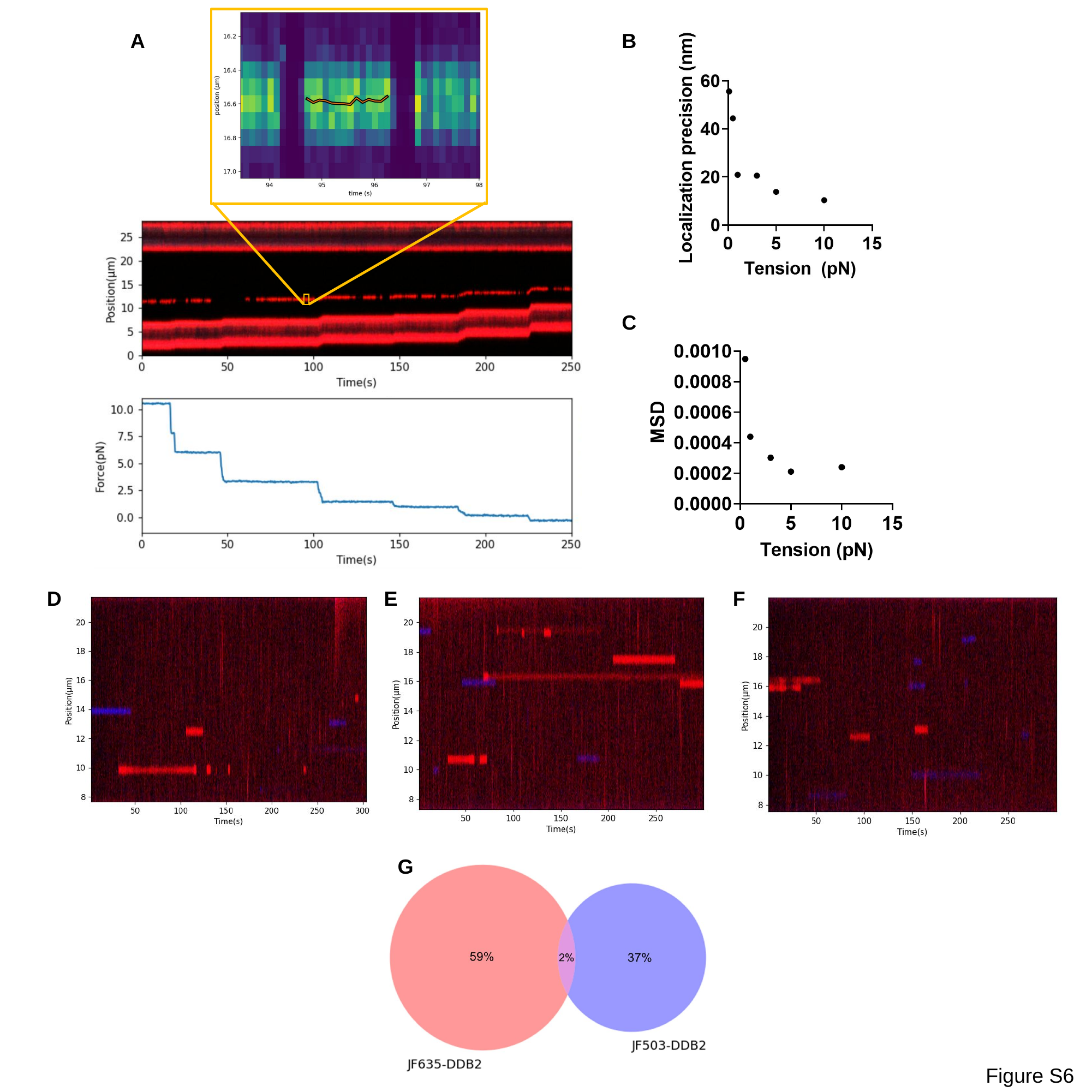

a
b
c
f
d
e
g
Figure S6

## Slide 15
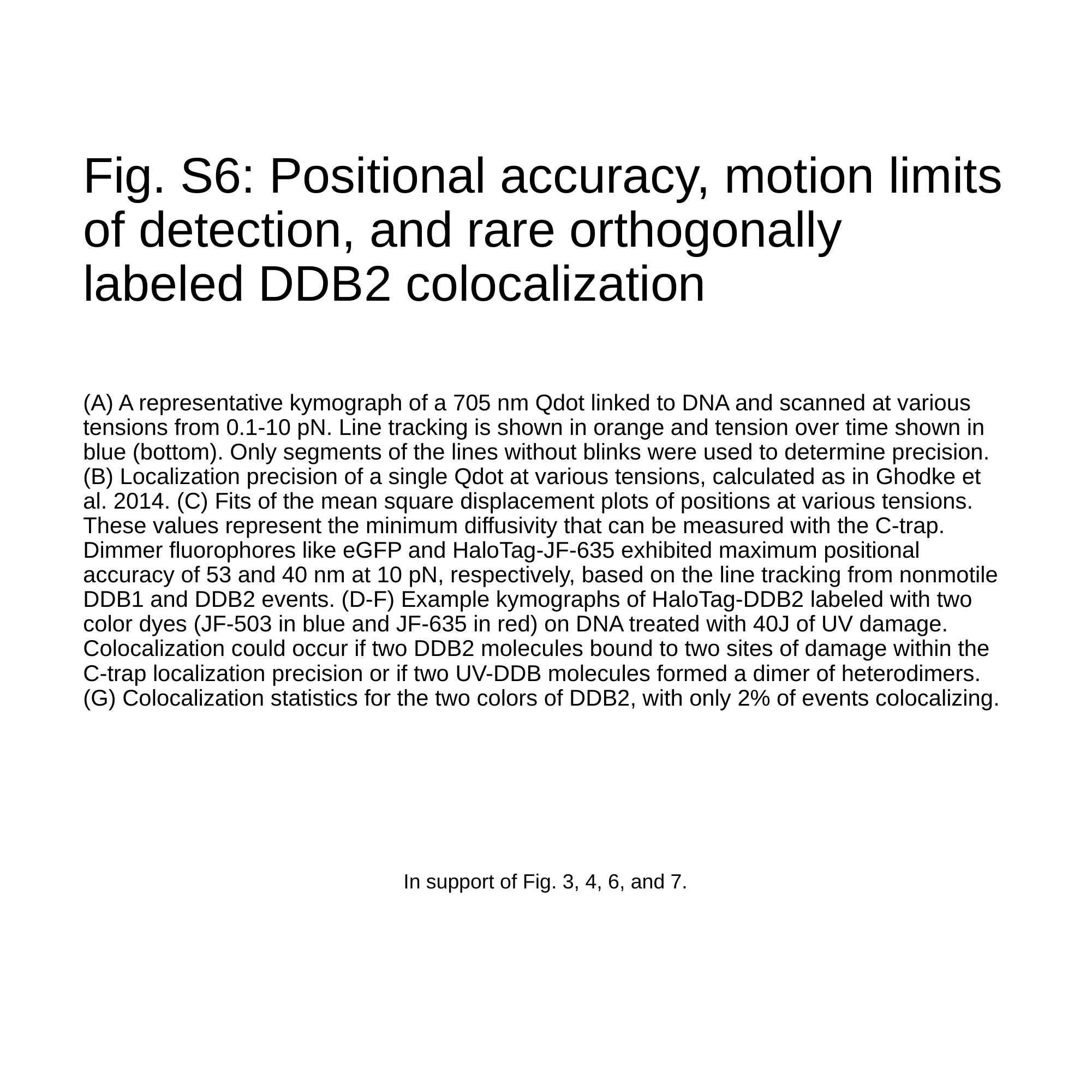

# Fig. S6: Positional accuracy, motion limits of detection, and rare orthogonally labeled DDB2 colocalization
(A) A representative kymograph of a 705 nm Qdot linked to DNA and scanned at various tensions from 0.1-10 pN. Line tracking is shown in orange and tension over time shown in blue (bottom). Only segments of the lines without blinks were used to determine precision. (B) Localization precision of a single Qdot at various tensions, calculated as in Ghodke et al. 2014. (C) Fits of the mean square displacement plots of positions at various tensions. These values represent the minimum diffusivity that can be measured with the C-trap. Dimmer fluorophores like eGFP and HaloTag-JF-635 exhibited maximum positional accuracy of 53 and 40 nm at 10 pN, respectively, based on the line tracking from nonmotile DDB1 and DDB2 events. (D-F) Example kymographs of HaloTag-DDB2 labeled with two color dyes (JF-503 in blue and JF-635 in red) on DNA treated with 40J of UV damage. Colocalization could occur if two DDB2 molecules bound to two sites of damage within the C-trap localization precision or if two UV-DDB molecules formed a dimer of heterodimers. (G) Colocalization statistics for the two colors of DDB2, with only 2% of events colocalizing.
In support of Fig. 3, 4, 6, and 7.

## Slide 16
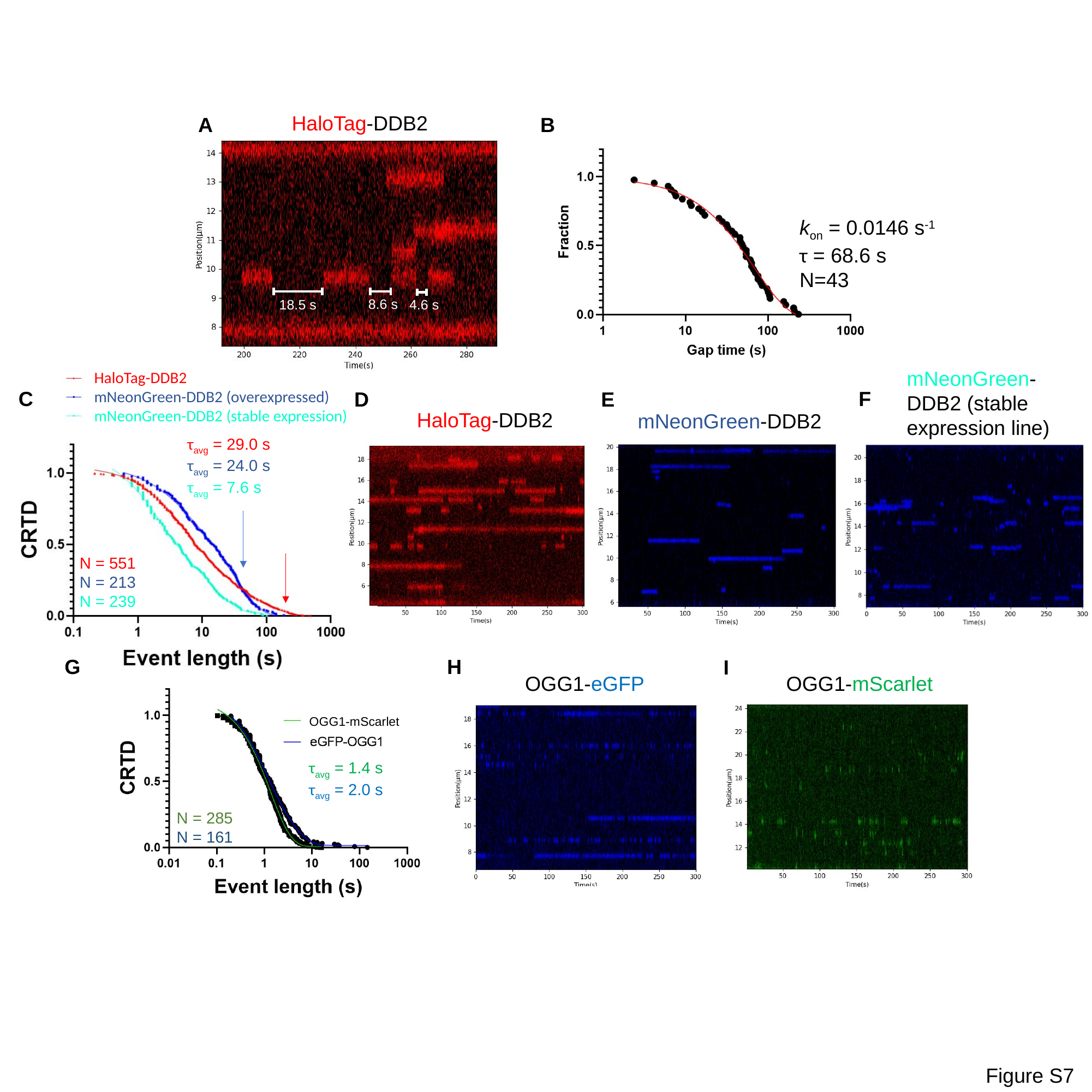

HaloTag-DDB2
A
B
kon = 0.0146 s-1
τ = 68.6 s
N=43
8.6 s
4.6 s
18.5 s
mNeonGreen-DDB2 (stable expression line)
HaloTag-DDB2
mNeonGreen-DDB2 (overexpressed)
mNeonGreen-DDB2 (stable expression)
F
C
D
E
HaloTag-DDB2
mNeonGreen-DDB2
τavg = 29.0 s
τavg = 24.0 s
τavg = 7.6 s
N = 551
N = 213
N = 239
G
H
I
OGG1-mScarlet
OGG1-eGFP
OGG1-mScarlet
τavg = 1.4 s
τavg = 2.0 s
N = 285
N = 161
Figure S7

## Slide 17
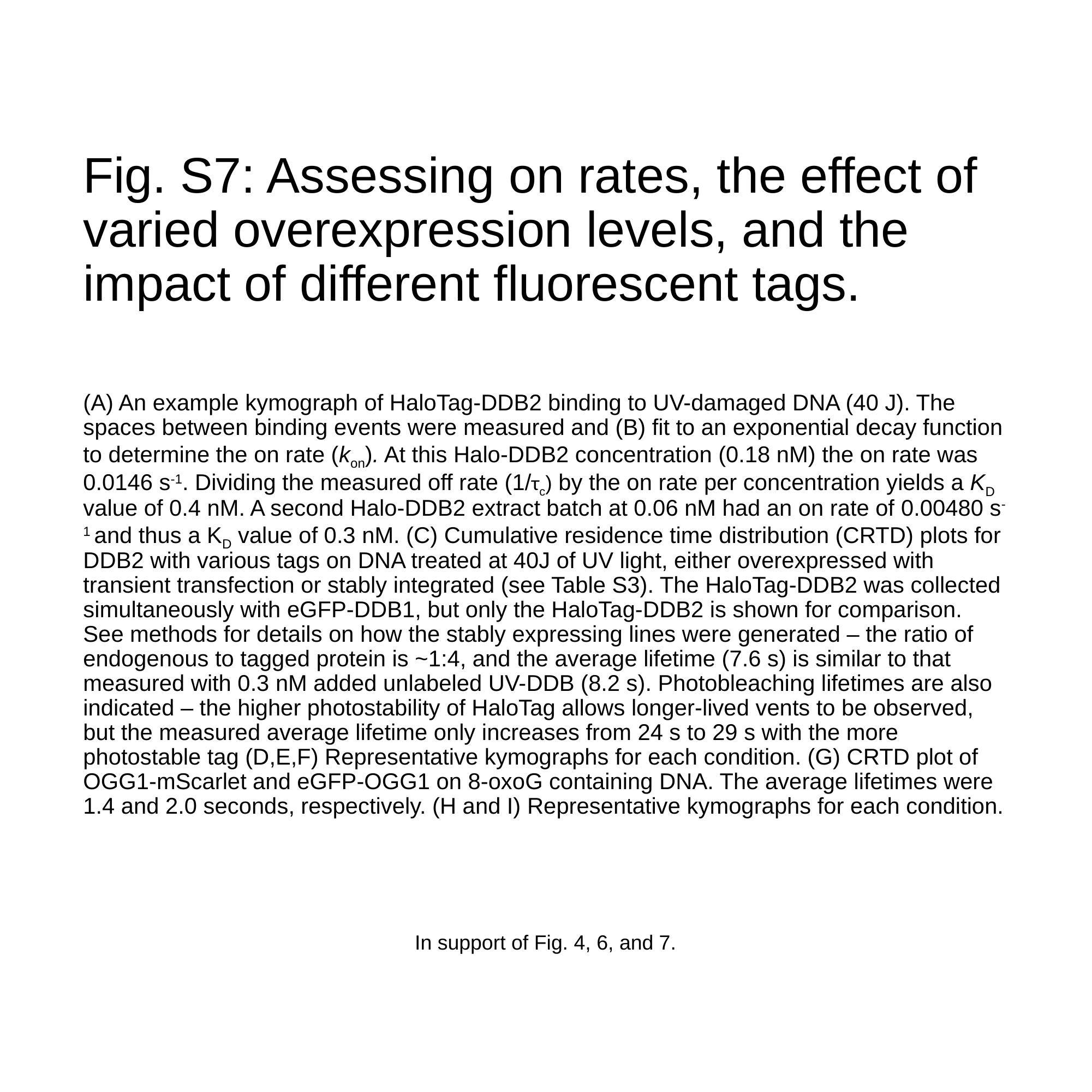

# Fig. S7: Assessing on rates, the effect of varied overexpression levels, and the impact of different fluorescent tags.
(A) An example kymograph of HaloTag-DDB2 binding to UV-damaged DNA (40 J). The spaces between binding events were measured and (B) fit to an exponential decay function to determine the on rate (kon). At this Halo-DDB2 concentration (0.18 nM) the on rate was 0.0146 s-1. Dividing the measured off rate (1/τc) by the on rate per concentration yields a KD value of 0.4 nM. A second Halo-DDB2 extract batch at 0.06 nM had an on rate of 0.00480 s-1 and thus a KD value of 0.3 nM. (C) Cumulative residence time distribution (CRTD) plots for DDB2 with various tags on DNA treated at 40J of UV light, either overexpressed with transient transfection or stably integrated (see Table S3). The HaloTag-DDB2 was collected simultaneously with eGFP-DDB1, but only the HaloTag-DDB2 is shown for comparison. See methods for details on how the stably expressing lines were generated – the ratio of endogenous to tagged protein is ~1:4, and the average lifetime (7.6 s) is similar to that measured with 0.3 nM added unlabeled UV-DDB (8.2 s). Photobleaching lifetimes are also indicated – the higher photostability of HaloTag allows longer-lived vents to be observed, but the measured average lifetime only increases from 24 s to 29 s with the more photostable tag (D,E,F) Representative kymographs for each condition. (G) CRTD plot of OGG1-mScarlet and eGFP-OGG1 on 8-oxoG containing DNA. The average lifetimes were 1.4 and 2.0 seconds, respectively. (H and I) Representative kymographs for each condition.
In support of Fig. 4, 6, and 7.

## Slide 18
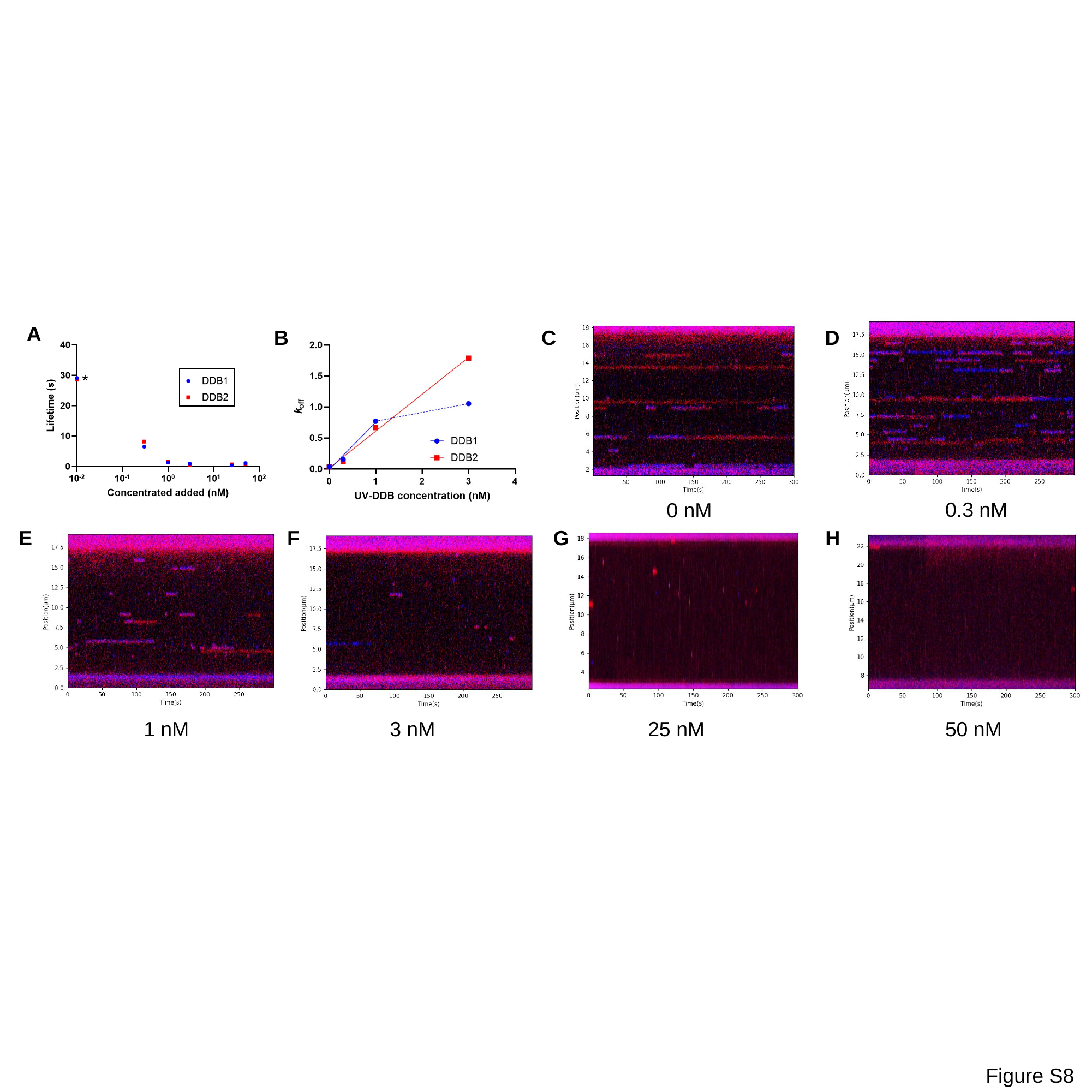

a
b
c
d
*
0.3 nM
0 nM
e
f
g
h
1 nM
3 nM
25 nM
50 nM
Figure S8

## Slide 19
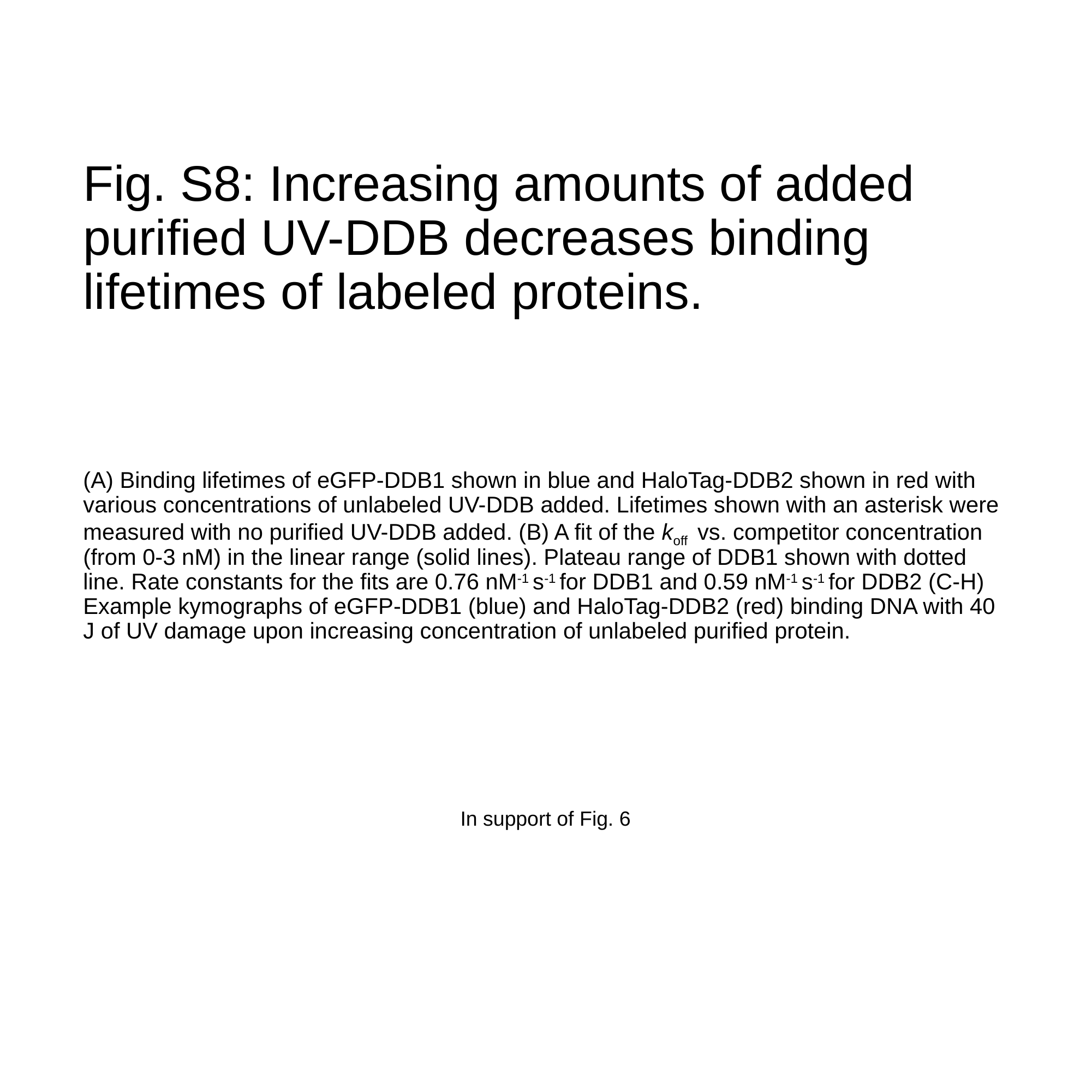

# Fig. S8: Increasing amounts of added purified UV-DDB decreases binding lifetimes of labeled proteins.
(A) Binding lifetimes of eGFP-DDB1 shown in blue and HaloTag-DDB2 shown in red with various concentrations of unlabeled UV-DDB added. Lifetimes shown with an asterisk were measured with no purified UV-DDB added. (B) A fit of the koff vs. competitor concentration (from 0-3 nM) in the linear range (solid lines). Plateau range of DDB1 shown with dotted line. Rate constants for the fits are 0.76 nM-1 s-1 for DDB1 and 0.59 nM-1 s-1 for DDB2 (C-H) Example kymographs of eGFP-DDB1 (blue) and HaloTag-DDB2 (red) binding DNA with 40 J of UV damage upon increasing concentration of unlabeled purified protein.
In support of Fig. 6

## Slide 20
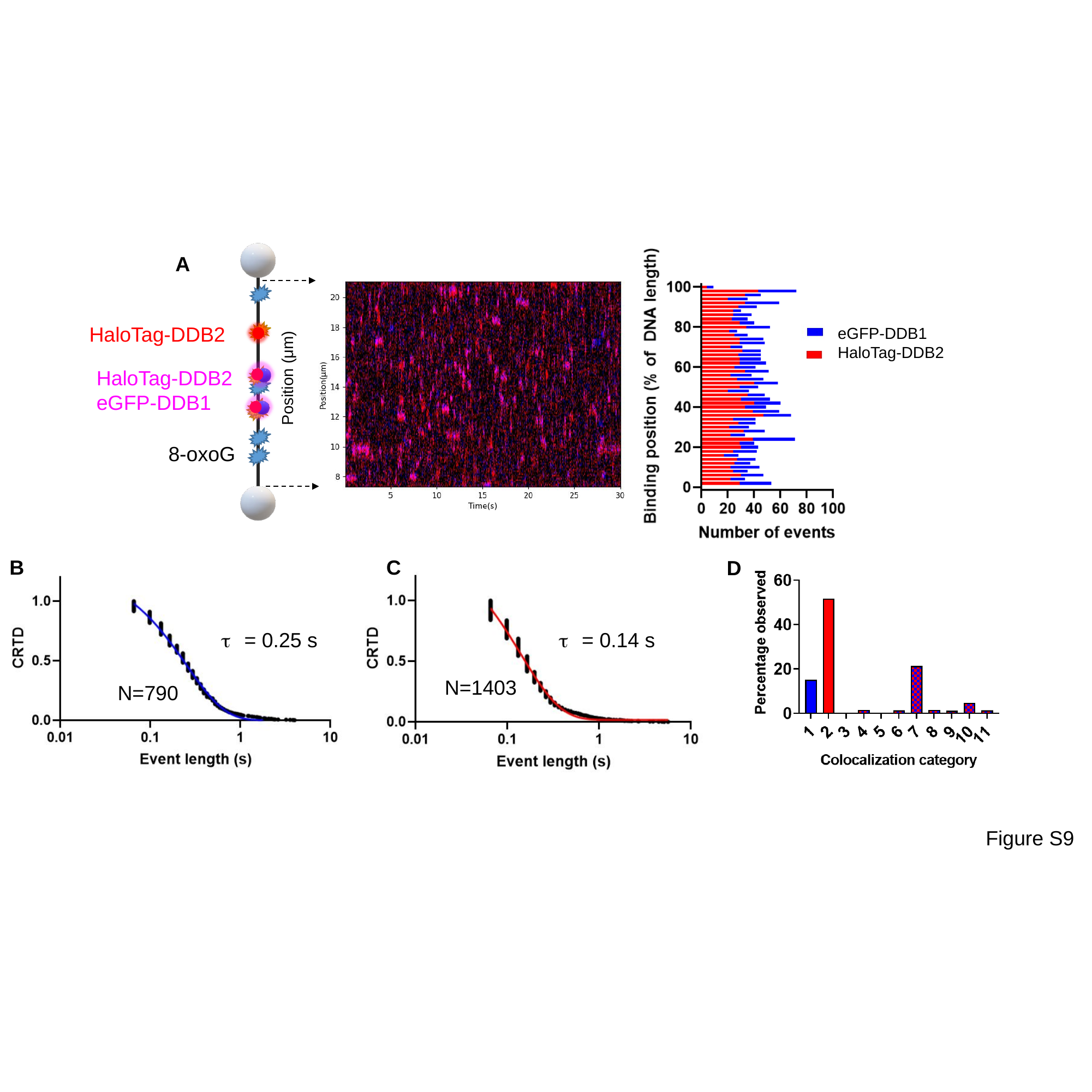

a
HaloTag-DDB2
eGFP-DDB1
HaloTag-DDB2
HaloTag-DDB2
eGFP-DDB1
Position (μm)
8-oxoG
b
c
d
t = 0.25 s
t = 0.14 s
N=1403
N=790
Figure S9

## Slide 21
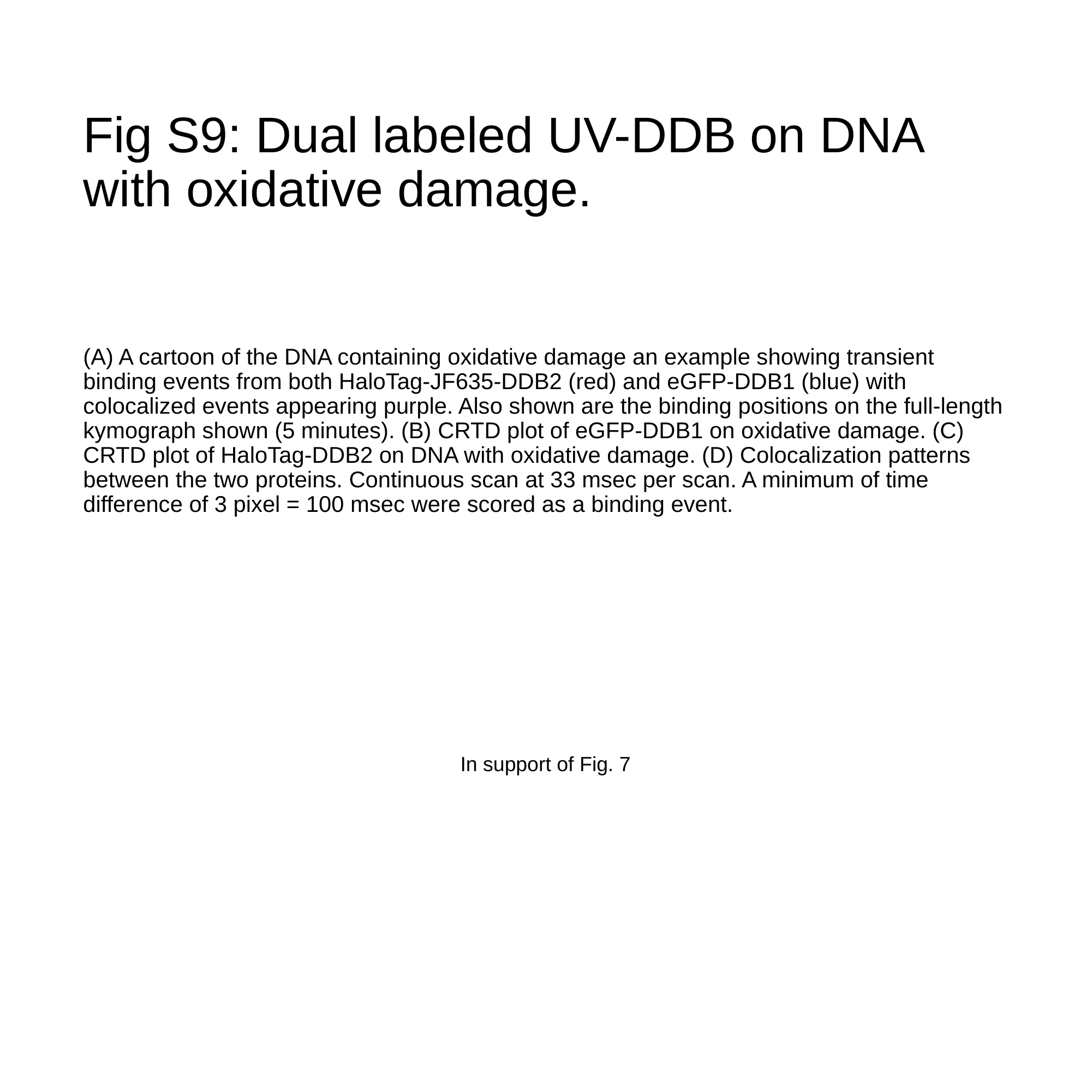

# Fig S9: Dual labeled UV-DDB on DNA with oxidative damage.
(A) A cartoon of the DNA containing oxidative damage an example showing transient binding events from both HaloTag-JF635-DDB2 (red) and eGFP-DDB1 (blue) with colocalized events appearing purple. Also shown are the binding positions on the full-length kymograph shown (5 minutes). (B) CRTD plot of eGFP-DDB1 on oxidative damage. (C) CRTD plot of HaloTag-DDB2 on DNA with oxidative damage. (D) Colocalization patterns between the two proteins. Continuous scan at 33 msec per scan. A minimum of time difference of 3 pixel = 100 msec were scored as a binding event.
In support of Fig. 7
