# Supplementary material for: Single-molecule analysis of DNA-binding proteins from nuclear extracts (SMADNE)
Source: Nucleic Acids Res. 2023 Mar 2;51(7):e39. doi: 10.1093/nar/gkad095 (PMC10123111; doi:10.1093/nar/gkad095)
Supplement: gkad095_Supplemental_Files [file gkad095_supplemental_files.zip › Supplemental tables 1-19-23_fixed.pptx]

## Slide 1
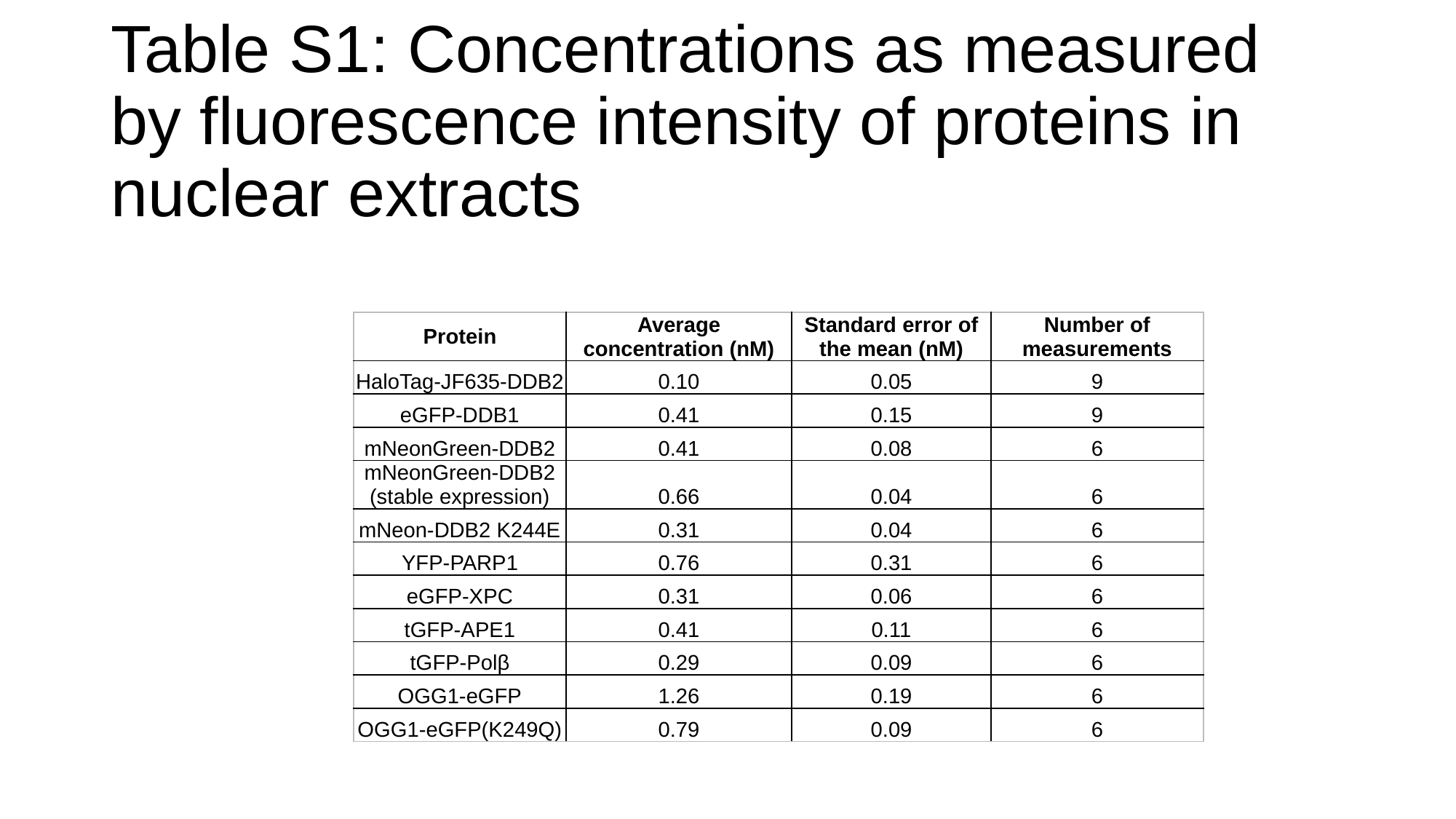

# Table S1: Concentrations as measured by fluorescence intensity of proteins in nuclear extracts
| Protein | Average concentration (nM) | Standard error of the mean (nM) | Number of measurements |
| --- | --- | --- | --- |
| HaloTag-JF635-DDB2 | 0.10 | 0.05 | 9 |
| eGFP-DDB1 | 0.41 | 0.15 | 9 |
| mNeonGreen-DDB2 | 0.41 | 0.08 | 6 |
| mNeonGreen-DDB2 (stable expression) | 0.66 | 0.04 | 6 |
| mNeon-DDB2 K244E | 0.31 | 0.04 | 6 |
| YFP-PARP1 | 0.76 | 0.31 | 6 |
| eGFP-XPC | 0.31 | 0.06 | 6 |
| tGFP-APE1 | 0.41 | 0.11 | 6 |
| tGFP-Polβ | 0.29 | 0.09 | 6 |
| OGG1-eGFP | 1.26 | 0.19 | 6 |
| OGG1-eGFP(K249Q) | 0.79 | 0.09 | 6 |

## Slide 2
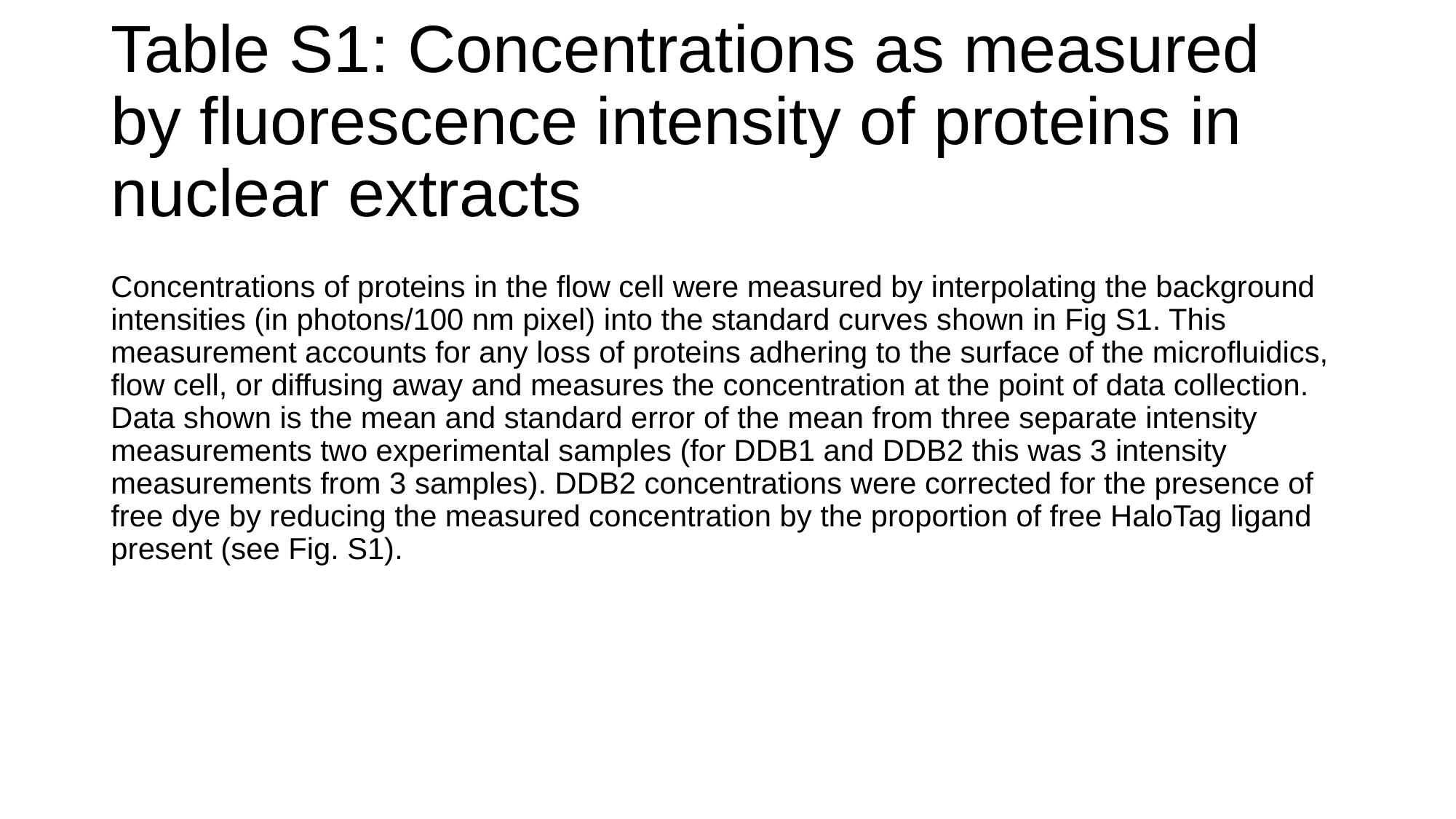

# Table S1: Concentrations as measured by fluorescence intensity of proteins in nuclear extracts
Concentrations of proteins in the flow cell were measured by interpolating the background intensities (in photons/100 nm pixel) into the standard curves shown in Fig S1. This measurement accounts for any loss of proteins adhering to the surface of the microfluidics, flow cell, or diffusing away and measures the concentration at the point of data collection. Data shown is the mean and standard error of the mean from three separate intensity measurements two experimental samples (for DDB1 and DDB2 this was 3 intensity measurements from 3 samples). DDB2 concentrations were corrected for the presence of free dye by reducing the measured concentration by the proportion of free HaloTag ligand present (see Fig. S1).

## Slide 3
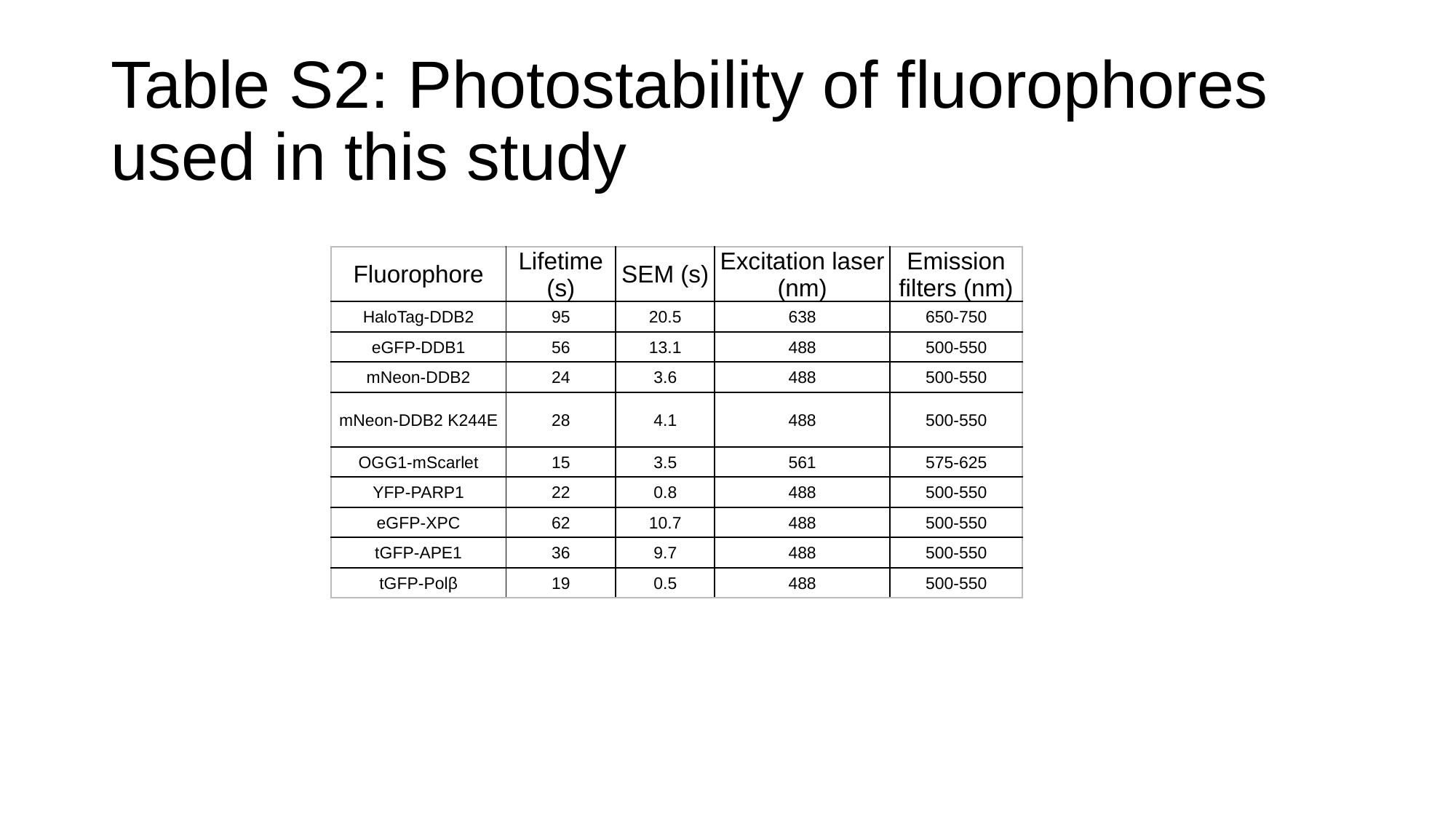

# Table S2: Photostability of fluorophores used in this study
| Fluorophore | Lifetime (s) | SEM (s) | Excitation laser (nm) | Emission filters (nm) |
| --- | --- | --- | --- | --- |
| HaloTag-DDB2 | 95 | 20.5 | 638 | 650-750 |
| eGFP-DDB1 | 56 | 13.1 | 488 | 500-550 |
| mNeon-DDB2 | 24 | 3.6 | 488 | 500-550 |
| mNeon-DDB2 K244E | 28 | 4.1 | 488 | 500-550 |
| OGG1-mScarlet | 15 | 3.5 | 561 | 575-625 |
| YFP-PARP1 | 22 | 0.8 | 488 | 500-550 |
| eGFP-XPC | 62 | 10.7 | 488 | 500-550 |
| tGFP-APE1 | 36 | 9.7 | 488 | 500-550 |
| tGFP-Polβ | 19 | 0.5 | 488 | 500-550 |

## Slide 4
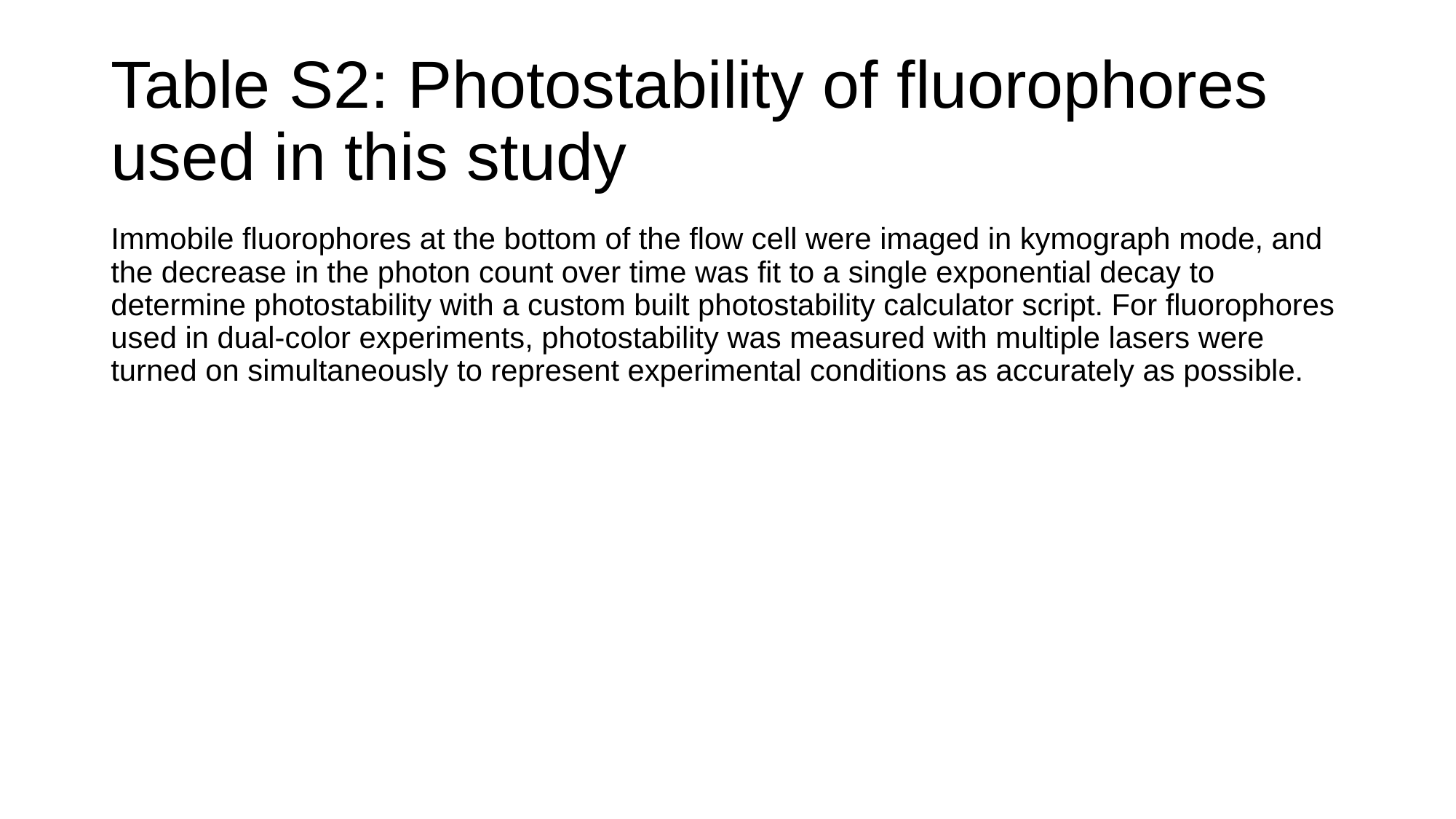

# Table S2: Photostability of fluorophores used in this study
Immobile fluorophores at the bottom of the flow cell were imaged in kymograph mode, and the decrease in the photon count over time was fit to a single exponential decay to determine photostability with a custom built photostability calculator script. For fluorophores used in dual-color experiments, photostability was measured with multiple lasers were turned on simultaneously to represent experimental conditions as accurately as possible.

## Slide 5
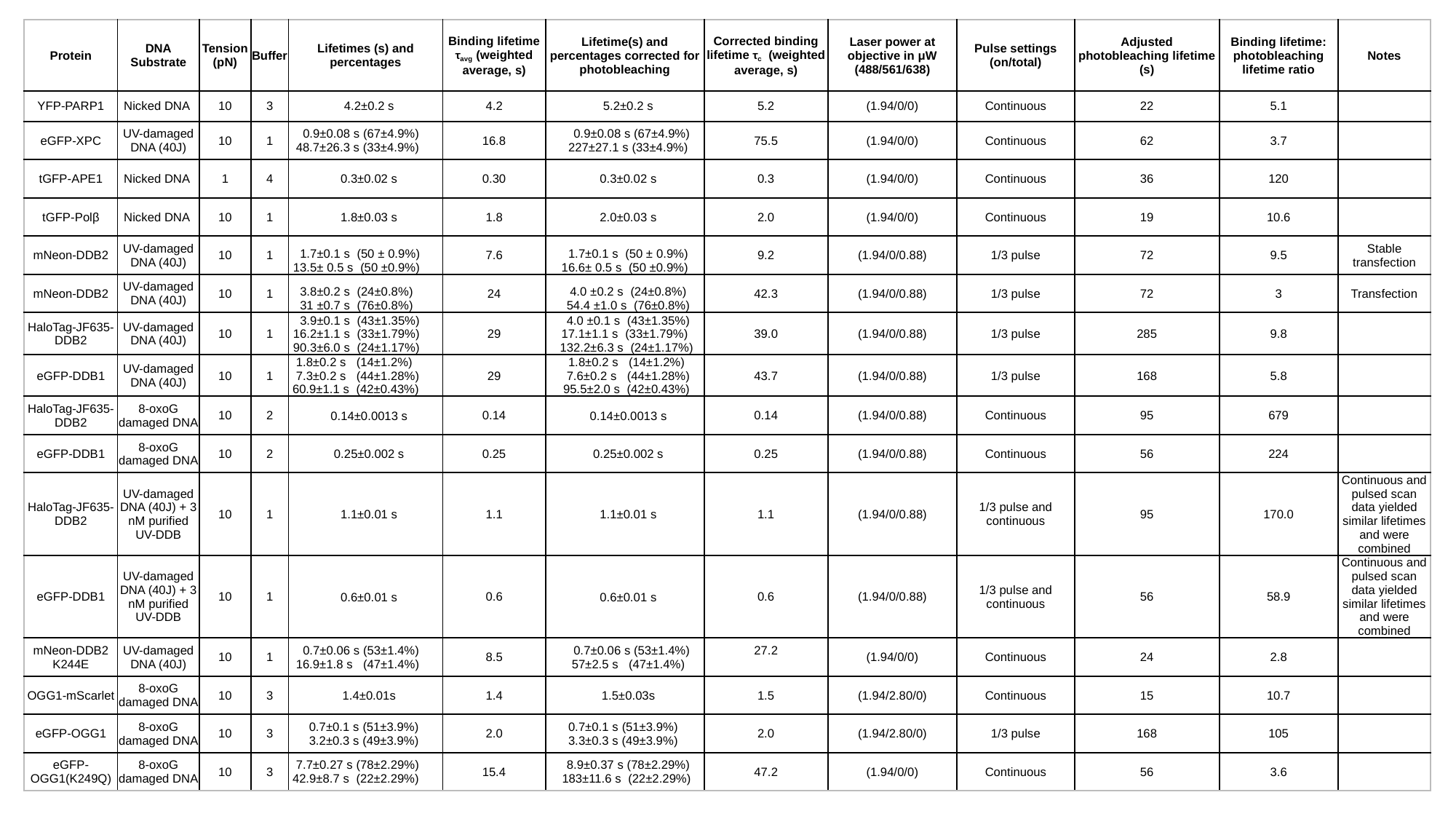

| Protein | DNA Substrate | Tension (pN) | Buffer | Lifetimes (s) and percentages | Binding lifetime τavg (weighted average, s) | Lifetime(s) and percentages corrected for photobleaching | Corrected binding lifetime τc (weighted average, s) | Laser power at objective in μW (488/561/638) | Pulse settings (on/total) | Adjusted photobleaching lifetime (s) | Binding lifetime: photobleaching lifetime ratio | Notes |
| --- | --- | --- | --- | --- | --- | --- | --- | --- | --- | --- | --- | --- |
| YFP-PARP1 | Nicked DNA | 10 | 3 | 4.2±0.2 s | 4.2 | 5.2±0.2 s | 5.2 | (1.94/0/0) | Continuous | 22 | 5.1 | |
| eGFP-XPC | UV-damaged DNA (40J) | 10 | 1 | 0.9±0.08 s (67±4.9%) 48.7±26.3 s (33±4.9%) | 16.8 | 0.9±0.08 s (67±4.9%) 227±27.1 s (33±4.9%) | 75.5 | (1.94/0/0) | Continuous | 62 | 3.7 | |
| tGFP-APE1 | Nicked DNA | 1 | 4 | 0.3±0.02 s | 0.30 | 0.3±0.02 s | 0.3 | (1.94/0/0) | Continuous | 36 | 120 | |
| tGFP-Polβ | Nicked DNA | 10 | 1 | 1.8±0.03 s | 1.8 | 2.0±0.03 s | 2.0 | (1.94/0/0) | Continuous | 19 | 10.6 | |
| mNeon-DDB2 | UV-damaged DNA (40J) | 10 | 1 | 1.7±0.1 s (50 ± 0.9%) 13.5± 0.5 s (50 ±0.9%) | 7.6 | 1.7±0.1 s (50 ± 0.9%) 16.6± 0.5 s (50 ±0.9%) | 9.2 | (1.94/0/0.88) | 1/3 pulse | 72 | 9.5 | Stable transfection |
| mNeon-DDB2 | UV-damaged DNA (40J) | 10 | 1 | 3.8±0.2 s (24±0.8%) 31 ±0.7 s (76±0.8%) | 24 | 4.0 ±0.2 s (24±0.8%) 54.4 ±1.0 s (76±0.8%) | 42.3 | (1.94/0/0.88) | 1/3 pulse | 72 | 3 | Transfection |
| HaloTag-JF635-DDB2 | UV-damaged DNA (40J) | 10 | 1 | 3.9±0.1 s (43±1.35%) 16.2±1.1 s (33±1.79%) 90.3±6.0 s (24±1.17%) | 29 | 4.0 ±0.1 s (43±1.35%) 17.1±1.1 s (33±1.79%) 132.2±6.3 s (24±1.17%) | 39.0 | (1.94/0/0.88) | 1/3 pulse | 285 | 9.8 | |
| eGFP-DDB1 | UV-damaged DNA (40J) | 10 | 1 | 1.8±0.2 s (14±1.2%) 7.3±0.2 s (44±1.28%) 60.9±1.1 s (42±0.43%) | 29 | 1.8±0.2 s (14±1.2%) 7.6±0.2 s (44±1.28%) 95.5±2.0 s (42±0.43%) | 43.7 | (1.94/0/0.88) | 1/3 pulse | 168 | 5.8 | |
| HaloTag-JF635-DDB2 | 8-oxoG damaged DNA | 10 | 2 | 0.14±0.0013 s | 0.14 | 0.14±0.0013 s | 0.14 | (1.94/0/0.88) | Continuous | 95 | 679 | |
| eGFP-DDB1 | 8-oxoG damaged DNA | 10 | 2 | 0.25±0.002 s | 0.25 | 0.25±0.002 s | 0.25 | (1.94/0/0.88) | Continuous | 56 | 224 | |
| HaloTag-JF635-DDB2 | UV-damaged DNA (40J) + 3 nM purified UV-DDB | 10 | 1 | 1.1±0.01 s | 1.1 | 1.1±0.01 s | 1.1 | (1.94/0/0.88) | 1/3 pulse and continuous | 95 | 170.0 | Continuous and pulsed scan data yielded similar lifetimes and were combined |
| eGFP-DDB1 | UV-damaged DNA (40J) + 3 nM purified UV-DDB | 10 | 1 | 0.6±0.01 s | 0.6 | 0.6±0.01 s | 0.6 | (1.94/0/0.88) | 1/3 pulse and continuous | 56 | 58.9 | Continuous and pulsed scan data yielded similar lifetimes and were combined |
| mNeon-DDB2 K244E | UV-damaged DNA (40J) | 10 | 1 | 0.7±0.06 s (53±1.4%) 16.9±1.8 s (47±1.4%) | 8.5 | 0.7±0.06 s (53±1.4%) 57±2.5 s (47±1.4%) | 27.2 | (1.94/0/0) | Continuous | 24 | 2.8 | |
| OGG1-mScarlet | 8-oxoG damaged DNA | 10 | 3 | 1.4±0.01s | 1.4 | 1.5±0.03s | 1.5 | (1.94/2.80/0) | Continuous | 15 | 10.7 | |
| eGFP-OGG1 | 8-oxoG damaged DNA | 10 | 3 | 0.7±0.1 s (51±3.9%) 3.2±0.3 s (49±3.9%) | 2.0 | 0.7±0.1 s (51±3.9%) 3.3±0.3 s (49±3.9%) | 2.0 | (1.94/2.80/0) | 1/3 pulse | 168 | 105 | |
| eGFP-OGG1(K249Q) | 8-oxoG damaged DNA | 10 | 3 | 7.7±0.27 s (78±2.29%) 42.9±8.7 s (22±2.29%) | 15.4 | 8.9±0.37 s (78±2.29%) 183±11.6 s (22±2.29%) | 47.2 | (1.94/0/0) | Continuous | 56 | 3.6 | |

## Slide 6
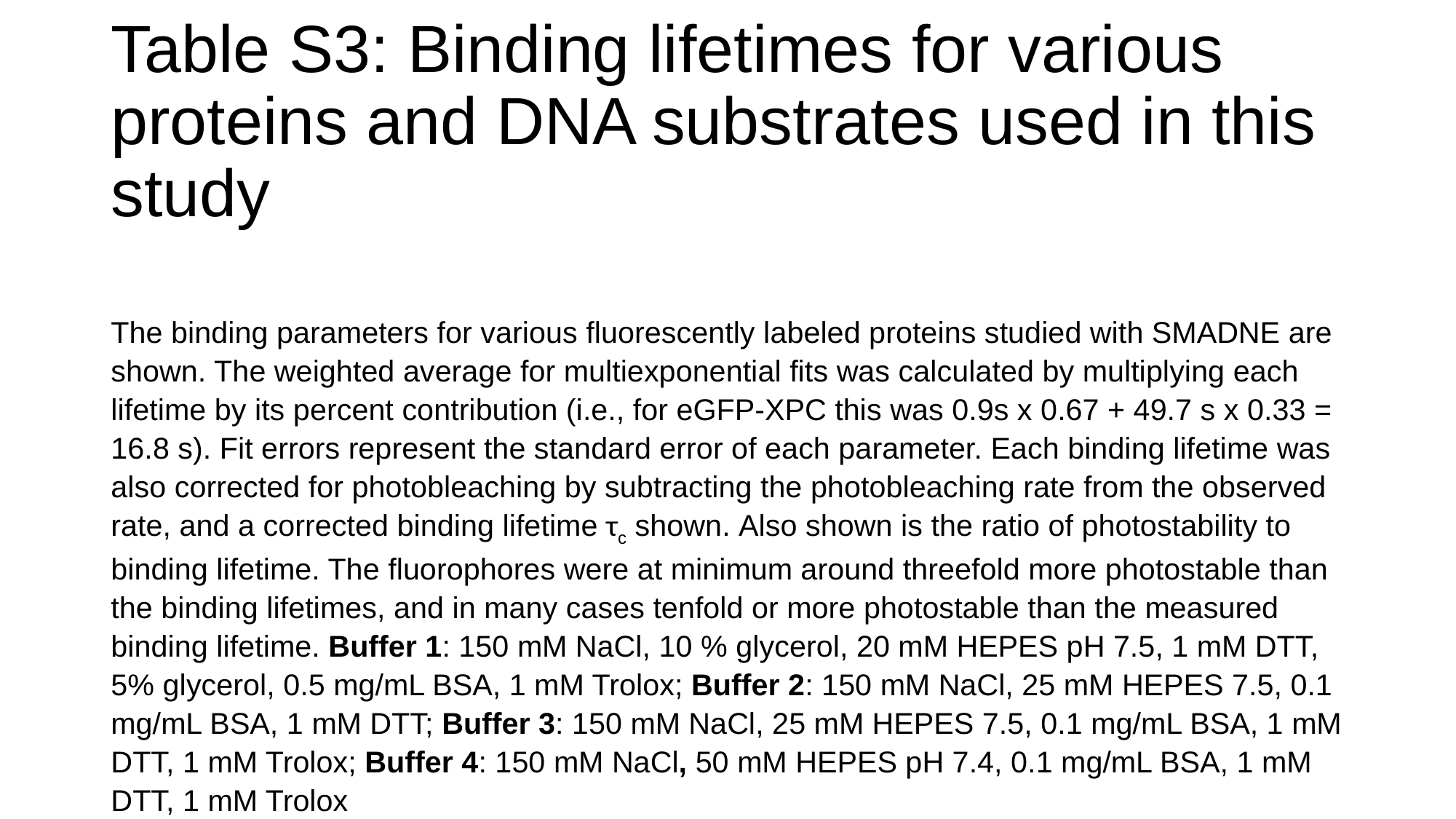

# Table S3: Binding lifetimes for various proteins and DNA substrates used in this study
The binding parameters for various fluorescently labeled proteins studied with SMADNE are shown. The weighted average for multiexponential fits was calculated by multiplying each lifetime by its percent contribution (i.e., for eGFP-XPC this was 0.9s x 0.67 + 49.7 s x 0.33 = 16.8 s). Fit errors represent the standard error of each parameter. Each binding lifetime was also corrected for photobleaching by subtracting the photobleaching rate from the observed rate, and a corrected binding lifetime τc shown. Also shown is the ratio of photostability to binding lifetime. The fluorophores were at minimum around threefold more photostable than the binding lifetimes, and in many cases tenfold or more photostable than the measured binding lifetime. Buffer 1: 150 mM NaCl, 10 % glycerol, 20 mM HEPES pH 7.5, 1 mM DTT, 5% glycerol, 0.5 mg/mL BSA, 1 mM Trolox; Buffer 2: 150 mM NaCl, 25 mM HEPES 7.5, 0.1 mg/mL BSA, 1 mM DTT; Buffer 3: 150 mM NaCl, 25 mM HEPES 7.5, 0.1 mg/mL BSA, 1 mM DTT, 1 mM Trolox; Buffer 4: 150 mM NaCl, 50 mM HEPES pH 7.4, 0.1 mg/mL BSA, 1 mM DTT, 1 mM Trolox
